# Time‐varying age‐ and CD4‐stratified rates of mortality and WHO stage 3 and stage 4 events in children, adolescents and youth 0 to 24 years living with perinatally acquired HIV, before and after antiretroviral therapy initiation in the paediatric IeDEA Global Cohort Consortium

**DOI:** 10.1002/jia2.25617

**Published:** 2020-10-09

**Authors:** Sophie Desmonde, Anne M Neilan, Beverly Musick, Gabriela Patten, Kulkanya Chokephaibulkit, Andrew Edmonds, Stephany N Duda, Karen Malateste, Kara Wools‐Kaloustian, Andrea L Ciaranello, Mary‐Ann Davies, Valériane Leroy, IeDEA

**Affiliations:** ^1^ Inserm U1027 Université Paul Sabatier Toulouse 3 Toulouse France; ^2^ Division of General Academic Pediatrics Department of Pediatrics Massachusetts General Hospital for Children Boston MA USA; ^3^ School of Medicine Indiana University Indianapolis IN USA; ^4^ School of Public Health and Family Medicine Faculty of Health Sciences University of Cape Town Cape Town South Africa; ^5^ Faculty of Medicine Siriraj Hospital Mahidol University Bangkok Thailand; ^6^ Department of Epidemiology Gillings School of Global Public Health The University of North Carolina at Chapel Hill Chapel Hill NC USA; ^7^ Department of Biomedical Informatics Vanderbilt University Medical Center Nashville TN USA; ^8^ Inserm U1219 Université de Bordeaux Bordeaux France; ^9^ Bordeaux Population Health Center Université de Bordeaux Bordeaux France; ^10^ Division of Infectious Diseases Department of Medicine Massachusetts General Hospital Boston MA USA

**Keywords:** HIV, paediatrics, adolescents, mortality, severe morbidity

## Abstract

**Introduction:**

Evaluating outcomes of paediatric patients with HIV provides crucial data for clinicians and policymakers. We analysed mortality and clinical events rates among children, adolescents, and youth with perinatally acquired HIV (PHIV) aged 0 to 24 years stratified by time‐varying age and CD4, before and after antiretroviral therapy (ART), in the paediatric IeDEA multiregional collaboration (East, West, Central and Southern Africa, Asia‐Pacific, and Central/South America and the Caribbean).

**Methods:**

ART‐naïve children with HIV enrolled before age 10 (proxy for perinatal infection) at IeDEA sites between 2004 and 2016, with ≥1 CD4 measurement during follow‐up were included. We estimated incidence rates (IR) and 95% confidence intervals (95% CI) of mortality and first occurrence of WHO‐4 and WHO‐3 events, excluding tuberculosis, during person‐years (PY) spent within different age (<2, 2 to 4, 5 to 9, 10 to 14, 15 to 19, 20 to 24) and CD4 (percent when <5 years [<15%, 15% to 24%, ≥25%]; count when ≥5 years [<200, 200 to 499, ≥500 cells/µL]) strata. We used linear mixed models to predict CD4 evolution, with trends modelled by region.

**Results:**

In the pre‐ART period, 49 137 participants contributed 51 966 PY of follow‐up (median enrolment age: 3.9 years). The overall pre‐ART IRs were 2.8/100 PY (95% CI: 2.7 to 2.9) for mortality, 3.3/100 PY (95% CI: 3.0 to 3.5) for first occurrence of a WHO‐4 event, and 7.0/100 PY (95% CI: 6.7 to 7.4) for first occurrence of a WHO‐3 event. Lower CD4 and younger age strata were associated with increased rates of both mortality and first occurrence of a clinical event. In the post‐ART period, 52 147 PHIVY contributed 207 945 PY (ART initiation median age: 4.5 years). Overall mortality IR was 1.4/100 PY (95% CI: 1.4 to 1.5) and higher in low CD4 strata; patients at each end of the age spectrum (<2 and >19) had increased mortality post‐ART. IRs for first occurrence of WHO‐4 and WHO‐3 events were 1.3/100 PY (95% CI: 1.2 to 1.4) and 2.1/100 PY (95% CI: 2.0 to 2.2) respectively. These were also associated with lower CD4 and younger age strata.

**Conclusions:**

Mortality and incidence of clinical events were highest in both younger (<2 years) and older (>19 years) youth with PHIV. Scaling‐up services for <2 years (early access to HIV diagnosis and care) and >19 years (adolescent‐ and youth‐focused health services) is critical to improve outcomes among PHIVY.

## 1 INTRODUCTION

With the rollout of antiretroviral therapy (ART), children living with perinatally acquired HIV (PHIV) are now surviving into adolescence and young adulthood [1, 2, 3]. In 2016, of the 1.3 million adolescents ages 15 to 19 living with HIV, over a third acquired HIV perinatally. Globally, adolescents experience poorer health outcomes compared to adults with HIV [4, 5, 6, 7]. Updated data detailing rates of mortality and clinical events among people with PHIV are essential to both clinicians and policymakers [5, 7, 8]. Understanding current rates of important clinical events, including mortality, for children, adolescents and youth living with PHIV in low‐income settings as they age is critical, and this understanding should inform interventions designed for this vulnerable population.

The International epidemiology Databases to Evaluate AIDS (IeDEA) research consortium (https://www.iedea.org/) harmonizes globally diverse HIV/AIDS data from routine clinical care. The objective of this study was to describe the incidence of mortality and first occurrence of WHO‐4 and WHO‐3 events stratified by time‐updated age and CD4 before and after ART initiation among a large, multiregional IeDEA cohort of children, adolescents and youth living with (PHIVY).

## 2 METHODS

### 2.1 Study population, participants and data collection

Individual patient data from the six paediatric cohorts within IeDEA were pooled: Asia‐Pacific; West Africa; East Africa; Central Africa; Southern Africa; and the Caribbean, Central and South America Network (CCASAnet) [9]. Study inclusion criteria included enrolment in care before 10 years of age (as a proxy for perinatal infection) at any participating IeDEA site between 2004 and 2016; confirmed HIV diagnosis; being ART‐naïve at enrolment; and, having at least one CD4 measurement (count or percent) during follow‐up.

Data abstracted for this analysis were generated during routine care encounters and included region, country, site, demographics (sex, date of birth, HIV diagnosis date [when available], and date of enrolment in care), laboratory values (CD4 count/percent), ART regimens, and, if applicable, date of last clinical contact, transfer out or death.

All sites contributed to the mortality analyses, and a subset of sites contributed to the clinical events analyses. For the clinical events analyses, regional data managers assessed the reliability and completeness of data using a 6‐item survey, which gathered data on the frequency and details of event data collected at each site. Sites were selected based on reporting in both the pre‐ and post‐ART periods. Overall, 27/61 IeDEA sites that care for children were included in the clinical events analyses; all regions contributed at least one site, except West Africa, where sites do not record clinical diagnoses.

Diagnoses recorded in regional databases were derived by the clinician treating the patient utilizing clinical assessments and locally available diagnostic testing, including clinical, radiographic, laboratory and pathological evaluations. Clinical events were categorized by 2007 WHO staging criteria as Stage 4 (WHO‐4) or Stage 3 (WHO‐3)+++, excluding tuberculosis (TB) [10]. While technologies such as Xpert MTB/RIF are being implemented in some IeDEA sites, most sites rely on presumptive clinical diagnoses for TB [11]. Given widespread variations in these diagnosis and documentation practices [12], we excluded TB from the primary analyses to avoid biased estimates. We conducted a separate analysis evaluating the incidence of TB events; importantly, reported diagnoses were not supported by any additional data.

The data presented here are based on retrospective de‐identified information collected on a routine basis in sites participating in the IeDEA consortium. These data were approved for use by the local institutional review boards in each of the IeDEA countries included in the analysis and consent requirements were deferred to the local institutional review boards. Furthermore, the research did not include any planned intervention by the researcher or direct interaction with individuals or groups and no results can identify specific individuals.

### 2.2 Outcomes and key definitions

Outcomes included mortality, first occurrence of WHO‐4 event, and first occurrence of WHO‐3 event, excluding TB, during follow‐up (Appendix, Data S1). We defined two distinct follow‐up periods: (i) the pre‐ART period, from enrolment until 30 days after ART initiation (or loss to follow‐up [LTFU], transfer or death, whichever came first) and (ii) the post‐ART period, from the 31st day on ART until database closure date (or LTFU, transfer, or death, whichever came first). Events occurring in the first 30 days after ART initiation were included in the pre‐ART period, to account for the likelihood that such events likely represented unmasking of a previously existing condition. We limited this period to 30 days to avoid diluting high rates of mortality and infectious morbidity previously reported in the pre‐universal ART era [2, 13]. LTFU was defined as having no clinical contact for ≥6 months since the last visit recorded in the database in the pre‐ART period and for ≥3 months in the post‐ART period; in case of LTFU, follow‐up was censored at date of last clinical contact. Those still in care at database closure were also censored at date of last visit.

Baseline was defined as date of enrolment in the pre‐ART period and date of ART initiation in the post‐ART period.

### 2.3 Statistical analyses

Time varying age was divided in six stratums (<2, 2 to 4, 5 to 9, 10 to 14, 15 to 19, and 20 to 24 years) and CD4 levels were divided into three stratums: we used exclusively CD4 percentage in children aged <5 years (<15%, 15% to 24%, ≥25%) and absolute CD4 cell count for older children aged ≥5 years (<200, 200 to 499, ≥500 cells/µL). First, we estimated the incidence rates (IRs), events per 100 person‐years (PY) of follow‐up, of mortality stratified by the combination of time‐varying age and CD4 level, and conducted a sensitivity analysis where all LTFU was considered as underreported mortality. Second, we estimated IR/100 PYs for the first occurrence of WHO‐4 and WHO‐3 events, also stratified by time‐varying age and CD4 in a subset of sites. We report IRs separately in the pre‐ART and post‐ART periods, given that some sites only provided care in the post‐ART periods.

To calculate the total person‐time contributed by participants in each age/CD4 stratum, we used random effect models and used estimated dates when strata thresholds were crossed. If only one CD4 value was available and the follow‐up duration was <6 months, we carried this CD4 value through an individual’s entire follow‐up time. If a participant had >1 CD4 value or follow‐up duration ≥6 months, we estimated the date when strata thresholds were crossed based on the overall CD4 pattern observed among the participants in their region. Pre‐ART, we estimated the CD4 decline since birth (the estimated time of infection) using linear mixed models. Post‐ART, we estimated the CD4 increase since ART initiation using linear mixed models. We modelled an increased slope for the first six months post‐ART initiation, reflecting the expected initial robust immune response pattern post‐ART [14, 15]. If a patient only had one CD4 measurement available, dates at which CD4 thresholds were crossed were based on the average slope of CD4 decline (pre ART) or increase (post ART) for the cohort. Separate models were built for each IeDEA region.

We calculated IRs as the number of events divided by the total number of PY of follow‐up for each age and year stratum, and computed confidence intervals (CIs) for Poisson means using normal approximation, based on the logarithmic transformation of the rates. We assessed IR trends across all strata using Poisson models for trend.

## 3 RESULTS

Overall, 65 903 children, adolescents, and youth were included, contributing a total of 259 912 person‐years of follow‐up; 49 137 participants contributed to the pre‐ART period and 52 147 to the post‐ART period (Table 1).

**Table 1:** Characteristics of IeDEA participants at baseline and during follow‐up

Characteristic	Pre‐ART participants	Post‐ART participants
Region, n (%)		
Asia‐Pacific	3450 (7)	3755 (7)
CCASAnet	979 (2)	1252 (2)
Central Africa	1538 (3)	1564 (3)
East Africa	9742 (20)	8344 (16)
Southern Africa	29 649 (60)	33 321 (64)
West Africa	3779 (8)	3911 (8)
Overall	49 137 (100)	52 147 (100)
Person‐time, years (%)		
Asia‐Pacific	5372 (10)	21 833 (11)
CCASAnet	1460 (3)	8716 (4)
Central Africa	2901 (6)	7449 (4)
East Africa	13 754 (27)	30 279 (15)
Southern Africa	24 454 (47)	121 596 (59)
West Africa	4024 (8)	18 073 (9)
Overall	51 967 (100)	207 945 (100)

Baseline	At enrolment	At ART initiation
Year, n (%)		
<2004	2065 (4)	2686 (5)
2004 to 2007	18 781 (38)	18 921 (36)
2008 to 2012	20 858 (43)	22 086 (42)
2013 to 2014	5153 (11)	5993 (11)
2015 to 2016	2280 (5)	2461 (5)
Female, n (%)	24 897 (51)	25 871 (50)
Pre‐ART follow‐up, n (%)		35 381 (68)
Age, median (IQR), years	3.9 (1.5 to 6.7)	4.5 (1.9 to 7.4)
Outcomes		
Initiated ART, n (%)	37 591 (77)	
Deceased, n (%)	1449 (3)	2932 (6)
Lost to follow‐up,^a^ n (%)	8063 (16)	20 600 (40)

ART, antiretroviral therapy; CCASAnet, the Caribbean, Central and South American Network; IQR, interquartile range.

^a^ Lost to follow‐up in the pre‐ART period was defined as 6 months since last clinical contact and in the post‐ART period as 3 months since last clinical contact.

During the pre‐ART period, 49 137 participants contributed 51 967 PY (87% from sub‐Saharan Africa, 10% from the Asia‐Pacific, and 3% from CCASAnet). Median age at enrolment was 3.9 years (interquartile range [IQR]: 1.5 to 6.7); 51% were female and >80% enrolled between 2004 and 2012. Overall median follow‐up was 4.0 months [IQR: 1.8 to 13.0] and 77% initiated ART. More person‐time was spent in the 5 to 9 years age stratum (48%) than any other age stratum, and 52% of person‐time was spent in the highest CD4 stratum (Table 2).

**Table 2:** Distribution of person‐time by time‐updated age and CD4 count (or CD4% if <5 years) in both the pre‐ART and the post‐ART periods

Person‐time, years (%), by time‐updated age and CD4 strata								
Age	Pre‐ART participants				Post‐ART participants			
	CD4 cell %/count				CD4 cell %/count			
	<15%	15% to 24%	≥25%	Overall	<15%	15% to 24%	≥25%	Overall
	<200 cells/µL	200 to 499 cells/µL	≥500 cells/µL		<200 cells/µL	200 to 499 cells/µL	≥500 cells/µL	
<2 years	1171 (2)	3210 (6)	2487 (5)	6868 (13)	727 (0)	3391 (2)	5970 (3)	10 087 (5)
2 to 4 years	1842 (4)	6443 (12)	4915 (9)	13 200 (25)	1703 (1)	8335 (4)	28 066 (13)	38 103 (18)
5 to 9 years	1629 (3)	7318 (14)	16 134 (31)	25 081 (48)	2902 (1)	12 041 (6)	75 815 (36)	90 758 (44)
10 to 14 years	368 (1)	2727 (5)	3391 (7)	6485 (12)	2027 (1)	12 030 (6)	44 240 (21)	58 297 (28)
15 to 19 years	39 (0)	159 (0)	133 (0)	330 (1)	749 (0)	3047 (1)	6604 (3)	10 400 (5)
20 to 24 years	0 (0)	1 (0)	2 (0)	2 (0)	53 (0)	76 (0)	171 (0)	300 (0)
Overall	5049 (10)	19 857 (38)	27 060 (52)	51 967 (100)	8160 (4)	38 920 (19)	160 866 (77)	207 946 (100)

Overall column and line totals may not sum to overall total due to rounding of person‐time years. ART, antiretroviral therapy.

During the post‐ART period, 52 147 participants contributed 207 945 PY. Of note, 68% of these participants also contributed pre‐ART person‐time. Of the entire cohort, 32% were enrolled in care on the day of ART initiation. More than half (59%) were from Southern Africa, 50% were female, and the median age at ART initiation was 4.5 years (IQR: 1.9 to 7.4) (Table 1). Similar to the pre‐ART period, most person‐time was spent between the ages of 5 and 9 years (44%), and 77% was spent in the highest CD4 stratum (Table 2). Median follow‐up time was 42 months (IQR: 17 to 74).

### 3.1 Mortality incidence in the pre‐ART period

**Figure 1:**
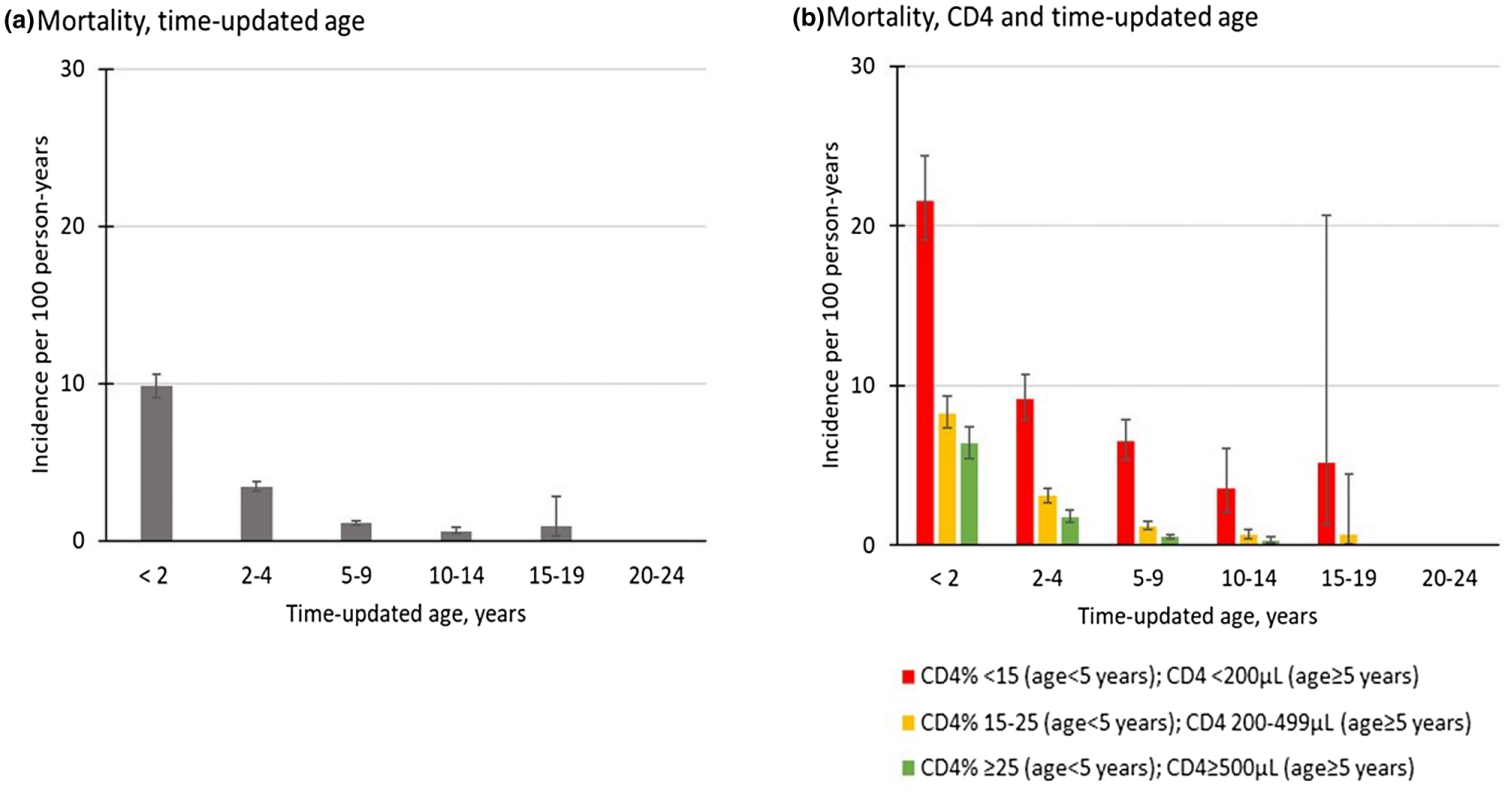
Incidence rates of pre‐ART mortality by (**a**) time updated age, and (**b**) CD4 and time updated age, IeDEA multiregional cohort, 2004 to 2016 . Error bars indicate 95% confidence intervals.

Overall, 1449 deaths were recorded pre‐ART; the overall mortality rate was 2.8/100 PY (95% CI: 2.7 to 2.9). Incidence of mortality was highest in children <2 years (9.8/100 PY, 95% CI: 9.1 to 10.6, Figure 1a) and decreased as age increased, reaching 0.6/100 PY (95% CI: 0.5 to 0.8) in adolescents aged 10 to 14 years (*p*‐value for trend, based on Poisson model: *p* < 0.001). We then observed a slight uptick in mortality within the 15 to 19 stratum (Figure A, Tables A and C, Data S1). When stratified by CD4 and age, mortality was highest in the lowest CD4 stratum and the youngest age stratum, 14.0/100 PY (95% CI: 12.7 to 15.4) in children <5 years with CD4 < 15% (Figure 1b, Tables B and C, Data S1). In sensitivity analyses, including LTFU as mortality, the overall mortality rate reached 19.0/100 PY (95% CI: 18.6 to 19.3) with similar trends including the marked increase in IR among those aged 15 to 19 years (Tables A and D, Data S1).

### 3.2 Incidence of first occurrence of WHO‐4 and WHO‐3 events in the pre‐ART period

Of the 49 137 participants with pre‐ART follow‐up, a subset of 16 596 (33%) children contributed to the analyses of incidence of WHO‐4 and WHO‐3 events.

**Figure 2:**
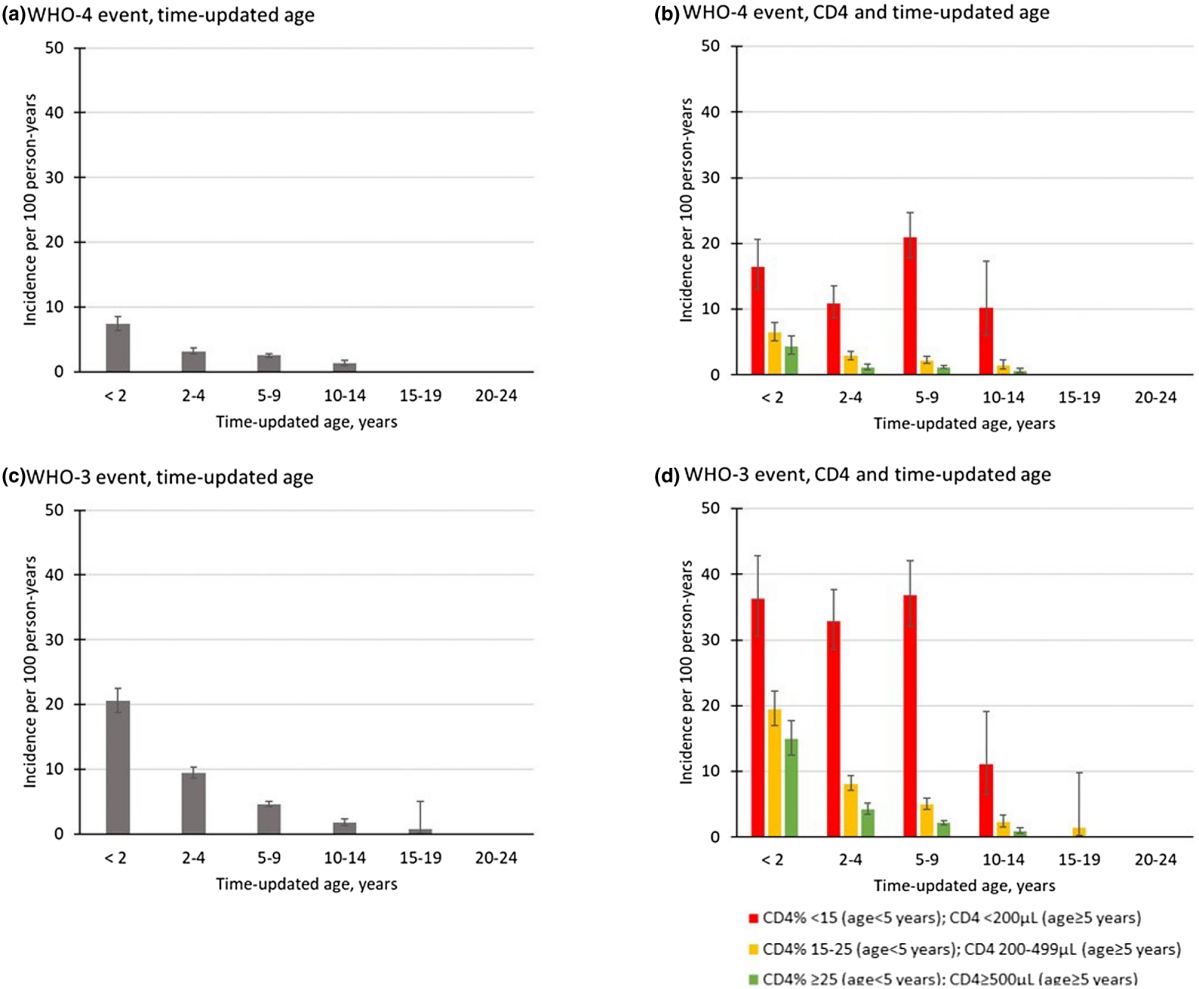
Incidence rates of pre‐ART WHO‐4 event by (**a**) time‐updated age, and (**b**) CD4 and time‐updated age, and WHO‐3 event by (**c**) time‐updated age, and (**d**) CD4 and time‐updated age IeDEA multiregional cohort, 2004 to 2016. Error bars indicate 95% confidence intervals.

Of the 16 596 participants included in the clinical event analysis, 424 (3%) presented with a WHO‐4 event at enrolment and were excluded from the analyses of incident first WHO‐4 events. The 16 172 remaining children contributed 23 883 PY and 723 (4%) had at least one WHO‐4 event during pre‐ART follow‐up. Pneumocystis pneumonia (21%) constituted the most frequent diagnosis (Table E, Figure B, Data S1). The overall IR for first occurrence of WHO‐4 event was 3.3/100 PY (95% CI: 3.0 to 3.5). The highest IR was observed in the stratum of lowest CD4 levels and youngest ages (<2 years with CD4 < 15%: 16.4/100 PY; 95% CI: 13.0 to 20.7); the lowest IR was observed among those 10 to 14 years with CD4 ≥ 500 (0.5/100 PY; 95% CI: 0.3 to 1.0; *p* < 0.001) (Figure 2a and b, Table F, Data S1).

Of the 16 596 participants, 1430 (9%) had a prevalent WHO‐3 clinical event at enrolment and were excluded from the analyses of incident WHO‐3 events. Among the remaining 15 166 children, 1528 WHO‐3 events occurred during 21 731 PY of follow‐up (7.0/100 PY, 95% CI: 6.7 to 7.4). Oral candidiasis constituted 46% of the diagnoses (Figure B, Data S1). We observed the highest IR in the stratum of lowest CD4 levels and youngest ages (<2 years with CD4 < 15%: 36.2/100 PY; 95% CI: 30.6 to 42.9), and the lowest IR among those 10 to 14 years with CD4 ≥ 500: 0.9/100 PY; 95% CI: 0.5 to 1.4; *p* < 0.001) (Figures 2c and d, Table G, Data S1).

Overall, we observed higher IRs for both WHO‐4 and WHO‐3 clinical events in the Asia‐Pacific and CCASAnet regions compared to sub‐Saharan Africa, (Figures C and D, Tables E and G, Data S1).

Additionally, we reported 1414 TB events occurring among 15 881 children at risk (IR 6.2/100 PY, 95% CI: 6.0 to 6.6). Rates were significantly higher in younger children in and among those with low CD4 counts (Table H of Data S1).

### 3.3 Mortality incidence in the post‐ART period

**Figure 3:**
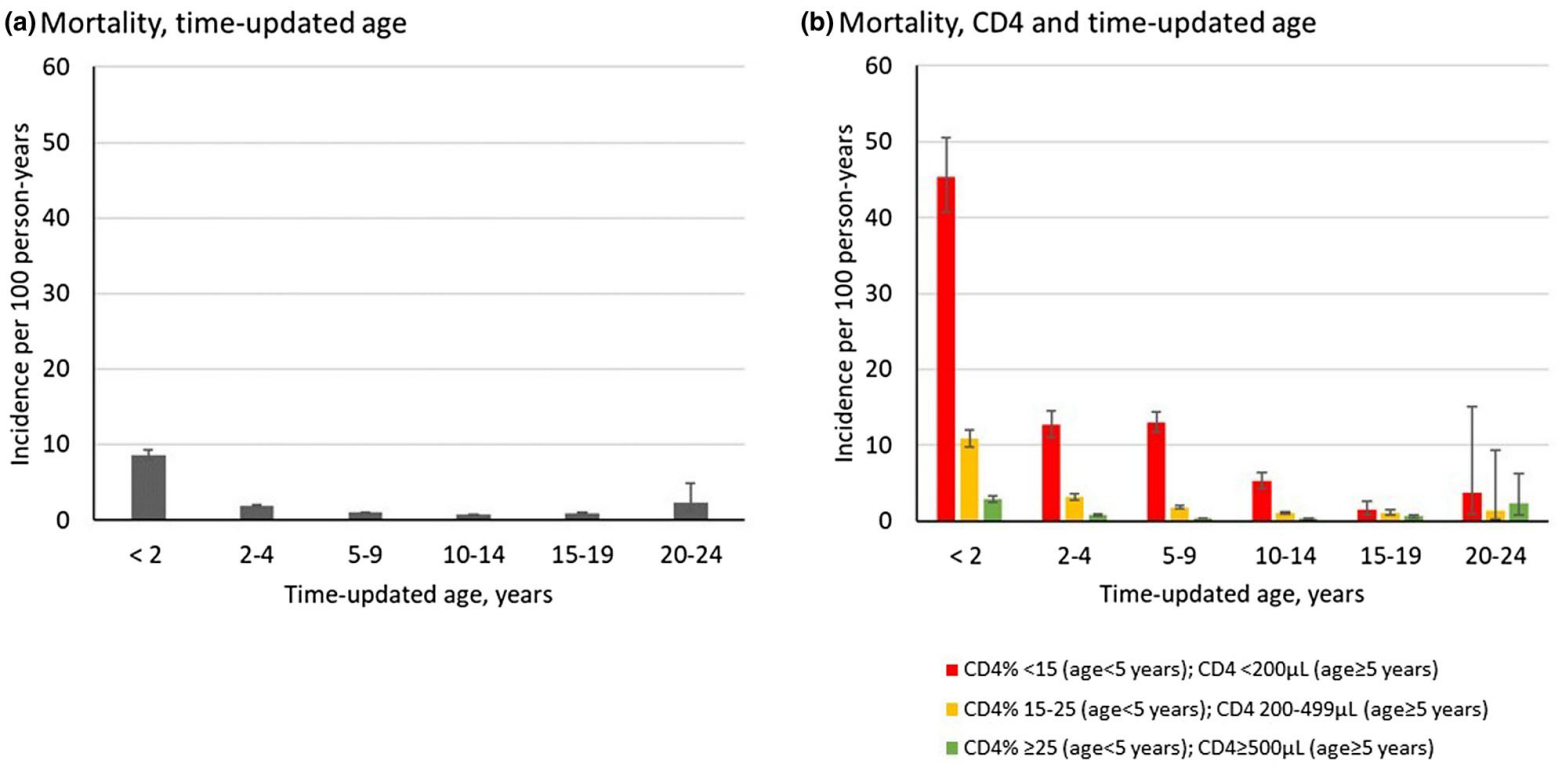
Incidence rates of post ART mortality by (**a**) time updated age, and (**b**) CD4 and time updated age, IeDEA multiregional cohort, 2004 to 2016. Error bars indicate 95% confidence intervals.

Overall, 2932 deaths (6%) were recorded post‐ART; the overall post‐ART mortality IR was 1.4/100 PY (95% CI: 1.4 to 1.5). Similar to the pre‐ART period, mortality was highest within the < 2 years stratum (<2 years: 8.6/100 PY; 95% CI: 8.1 to 9.2). Mortality then followed a decreasing trend within the middle age strata and then increased within the older ages (Figure 3a, Table A, Data S1). In all age groups, mortality was highest in the lowest CD4 stratum (Figure 3b). Furthermore, in this stratum, in children <2 years, post‐ART mortality was more than 2‐fold higher than pre‐ART mortality IR (45.4/100 PY; 95% CI: 40.8 to 50.6, compared to 21.6/100 PY; 95% CI: 19.1 to 24.4). Higher post‐ART mortality in the lowest CD4 stratum was observed in all age groups. Overall, mortality IRs did not differ by region; detailed results are available in Figure E and Table I, Data S1. In sensitivity analysis the overall mortality rate reached 12.0/100 PY (95% CI: 11.9 to 12.2); we observed the same trends as in the primary analysis (Table A, Table J, Data S1).

### 3.4 Incidence of first occurrence of WHO‐4 and WHO‐3 events in the post‐ART period

In the post‐ART period, a subset of 16 079 participants contributed to the WHO‐4 and WHO‐3 events analyses.

**Figure 4:**
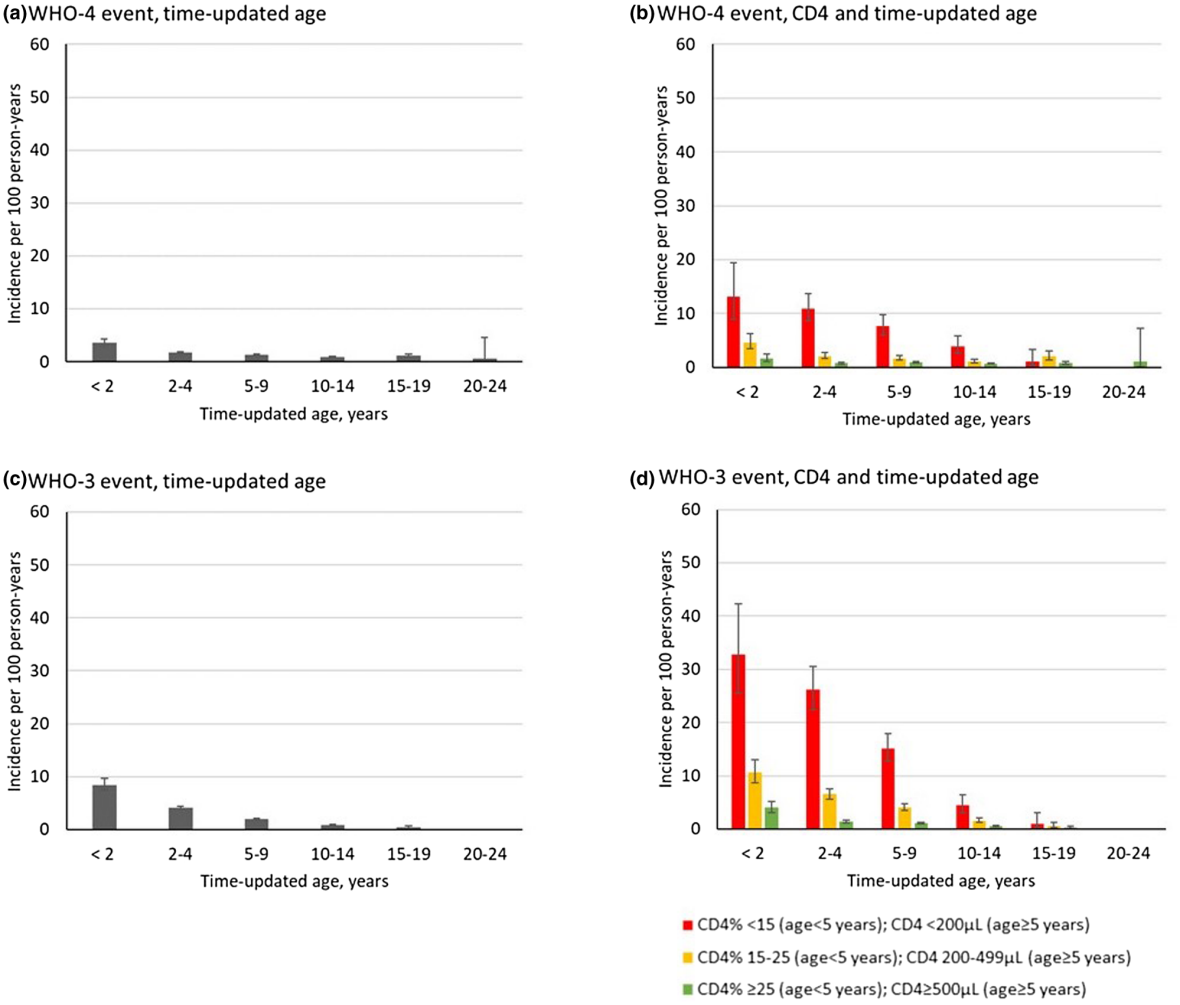
Incidence rate of post‐ART WHO‐4 events by (**a**) time‐updated age, and (**b**) CD4 and time‐updated age and WHO‐3 events by (**c**) time‐updated age, and (**d**) CD4 and time‐updated age, IeDEA multiregional cohort, 2004 to 2016. Error bars indicate 95% confidence intervals.

At the start of the post‐ART period, 18 children (<1%) had a prevalent WHO‐4 condition and were excluded from the analyses of incident post‐ART WHO‐4 events. Overall, 899 WHO‐4 events occurred among 16 061 children, during 72 412 PY of follow‐up. Recurrent severe bacterial infection and PCP represented >40% of these events (Table K, Figure B, Data S1). Post‐ART, the overall IR of first occurrence of a WHO‐4 event was 1.3/100 PY (95% CI: 1.2 to 1.4, Figure 4). Incidence was highest among <2‐year‐olds (3.5/100 PY, 95% CI: 2.9 to 4.4). IRs decreased in the older groups, remaining comparable between the older strata (Figure 4a, Tables A and G, Data S1). We also observed higher IRs in the lower CD4 strata (Figure 4b, Table B, Data S1). Regional data are available in Figure F and Table L, Data S1.

Twenty‐five children (<1%) had a prevalent WHO‐3 condition at the start of the post‐ART period and were excluded from the analyses of incident post‐ART WHO‐3 conditions. Among the remaining 16 054 children, 1410 (9%) experienced a first WHO‐3 event during 21 732 person‐years of follow‐up, yielding an overall IR of 2.1/100 PY (95% CI: 2.0 to 2.2). Oral candidiasis (31%) was the most frequently observed event (Figure B, Data S1). The IR of a first WHO‐3 event decreased as age increased, ranging from 8.4 (95% CI: 7.4 to 9.7) in <2‐year‐olds to 0.5/100 PY (95% CI: 0.3 to 0.7) in 15 to 19‐year‐olds, with no events recorded in 20 to 24‐year‐olds (Figure 4 and Table A, Data S1). We also found a significant difference between the IRs of first WHO‐3 events pre‐ and post‐ART in older youth >15 years by CD4 count strata: the highest IRs were observed in the lowest CD4 stratum (Figure 4 and Table B, Data S1). Regional data are available in Figure G and Table M of Data S1.

Additionally, 1125 TB events occurred in the post‐ART period; the estimated IR was 1.6/100 PY (95% CI: 1.5 to 1.7) (Table N of Data S1).

## 4 DISCUSSION

This large multiregional cohort study analyzed time‐updated age‐ and CD4‐adjusted incidence rates of mortality and first occurrence of WHO‐4 and WHO‐3 events (excluding TB) before and after ART initiation among 65 903 children, adolescents and youth living with PHIV over 259 912 PY of follow‐up. We make several key findings. First, post‐ART mortality rates were the highest at each end of the age spectrum relative to ages in the middle of the spectrum. Second, as age increased, participants in the lowest CD4 stratum had the highest mortality rates in the post‐ART period. Third, pre‐ART, IRs of both mortality and first occurrence of WHO‐4 and WHO‐3 events decreased as age and CD4 levels increased. Fourth, despite low incidence rates of clinical events, there remains a substantial burden of poor HIV‐related immune status and WHO‐4 and WHO‐3 events, both pre‐ and post‐ART.

We report an overall post‐ART mortality rate (1.4/100 PY) that is similar to those reported in other studies [16, 17, 18, 19, 20, 21, 22]. Younger children (<2 years) and those with low CD4 levels experienced higher rates of mortality, consistent with previous findings [16, 17, 18, 19, 20, 22, 23, 24]. We also found that mortality rates increased as participants reached ages 15 to 24. Indeed, these older children may have already experienced a fair amount of morbidity and resistance during their childhood, before entering HIV care, making them more vulnerable. In addition, this current generation of adolescents and youth who survived childhood with HIV, are challenged by a wide range of adolescent‐specific barriers to care, retention, and medication adherence exposing them to additional infectious morbidity and STIs [5, 25, 26]. This result underlines the need to identify and implement youth‐targeted interventions to ensure adolescents and youth living with PHIV are on effective regimens and engaged in care, and thus improve survival outcomes [25].

Notably, mortality incidence was higher at the lowest CD4 stratum during the post‐ART period compared to the pre‐ART period, for the same age strata. In this study were unable to link morbidity to mortality, however, given that the median age at ART initiation was 4.5 years, this likely reflects the late stage at which children, adolescents, and youth were accessing ART: children are initiating ART at very low CD4 counts, and mortality is occurring before immune reconstitution is possible [27, 28, 29]. At database closure, 4% of children in care had still not initiated ART in 2016. As universal ART is scaling‐up in accordance with 2017 WHO guidelines, there is an urgency to identify these children and initiate them on treatment as soon as possible [30].

Pre‐ART, the overall mortality rate was 2.8/100 PY. The <2‐year‐old mortality was close to 4‐fold higher (9.8/100 PY). However, these pre‐ART rates are lower than those observed in earlier studies [24, 31]. In our study, we report comparable rates only among those in the lowest CD4 stratum, ranging from 13 to 26/100 PY in sub‐Saharan African regions. Two key factors may have led us to underestimate mortality: first, median age at enrolment was 3.9 years. More than 50% of children born with HIV die by age 2 without ART, and thus many children may have died before accessing diagnosis and care, particularly in the earlier years [32]. Second, 16% of youth were classified as LTFU and were censored at the date of their last clinic visit while waiting to initiate ART, and some of those classified as LTFU may have actually died [33]. Indeed, in sensitivity analyses, we report high pre ART mortality among infants, reaching 36.1/100 PY (95% CI: 34.7 to 37.6) among those <2 years.

Few studies document the incidence of WHO‐4 and WHO‐3 events in children prior to initiating ART, and those that do were conducted in the pre‐ART era in high‐income countries. Overall, the pre‐ART IRs observed in our study are lower than those reported elsewhere [34]. Post‐ART, we make the same observation [23, 35]. We also noted marked differences between regions, where morbidity IRs were lower in sub‐Saharan Africa. This is most likely due to regional differences in the healthcare infrastructure and capacity to diagnose WHO‐4 and WHO‐3 events. In addition to having excluded TB events, the low pre‐ART opportunistic infection rates may be due to several factors, including the lower diagnostic capacity for opportunistic infections in resource‐limited settings and survivor bias (the sickest children either initiated ART or died before an event was recorded). Furthermore, we found that younger children were more at risk of disease progression and mortality. Those who survived to reach the older age groups in the pre‐ART period were likely children whose disease progression was slower. Of note, 13% of the 10 to 24‐year‐old age group had not yet initiated ART. Our results suggest that even in the setting of treat‐all policies, there remains a substantial burden of WHO‐4 and WHO‐3 events associated with poor HIV‐related immune status, in particular among younger children, and highlights the need to implement or re‐inforce strategies to reduce preventable infectious diseases in this population [36, 37].

Our study is affected by several limitations. First, survivor bias, as described above, likely led to an underestimation of mortality and clinical events IRs. Second, inclusion criteria included at least one CD4 measurement during both pre and post ART follow‐up, likely excluding those who died before access to CD4 was possible. Third, LTFU was high, particularly in the post‐ART period; this also may have led to unascertained morbidity and mortality: to account for this, and provide an interval in which true mortality IRs lie, we conducted a sensitivity analysis, under a maximum bias assumption where all LTFU was mortality. Fourth, excluding TB from the clinical events analysis underestimates WHO‐4 and WHO‐3 clinical events IRs. Indeed, in secondary analysis, we found high TB IRs. Although these findings are in line with previous studies, in the absence of reliably consistent application of TB case definitions, we feel our estimates are more robust as presented with this caveat [38, 39]. In addition, it is assumed that data on WHO‐4 and WHO‐3 events were incomplete and we were unable to link these events to mortality, both due to operational challenges in routine data collection as well as diagnostic limitations in our settings. We tried to address incompleteness by narrowing the study sample to a subset of our cohort in the clinical events analysis. Fifth, few data and follow‐up time are available in the 20 to 24 years age stratum, in particular in the pre‐ART period, making comparisons less meaningful. Despite this, as universal ART is scaled up, we feel this dataset provides a unique opportunity to describe this population of untreated 20 to 24‐year‐olds living with PHIV. Finally, we were unable to stratify results by sex, due to multiple stratifications leading to little person‐time per stratum.

Nonetheless, this study provides the largest report of both pre‐ and post‐ART, time‐updated age‐ and CD4‐stratified data on paediatric and adolescent mortality and WHO‐4 and WHO‐3 IRs (excluding TB) in the post‐ART era. The data presented are likely the most recent available describing children and adolescents living with HIV who are have not yet initiated ART and remain crucial to document the natural history of HIV progression. Furthermore, our results are strengthened by the study’s global scope and duration of follow‐up, providing estimates from infancy through age 24. While we likely underestimate IRs, but as CD4 testing becomes less available as countries shift to viral load monitoring, our data provide a valuable perspective on regional and global trends of IRs of morbidity and mortality by time‐updated age and CD4 level that will help to gauge current access to and quality of care. CD4 monitoring continues to play an important role in the clinical management of HIV, particularly for patients presenting late to care, and for treatment monitoring where viral load monitoring is restricted [40]. As Treat‐All implementation is scaling up in these regions, children living with PHIV will access ART at an earlier stage of their disease, likely impacting the morbidity and mortality IRs of the future generation of adolescents and youth living with PHIV.

## 5 CONCLUSIONS

Mortality and clinical event IRs were lower in our global cohort after ART initiation compared to before, except in younger children at low CD4 levels. Our data reflect the advanced stage of disease at which these children initiated ART across our cohorts and highlight the urgency in treating children at the earliest age possible. The high post‐ART mortality rates observed among children <2 years of age underscore the importance of prioritizing access to early HIV diagnosis and care in infants. Furthermore, while IRs decreased as age increased, this was no longer true in adolescents and young adults aged 15 to 24 years. Strengthening adolescent‐focused HIV services remains critical to improve long‐term outcomes among youth living with PHIV.

## COMPETING INTERESTS

Authors have no conflicts of interest.

## AUTHORS’ CONTRIBUTIONS

SDe, AMN, ALC, KWK, MAD and VL designed the study. KC, AE, KWK, SDu, MAD and VL provided data. KM, BM and GP extracted the data. SDe analysed the data. SDe and AMN drafted the manuscript. All authors have read, revised and approved the final version.

## Asia‐Pacific

The TREAT Asia Pediatric HIV Observational Database is an initiative of TREAT Asia, a program of amfAR, The Foundation for AIDS Research, with support from the US National Institutes of Health’s National Institute of Allergy and Infectious Diseases, the Eunice Kennedy Shriver National Institute of Child Health and Human Development, National Cancer Institute, National Institute of Mental Health, and National Institute on Drug Abuse as part of the International Epidemiology Databases to Evaluate AIDS (IeDEA; U01AI069907). The Kirby Institute is funded by the Australian Government Department of Health and Ageing, and is affiliated with the Faculty of Medicine, UNSW Australia. The content of this publication is solely the responsibility of the authors and does not necessarily represent the official views of any of the governments or institutions mentioned above.

Site investigators and cohorts: PS Ly, V Khol, National Centre for HIV/AIDS, Dermatology and STDs, Phnom Penh, Cambodia; J Tucker, New Hope for Cambodian Children, Phnom Penh, Cambodia; N Kumarasamy, E Chandrasekaran, Chennai Antiviral Research and Treatment Clinical Research Site (CART CRS), VHS‐Infectious Diseases Medical Centre, VHS, Chennai, India; A Kinikar, V Mave, S Nimkar, I Marbaniang, BJ Medical College and Sassoon General Hospitals, Maharashtra, India; DK Wati, D Vedaswari, IB Ramajaya, Sanglah Hospital, Udayana University, Bali, Indonesia; N Kurniati, D Muktiarti, Cipto Mangunkusumo – Faculty of Medicine Universitas Indonesia, Jakarta, Indonesia; SM Fong, M Lim, F Daut, Hospital Likas, Kota Kinabalu, Malaysia; NK Nik Yusoff, P Mohamad, Hospital Raja Perempuan Zainab II, Kelantan, Malaysia; TJ Mohamed, MR Drawis, Pediatric Institute, Hospital Kuala Lumpur, Kuala Lumpur, Malaysia; R Nallusamy, KC Chan, Penang Hospital, Penang, Malaysia; T Sudjaritruk, V Sirisanthana, L Aurpibul, Department of Pediatrics, Faculty of Medicine, and Research Institute for Health Sciences, Chiang Mai University, Chiang Mai, Thailand; R Hansudewechakul, P Ounchanum, S Denjanta, A Kongphonoi, Chiangrai Prachanukroh Hospital, Chiang Rai, Thailand; P Lumbiganon, P Kosalaraksa, P Tharnprisan, T Udomphanit, Division of Infectious Diseases, Department of Pediatrics, Faculty of Medicine, Khon Kaen University, Khon Kaen, Thailand; G Jourdain, PHPT‐IRD UMI 174 (Institut de recherche pour le développement and Chiang Mai University), Chiang Mai, Thailand; T Puthanakit, S Anugulruengkit, W Jantarabenjakul, R Nadsasarn, Department of Pediatrics, Faculty of Medicine and Research Unit in Pediatric and Infectious Diseases, Chulalongkorn University, Bangkok, Thailand; K Chokephaibulkit, K Lapphra, W Phongsamart, S Sricharoenchai, Department of Pediatrics, Faculty of Medicine Siriraj Hospital, Mahidol University, Bangkok, Thailand; KH Truong, QT Du, CH Nguyen, Children’s Hospital 1, Ho Chi Minh City, Vietnam; VC Do, TM Ha, VT An Children’s Hospital 2, Ho Chi Minh City, Vietnam; LV Nguyen, DTK Khu, AN Pham, LT Nguyen, National Hospital of Pediatrics, Hanoi, Vietnam; ON Le, Worldwide Orphans Foundation, Ho Chi Minh City, Vietnam; AH Sohn, JL Ross, T Suwanlerk, TREAT Asia/amfAR ‐ The Foundation for AIDS Research, Bangkok, Thailand; MG Law, A Kariminia, The Kirby Institute, UNSW Australia, Sydney, Australia.

## Caribbean, Central, and South America (CCASAnet)

This work was supported by the NIH‐funded Caribbean, Central and South America network for HIV epidemiology (CCASAnet), a member cohort of the International Epidemiologic Databases to Evaluate AIDS (leDEA) (U01AI069923). This award is funded by the following institutes: Eunice Kennedy Shriver National Institute Of Child Health & Human Development (NICHD), National Cancer Institute (NCI), National Institute Of Allergy And Infectious Diseases (NIAID), National Institute Of Mental Health (NIMH), the National Heart, Lung, and Blood Institute (NHLBI), the National Institute on Alcohol Abuse and Alcoholism (NIAAA), the National Institute of Diabetes and Digestive and Kidney Diseases (NIDDK), the Fogarty International Center (FIC), and the Office Of The Director, National Institutes Of Health (OD).

Fundación Huésped, Argentina: Pedro Cahn, Carina Cesar, Valeria Fink, Omar Sued, Emanuel Dell’Isola, Jose Valiente, Cleyton Yamamoto. Instituto Nacional de Infectologia‐Fiocruz, Brazil: Beatriz Grinsztejn, Valdilea Veloso, Paula Luz, Raquel de Boni, Sandra Cardoso Wagner, Ruth Friedman, Ronaldo Moreira. Universidade Federal de Minas Gerais, Brazil: Jorge Pinto, Flavia Ferreira, Marcelle Maia. Universidade Federal de São Paulo, Brazil: Regina Célia de Menezes Succi, Daisy Maria Machado, Aida de Fátima Barbosa Gouvêa. Fundación Arriarán, Chile: Marcelo Wolff, Claudia Cortes, Maria Fernanda Rodriguez, Gladys Allendes. Les Centres GHESKIO, Haiti: Jean William Pape, Vanessa Rouzier, Adias Marcelin, Christian Perodin. Hospital Escuela Universitario, Honduras: Marco Tulio Luque. Instituto Hondureño de Seguridad Social, Honduras: Denis Padgett. Instituto Nacional de Ciencias Médicas y Nutrición Salvador Zubirán, Mexico: Juan Sierra Madero, Brenda Crabtree Ramirez, Paco Belaunzaran, Yanink Caro Vega. Instituto de Medicina Tropical Alexander von Humboldt, Peru: Eduardo Gotuzzo, Fernando Mejia, Gabriela Carriquiry. Vanderbilt University Medical Center, USA: Catherine C McGowan, Bryan E Shepherd, Timothy Sterling, Karu Jayathilake, Anna K Person, Peter F Rebeiro, Jessica Castilho, Stephany N Duda, Fernanda Maruri, Hilary Vansell, Cathy Jenkins, Ahra Kim, Sarah Lotspeich.

## Central Africa

Research reported in this publication was supported by the National Institutes of Health’s National Institute of Allergy and Infectious Diseases (NIAID), the Eunice Kennedy Shriver National Institute of Child Health & Human Development (NICHD), the National Cancer Institute (NCI), the National Institute on Drug Abuse (NIDA), the National Heart, Lung, and Blood Institute (NHLBI), the National Institute on Alcohol Abuse and Alcoholism (NIAAA), the National Institute of Diabetes and Digestive and Kidney Diseases (NIDDK), the Fogarty International Center (FIC), the National Library of Medicine (NLM), and the Office of the Director (OD) under Award Number U01AI096299 (Central Africa‐IeDEA). The content is solely the responsibility of the authors and does not necessarily represent the official views of the National Institutes of Health.

Site investigators and cohorts: Nimbona Pélagie, Association Nationale de Soutien aux Séropositifs et Malade du Sida (ANSS), Burundi; Patrick Gateretse, Jeanine Munezero, Valentin Nitereka, Théodore Niyongabo, Christelle Twizere, Centre National de Reference en Matière de VIH/SIDA, Burundi; Hélène Bukuru, Thierry Nahimana, Centre Hospitalo‐Universitaire de Kamenge (CHUK), Burundi; Elysée Baransaka, Patrice Barasukana, Eugene Kabanda, Martin Manirakiza, François Ndikumwenayo, CHUK/Burundi National University, Burundi; Jérémie Biziragusenyuka, Ange Marie Michelline Munezero, Hospital Prince Régent Charles (HPRC), Burundi; Tabeyang Mbuh, Kinge Thompson Njie, Edmond Tchassem, Kien‐Atsu Tsi, Bamenda Hospital, Cameroon; Rogers Ajeh, Mark Benwi, Marc Lionel Ngamani, Victorine Nkome, Grace Toutou, Clinical Research Education and Consultancy (CRENC), Cameroon; Anastase Dzudie, CRENC and Douala General Hospital, Cameroon; Akindeh Mbuh, CRENC and University of Yaoundé, Cameroon; Djenabou Amadou, Amadou Dodo Balkissou, Eric Ngassam, Eric Walter Pefura Yone, Jamot Hospital, Cameroon; Alice Ndelle Ewanoge, Norbert Fuhngwa, Ernestine Kendowo, Chris Moki, Denis Nsame Nforniwe, Limbe Regional Hospital, Cameroon; Catherine Akele, Faustin Kitetele, Patricia Lelo, Martine Tabala, Kalembelembe Pediatric Hospital, Democratic Republic of Congo; Emile Wemakoy Okitolonda, Cherubin Ekembe, Kinshasa School of Public Health, Democratic Republic of Congo; Merlin Diafouka, Martin Herbas Ekat, Dominique Mahambou Nsonde, CTA Brazzaville, Republic of Congo; Adolphe Mafou, CTA Pointe‐Noire, Republic of Congo; Nicole Ayinkamiye, Jules Igirimbabazi, Bethsaida Health Center, Rwanda; Emmanuel Ndamijimana, Providance Uwineza, Busanza Health Center, Rwanda; Emmanuel Habarurema, Marie Luise Nyiraneza, Gahanga Health Center, Rwanda; Dorothee Mukamusana, Liliane Tuyisenge, Gikondo Health Center, Rwanda; Catherine Kankindi, Christian Shyaka, Kabuga Health Center, Rwanda; Marie Grace Ingabire, Bonheur Uwakijijwe, Kicukiro Health Center, Rwanda; Jules Ndumuhire, Marie Goretti Nyirabahutu, Masaka Health Center, Rwanda; Yvette Ndoli, Oliver Uwamahoro, Nyarugunga Health Center, Rwanda; Ribakare Muhayimpundu, Sabin Nsanzimana, Eric Remera, Esperance Umurarungu, Rwanda Biomedical Center, Rwanda; Lydia Busingye, Alex M Butera, Josephine Gasana, Thierry Habiryayo, Charles Ingabire, Jules Kabahizi, Jean Chrysostome Kagimbana, Faustin Kanyabwisha, Gallican Kubwimana, Benjamin Muhoza, Athanase Munyaneza, Gad Murenzi, Francoise Musabyimana, Francine Mwiza, Gallican Nshogoza Rwibasira, Jean d'Amour Sinayobye, Patrick Tuyisenge, Rwanda Military Hospital, Rwanda; Chantal Benekigeri, Jacqueline Musaninyange, WE‐ACTx Health Center, Rwanda.

Coordinating and Data Centers: Adebola Adedimeji, Kathryn Anastos, Madeline Dilorenzo, Lynn Murchison, Jonathan Ross, Marcel Yotebieng, Albert Einstein College of Medicine, USA; Diane Addison, Ellen Brazier, Heidi Jones, Elizabeth Kelvin, Sarah Kulkarni, Denis Nash, Matthew Romo, Olga Tymejczyk, Institute for Implementation Science in Population Health, Graduate School of Public Health and Health Policy, City University of New York (CUNY), USA; Batya Elul, Columbia University, USA; Xiatao Cai, Allan Dong, Don Hoover, Hae‐Young Kim, Chunshan Li, Qiuhu Shi, Data Solutions, USA; Robert Agler, Kathryn Lancaster, The Ohio State University, USA; Mark Kuniholm, University at Albany, State University of New York, USA; Andrew Edmonds, Angela Parcesepe, Jess Edwards, University of North Carolina at Chapel Hill, USA; Olivia Keiser, University of Geneva; Stephany Duda; Vanderbilt University School of Medicine, USA; April Kimmel, Virginia Commonwealth University School of Medicine, USA.

## East Africa IeDEA

Research reported in this publication was supported by the National Institute Of Allergy And Infectious Diseases (NIAID), Eunice Kennedy Shriver National Institute Of Child Health & Human Development (NICHD), National Institute On Drug Abuse (NIDA), National Cancer Institute (NCI), the National Institute of Mental Health (NIMH), the National Heart, Lung, and Blood Institute (NHLBI), the National Institute on Alcohol Abuse and Alcoholism (NIAAA), the National Institute of Diabetes and Digestive and Kidney Diseases (NIDDK), and the Fogarty International Center (FIC), in accordance with the regulatory requirements of the National Institutes of Health under Award Number U01AI069911 East Africa IeDEA Consortium. The content is solely the responsibility of the authors and does not necessarily represent the official views of the National Institutes of Health.

Site investigators and cohorts (with data managers): Diero L, Sang E, MOI University, AMPATH Plus, Eldoret, Kenya; Bukusi E, Elisheba Mutegi, KEMRI (Kenya Medical Research Institute), Kisumu, Kenya; Charles Kasozi, Lydia Buzaalirwa, Mathew Ssemakadde, Masaka Regional Referral Hospital, Masaka, Uganda; Mwebesa Bosco Bwana, Michael Kanyesigye, Mbarara University of Science and Technology (MUST), Mbarara, Uganda; Barbara Castelnuovo; John Michael Matovu, Infectious Diseases Institute (IDI), Mulago, Uganda; Fred Nalugoda, Francis X Wasswa, Rakai Health Sciences Program, Kalisizo, Uganda, Paul Kazyoba, Mary Mayige, (National Institute for Medical Research (NIMR), Dar es Salaam Tanzania), and Rita Elias Lyamuya, Francis Mayanga, Morogoro Regional Hospital, Morogoro, Tanzania; Kapella Ngonyani, Jerome Lwali, Tumbi Regional Hospital, Pwani, Tanzania; Mark Urassa and Charles Nyaga (National Institute for Medical Research (NIMR‐Kisesa), Mwanza, Tanzania), Tanzania; Rachel Vreeman (Mt. Sinai, New York, USA) Kara Wools‐Kaloustian, Constantin Yiannoutsos, Beverly Musick, Indiana University School of Medicine, Indiana University, Indianapolis, IN, USA; Batya Elul, Columbia University, New York City, NY, USA; Jennifer Syvertsen, University of California Riverside, California, USA; Rami Kantor, Brown University/Miriam Hospital, Providence, RI, USA; Jeffrey Martin, Megan Wenger, Craig Cohen, Jayne Kulzer, University of California, San Francisco, CA, USA; Paula Braitstein, University of Toronto, Toronto, Canada.

## IeDEA Southern Africa

Research reported in this publication was supported by the Eunice Kennedy Shriver National Institute Of Child Health & Human Development (NICHD), National Institute Of Diabetes And Digestive And Kidney Diseases (NIDDK), National Institute On Drug Abuse (NIDA), National Heart, Lung, And Blood Institute (NHLBI), National Institute On Alcohol Abuse And Alcoholism (NIAAA), Fogarty International Center (FIC), National Cancer Institute (NCI), and by the National Institute of Allergy and Infectious Diseases of the National Institutes of Health under Award Number U01AI069924. The content is solely the responsibility of the authors and does not necessarily represent the official views of the National Institutes of Health.

Site investigators and cohorts: Gary Maartens, Aid for AIDS, South Africa; Carolyn Bolton, Michael Vinikoor, Centre for Infectious Disease Research in Zambia (CIDRZ), Zambia; Robin Wood and Catherine Orrell, Gugulethu ART Programme, South Africa; Nosisa Sipambo, Harriet Shezi Clinic, South Africa; Frank Tanser, Africa Centre for Health & Population Studies (Hlabisa), South Africa; Andrew Boulle, Khayelitsha ART Programme, South Africa; Geoffrey Fatti, Kheth’Impilo, South Africa; Sam Phiri, Lighthouse Clinic, Malawi; Cleophas Chimbetete, Newlands Clinic, Zimbabwe; Karl Technau, Rahima Moosa Mother and Child Hospital, South Africa; Brian Eley, Red Cross Children's Hospital, South Africa; Josephine Muhairwe, SolidarMed Lesotho; Juan Burgos‐Soto, SolidarMed Mozambique; Cordelia Kunzekwenyika, SolidarMed Zimbabwe, Matthew P Fox, Themba Lethu Clinic, South Africa; Hans Prozesky, Tygerberg Academic Hospital, South Africa.

Data centers: Nina Anderegg, Marie Ballif, Lina Bartels, Julia Bohlius, Benedikt Christ, Felix Cuneo, Cam Ha Dao Ostinelli, Masa Davidovic, Tafadzwa Dhokotera, Matthias Egger, Lukas Fenner, Per von Groote, Andreas Haas, Taghavi Katayoun, Serra Lem, Martina Reichmuth, Veronika Skrivankova, Lilian Smith, Gilles Wandeler, Elizabeth Zaniewski, Kathrin Zürcher, Institute of Social and Preventive Medicine, University of Bern, Switzerland; Kim Anderson, Andrew Boulle, Morna Cornell, Mary‐Ann Davies, Victoria Iyun, Leigh Johnson, Reshma Kassanjee, Kathleen Kehoe,Mmamapudi Kubjane, Nicola Maxwell, Patience Nyakato, Ernest Mokotoane, Gem Patten, Mpho Tlali, Priscilla Tsondai, Renee de Waal, School of Public Health and Family Medicine, University of Cape Town, South Africa.

## West Africa

Research reported in this publication was supported by the US National Institutes of Health (NIAID, NICHD, NCI, NIMH, NHLBI, NIAAA, NIDDK, FIC) under Award Number U01AI069919 (PI: Dabis). The content is solely the responsibility of the authors and does not necessarily represent the official views of the National Institutes of Health.”

## Site investigators and cohorts

Adult cohorts: Marcel Djimon Zannou, CNHU, Cotonou, Benin; Armel Poda, CHU Souro Sanou, Bobo Dioulasso, Burkina Faso; Fred Stephen Sarfo & Komfo Anokeye Teaching Hospital, Kumasi, Ghana; Eugene Messou, ACONDA CePReF, Abidjan, Cote d’Ivoire; Henri Chenal, CIRBA, Abidjan, Cote d’Ivoire; Kla Albert Minga, CNTS, Abidjan, Cote d’Ivoire; Emmanuel Bissagnene, & Aristophane Tanon, CHU Treichville, Cote d’Ivoire; Moussa Seydi, CHU de Fann, Dakar, Senegal; Akessiwe Akouda Patassi, CHU Sylvanus Olympio, Lomé, Togo.

Pediatric cohorts: Sikiratou Adouni Koumakpai‐Adeothy,_CNHU, Cotonou, Benin; Lorna Awo Renner, Korle Bu Hospital, Accra, Ghana; Sylvie Marie N’Gbeche, ACONDA CePReF, Abidjan, Ivory Coast; Clarisse Amani Bosse, ACONDA_MTCT+, Abidjan, Ivory Coast; Kouadio Kouakou, CIRBA, Abidjan, Cote d’Ivoire; Madeleine Amorissani Folquet, CHU de Cocody, Abidjan, Cote d’Ivoire; François Tanoh Eboua, CHU de Yopougon, Abidjan, Cote d’Ivoire; Fatoumata Dicko Traore, Hopital Gabriel Toure, Bamako, Mali; Elom Takassi, CHU Sylvanus Olympio, Lomé,Togo.

Coordinating & data centers: François Dabis, Elise Arrive, Eric Balestre, Renaud Becquet, Charlotte Bernard, Shino Chassagne Arikawa, Alexandra Doring, Antoine Jaquet, Karen Malateste, Elodie Rabourdin, Thierry Tiendrebeogo, ADERA, Isped & INSERM U1219, Bordeaux, France. Sophie Desmonde, Julie Jesson, Valeriane Leroy, Inserm 1027, Toulouse, France Didier Koumavi Ekouevi, Jean‐Claude Azani, Patrick Coffie, Abdoulaye Cissé, Guy Gnepa, Apollinaire Horo, Christian Kouadio, Boris Tchounga, PACCI, CHU Treichville, Abidjan, Côte d’Ivoire.

### FUNDING

Research reported in this publication was supported by the US National Institutes of Health. Asia‐Pacific: The TREAT Asia Pediatric HIV Observational Database is an initiative of TREAT Asia, a programme of amfAR, The Foundation for AIDS Research, with support from the US National Institutes of Health’s (NIH) National Institute of Allergy and Infectious Diseases (NIAID), the Eunice Kennedy Shriver National Institute of Child Health and Human Development (NICHD), National Cancer Institute (NCI), National Institute of Mental Health (NIMH), National Institute on Drug Abuse (NIDA), the National Heart, Lung, and Blood Institute (NHLBI), the National Institute on Alcohol Abuse and Alcoholism (NIAAA), the National Institute of Diabetes and Digestive and Kidney Diseases (NIDDK), and the Fogarty International Center (FIC) as part of the International Epidemiology Databases to Evaluate AIDS (IeDEA; U01AI069907). The Caribbean, Central and South America network for HIV epidemiology (CCASAnet; U01AI069923) is supported by NICHD, NCI, NIAID, NIMH, NHLBI, NIAAA, NIDDK, FIC, and the Office of the Director, National Institutes of Health (OD).” Central Africa research reported in this publication was supported by NIAID, NICHD, NCI, NIDA, NHLBI, NIAAA, NIDDK, FIC, the National Library of Medicine (NLM), and OD under Award Number U01AI096299 (Central Africa‐IeDEA). East African Research reported in this publication was supported by NIAID, NICHD, NIDA, NCI, and NIMH, in accordance with the regulatory requirements of the NIH under Award Number U01AI069911 (KWK is a PI). Southern African research reported in this publication was supported by the NIAID of the NIH under Award Number U01AI069924 (MAD is a PI). West African Research reported in this publication is supported by NIAID, NICHD, NCI and NIMH under award number U01AI069919. Additional support for this research included K08 HD094638 to AMN and R01HD079214 to ALC. The funders had no role in study design, data collection and analysis, decision to publish, or preparation of the manuscript. The content is solely the responsibility of the authors and does not necessarily represent the official views of the above institutions.

## Supporting information


**Data S1.** Supplemental digital content 1.
**Data S2.** This file provides the complete IeDEA global funding acknowledgments for each region.

## References

[1] Azcoaga‐Lorenzo A , Ferreyra C , Alvarez A , Palma P , Velilla E , Del Amo J . Effectiveness of a PMTCT programme in rural Western Kenya. AIDS Care. 2011;23(3):274–80. 21347890 10.1080/09540121.2010.507750

[2] Anaky MF , Duvignac J , Wemin L , Kouakoussui A , Karcher S , Touré S , et al. Scaling up antiretroviral therapy for HIV‐infected children in Côte d'Ivoire: determinants of survival and loss to programme. Bull World Health Organ. 2010;88(7):490–9. 20616968 10.2471/BLT.09.068015 PMC2897983

[3] World Health Organisation . UNAIDS Data 2018. 2018 Available from: http://www.unaids.org/sites/default/files/media_asset/unaids‐data‐2018_en.pdf

[4] Johnson LF , Davies MA , Moultrie H , Sherman GG , Bland RM , Rehle TM , et al. The effect of early initiation of antiretroviral treatment in infants on pediatric AIDS mortality in South Africa: a model‐based analysis. Pediatr Infect Dis J. 2012;31(5):474–80. 22189531 10.1097/INF.0b013e3182456ba2

[5] Mofenson LM , Cotton MF . The challenges of success: adolescents with perinatal HIV infection. J Int AIDS Soc. 2013;16:18650. 23782484 10.7448/IAS.16.1.18650 PMC3687076

[6] Wong VJ , Murray KR , Phelps BR , Vermund SH , McCarraher DR . Adolescents, young people, and the 90–90‐90 goals: a call to improve HIV testing and linkage to treatment. AIDS. 2017;31(Suppl 3):S191–4. 28665876 10.1097/QAD.0000000000001539 PMC5497776

[7] Sohn AH , Hazra R . The changing epidemiology of the global paediatric HIV epidemic: keeping track of perinatally HIV‐infected adolescents. J Int AIDS Soc. 2013;16:18555. 23782474 10.7448/IAS.16.1.18555 PMC3687075

[8] Slogrove AL , Sohn AH . The global epidemiology of adolescents living with HIV: time for more granular data to improve adolescent health outcomes. Curr Opin HIV AIDS. 2018;13(3):170–8. 29432227 10.1097/COH.0000000000000449 PMC5929160

[9] IeDEA Pediatric Working Group . A survey of paediatric HIV programmatic and clinical management practices in Asia and sub‐Saharan Africa—the International epidemiologic Databases to Evaluate AIDS (IeDEA). J Int AIDS Soc. 2013;16(1):17998. 23336728 10.7448/IAS.16.1.17998 PMC3547123

[10] World Health Organisation . WHO case definitions of HIV for surveillance and revised clinical staging and immunological classification of HIV‐related disease in adults and children. 2007 Available from: https://apps.who.int/iris/bitstream/handle/10665/43699/9789241595629_eng.pdf?sequence=1&isAllowed=y

[11] Reid MJ , Saito S , Fayorsey R , Carter RJ , Abrams EJ . Assessing capacity for diagnosing tuberculosis in children in sub‐Saharan African HIV care settings. Int J Tuberc Lung Dis. 2012;16(7):924–7. 22583761 10.5588/ijtld.11.0816 PMC5920677

[12] Marais BJ , Gie RP , Hesseling AC , Schaaf HS , Lombard C , Enarson DA , et al. A refined symptom‐based approach to diagnose pulmonary tuberculosis in children. Pediatrics. 2006;118(5):e1350–9. 10.1542/peds.2006-0519 17079536

[13] Bolton‐Moore C , Mubiana‐Mbewe M , Cantrell RA , Chintu N , Stringer EM , Chi BH , et al. Clinical outcomes and CD4 cell response in children receiving antiretroviral therapy at primary health care facilities in Zambia. JAMA. 2007;298(16):1888–99. 17954540 10.1001/jama.298.16.1888

[14] Patel K , Hernan MA , Williams PL , Seeger JD , McIntosh K , Dyke RB , et al. Long‐term effects of highly active antiretroviral therapy on CD4+ cell evolution among children and adolescents infected with HIV: 5 years and counting. Clin Infect Dis. 2008;46(11):1751–60. 18426371 10.1086/587900 PMC3154876

[15] Desmonde S , Dicko F , Koueta F , Eboua T , Balestre E , Amani‐Bosse C , et al. Association between age at antiretroviral therapy initiation and 24‐month immune response in HIV‐infected children in West Africa. AIDS. 2014;1645–55. 24804858 10.1097/QAD.0000000000000272 PMC4169343

[16] Sutcliffe CG , van Dijk JH , Bolton C , Persaud D , Moss WJ . Effectiveness of antiretroviral therapy among HIV‐infected children in sub‐Saharan Africa. Lancet Infect Dis. 2008;8(8):477–89. 18652994 10.1016/S1473-3099(08)70180-4

[17] Rouet F , Fassinou P , Inwoley A , Anaky MF , Kouakoussui A , Rouzioux C , et al. Long‐term survival and immuno‐virological response of African HIV‐1‐infected children to highly active antiretroviral therapy regimens. AIDS. 2006;20(18):2315–9. 17117017 10.1097/QAD.0b013e328010943b

[18] Nyandiko WM , Ayaya S , Nabakwe E , Tenge C , Sidle JE , Yiannoutsos CT , et al. Outcomes of HIV‐infected orphaned and non‐orphaned children on antiretroviral therapy in western Kenya. J Acquir Immune Defic Syndr. 2006;43(4):418–25. 17099313 10.1097/01.qai.0000243122.52282.89

[19] Reddi A , Leeper SC , Grobler AC , Geddes R , France KH , Dorse GL , et al. Preliminary outcomes of a paediatric highly active antiretroviral therapy cohort from KwaZulu‐Natal, South Africa. BMC Pediatr. 2007;7:13. 17367540 10.1186/1471-2431-7-13 PMC1847430

[20] Walker AS , Mulenga V , Ford D , Kabamba D , Sinyinza F , Kankasa C , et al. The impact of daily cotrimoxazole prophylaxis and antiretroviral therapy on mortality and hospital admissions in HIV‐infected Zambian children. Clin Infect Dis. 2007;44(10):1361–7. 17443476 10.1086/515396

[21] Njom Nlend AE , Loussikila AB . Predictors of mortality among HIV‐infected children receiving highly active antiretroviral therapy. Med Mal Infect. 2017;47(1):32–7. 27609595 10.1016/j.medmal.2016.07.003

[22] Phongsamart W , Hansudewechakul R , Bunupuradah T , Klinbuayaem V , Teeraananchai S , Prasithsirikul W , et al. Long‐term outcomes of HIV‐infected children in Thailand: the Thailand Pediatric HIV Observational Database. Int J Infect Dis. 2014;22:19–24. 24576843 10.1016/j.ijid.2013.12.011

[23] Desmonde S , Essanin JB , Aka AE , Messou E , Amorissani‐Folquet M , Rondeau V , et al. Morbidity and health care resource utilization in HIV‐infected children after antiretroviral therapy initiation in Cote d'Ivoire, 2004–2009. J Acquir Immune Defic Syndr. 2014;65(3):e95–103. 10.1097/QAI.0b013e3182a4ea6f PMC3982324 24525473

[24] Walker AS , Mulenga V , Sinyinza F , Lishimpi K , Nunn A , Chintu C , et al. Determinants of survival without antiretroviral therapy after infancy in HIV‐1‐infected Zambian children in the CHAP Trial. J Acquir Immune Defic Syndr. 2006;42(5):637–45. 16868501 10.1097/01.qai.0000226334.34717.dc

[25] Collaborative Initiative for Paediatric HIVE, Research Global Cohort C , Slogrove AL , Schomaker M , Davies MA , Williams P , Balkan S , et al. The epidemiology of adolescents living with perinatally acquired HIV: A cross‐region global cohort analysis. PLoS Med. 2018;15(3):e1002514. 29494593 10.1371/journal.pmed.1002514 PMC5832192

[26] Adejumo OA , Malee KM , Ryscavage P , Hunter SJ , Taiwo BO . Contemporary issues on the epidemiology and antiretroviral adherence of HIV‐infected adolescents in sub‐Saharan Africa: a narrative review. J Int AIDS Soc. 2015;18:20049. 26385853 10.7448/IAS.18.1.20049 PMC4575412

[27] Ekouevi DK , Azondekon A , Dicko F , Malateste K , Touré P , Eboua FT , et al. 12‐month mortality and loss‐to‐program in antiretroviral‐treated children: the IeDEA pediatric West African Database to evaluate AIDS (pWADA), 2000–2008. BMC Public Health. 2011;11(1):2000–8. 10.1186/1471-2458-11-519 PMC3142512 21718505

[28] Kids ARTLC . Low risk of death, but substantial program attrition, in pediatric HIV treatment cohorts in Sub‐Saharan Africa. J Acquir Immune Defic Syndr. 2008;49(5):523–31. 18989227 10.1097/QAI.0b013e31818aadce

[29] Kiboneka A , Wangisi J , Nabiryo C , Tembe J , Kusemererwa S , Olupot‐Olupot P , et al. Clinical and immunological outcomes of a national paediatric cohort receiving combination antiretroviral therapy in Uganda. AIDS. 2008;22(18):2493–9. 19005272 10.1097/QAD.0b013e328318f148

[30] Barlow‐Mosha L , Musiime V , Davies MA , Prendergast AJ , Musoke P , Siberry G , et al. Universal antiretroviral therapy for HIV‐infected children: a review of the benefits and risks to consider during implementation. J Int AIDS Soc. 2017;20(1):21552. 28691434 10.7448/IAS.20.1.21552 PMC5527851

[31] Becquet R , Marston M , Dabis F , Moulton LH , Gray G , Coovadia HM , et al. Children who acquire HIV infection perinatally are at higher risk of early death than those acquiring infection through breastmilk: a meta‐analysis. PLoS One. 2012;7(2):e28510. 22383946 10.1371/journal.pone.0028510 PMC3285615

[32] Newell M‐L , Coovadia H , Cortina‐Borja M , Rollins N , Gaillard P , Dabis F . Mortality of infected and uninfected infants born to HIV‐infected mothers in Africa: a pooled analysis. Lancet. 2004;364(9441):1236–43. 15464184 10.1016/S0140-6736(04)17140-7

[33] Braitstein P , Songok J , Vreeman RC , Wools‐Kaloustian KK , Koskei P , Walusuna L , et al. "Wamepotea" (they have become lost): outcomes of HIV‐positive and HIV‐exposed children lost to follow‐up from a large HIV treatment program in western Kenya. J Acquir Immune Defic Syndr. 2011;57(3):e40–6. 10.1097/QAI.0b013e3182167f0d PMC3145828 21407085

[34] Nesheim SR , Hardnett F , Wheeling JT , Siberry GK , Paul ME , Emmanuel P , et al. Incidence of opportunistic illness before and after initiation of highly active antiretroviral therapy in children. Pediatr Infect Dis J. 2013;32(10):1089–95. 24067552 10.1097/INF.0b013e31829ee893 PMC3785006

[35] Mubiana‐Mbewe M , Bolton‐Moore C , Banda Y , Chintu N , Nalubamba‐Phiri M , Giganti M , et al. Causes of morbidity among HIV‐infected children on antiretroviral therapy in primary care facilities in Lusaka, Zambia. Trop Med Int Health. 2009;14(10):1190–8. 19708902 10.1111/j.1365-3156.2009.02360.x PMC3897250

[36] McHugh G , Rylance J , Mujuru H , Nathoo K , Chonzi P , Dauya E , et al. Chronic morbidity among older children and adolescents at diagnosis of HIV infection. J Acquir Immune Defic Syndr. 2016;73(3):275–81. 27171738 10.1097/QAI.0000000000001073 PMC5065928

[37] Adetokunboh OO , Awotiwon A , Ndwandwe D , Uthman OA , Wiysonge CS . The burden of vaccine‐preventable diseases among HIV‐infected and HIV‐exposed children in sub‐Saharan Africa: a systematic review and meta‐analysis. Human Vacc Immunother. 2019;15(11):2590–605. 10.1080/21645515.2019.1599676 PMC6930054 30945963

[38] Nelson LJ , Wells CD . Global epidemiology of childhood tuberculosis. Int J Tuberc Lung Dis. 2004;8(5):636–47. 15137548

[39] Wiseman CA , Schaaf HS , Cotton MF , Gie RP , Jennings T , Whitelaw A , et al. Bacteriologically confirmed tuberculosis in HIV‐infected infants: disease spectrum and survival. Int J Tuberc Lung Dis. 2011;15(6):770–5. 21575297 10.5588/ijtld.10.0501

[40] Ford N , Meintjes G , Pozniak A , Bygrave H , Hill A , Peter T , et al. The future role of CD4 cell count for monitoring antiretroviral therapy. Lancet Infect Dis. 2015;15(2):241–7. 25467647 10.1016/S1473-3099(14)70896-5

