# Supplementary material for: Time‐varying age‐ and CD4‐stratified rates of mortality and WHO stage 3 and stage 4 events in children, adolescents and youth 0 to 24 years living with perinatally acquired HIV, before and after antiretroviral therapy initiation in the paediatric IeDEA Global Cohort Consortium
Source: J Int AIDS Soc. 2020 Oct 9;23(10):e25617. doi: 10.1002/jia2.25617 (PMC7545918; doi:10.1002/jia2.25617)
Supplement: Supplementary file 1 — Data S1. Supplemental digital content 1. Data S2. This file provides the complete IeDEA global funding acknowledgments for each region. [file JIA2-23-e25617-s001.docx]

**Time-varying age- and CD4-stratified rates of mortality and WHO stage 3 and stage 4 events in children, adolescents, and youth 0-24 years living with perinatally acquired HIV, before and after antiretroviral therapy initiation in the paediatric IeDEA Global Cohort Consortium**

**Supplemental Digital Content 1**

Sophie Desmonde, PhD, *et al.*

This Appendix provides methodological detail and results in addition to those presented in the main manuscript.

**METHODS**

**Outcomes and key definitions**

We analysed the first event that occurred (excluding TB) during follow-up within a given event class for a given patient. Diagnoses were analysed as they were recorded in regional databases, based on methods available in routine clinical care at each site, including clinical, radiographic, laboratory, and pathologic evaluations. There was no assessment by an independent validation committee. Observed diagnoses were classified into the event classes below.

**WHO Stage 4 event**: disseminated bacillus Calmette-Guerin (BCG) disease, candidiasis (trachea, bronchi or lungs), candidiasis oesophogeal, cervical cancer (invasive); cryptococcosis extrapulmonary; cryptosporidiosis (duration >1 month); cytomegalovirus other location (site other than liver, spleen or lymph nodes; onset at age >1 month); herpes simplex virus ulcers (duration >1 month); histoplasmosis extrapulmonary; HIV encephalopathy, HIV wasting syndrome, Isosporiasis diarrhoea (duration >1 month); Kaposi sarcoma; leishmaniasis visceral; Mycobacterium avium complex (MAC) or kansasii extrapulmonary; mycobacterium extrapulmonary (other, excluding BCG in children); non-Hodgkin Lymphoma, diffuse large B-cell lymphoma (immunoblastic or centroblastic); Pneumocystis carinii pneumonia (PCP); progressive multifocal leukoencephalopathy; recurrent severe presumed bacterial infection excluding pneumonia (if aged ≥15 years); Salmonella bacteraemia (non-typhoid) recurrent; Toxoplasmosis brain (outside neonatal period); visceral herpes simplex infection; unexplained severe malnutrition or wasting; HIV-associated nephropathy, and HIV-associated cardiomyopathy

**WHO Stage 3 event**: unexplained anaemia (<8g/dl), and/or neutropaenia (<1,000/mm^3^), and thrombocytopaenia (<50,000/mm^3^) (duration >1 month); recurrent severe presumed bacterial infection excluding pneumonia (if aged <15 years); oral candidiasis (outside of neonatal period); unexplained chronic diarrhoea (>14 days if <15 years; >1 month if ≥15 years); unexplained persistent fever (>1 month); unexplained moderate malnutrition or wasting; symptomatic lymphoid interstitial pneumonitis; acute necrotizing ulcerative stomatitis, gingivitis or periodontitis; oral hairy leukoplakia; severe unexplained weight loss (>10% of body weight); chronic HIV-associated lung disease

**RESULTS**

Key results are presented in the main manuscript. In addition, Supplemental Tables A-H report incidence rates for mortality, earliest WHO-4 event, and earliest WHO-3 event, stratified by time-updated age and time-updated CD4. These results are graphically depicted in manuscript Figures 1-4.

**Table A. Event rates of mortality, occurrence of first WHO-4 event, and occurrence of first WHO-3 event during follow-up, by time-updated age**

| **Outcome of interest** |  | **Age, years** | | | | | | ***p-value^¥^*** |
| --- | --- | --- | --- | --- | --- | --- | --- | --- |
|  |  | **0-2** | **2-4** | **5-9** | **10-14** | **15-19** | **20-24** |  |
| ***Pre-ART**** |  |  |  |  |  |  |  |  |
| Mortality | IR^†^ | 9.8 | 3.4 | 1.1 | 0.6 | 0.9 | 0.0 | *<0.001* |
|  | 95%CI^§^ | [9.1-10.6] | [3.1-3.8] | [1.0-1.2] | [0.5-0.8] | [0.3-2.8] | - |  |
| Mortality (counting LTFU) | IR^†^ | 36.1 | 21.7 | 15.5 | 9.0 | 13.0 | 0.0 | *<0.001* |
|  | 95%CI^§^ | [34.7-37.6] | [20.9-22.5] | [15.0-16.0] | [8.3-9.8] | [9.7-17.6] | - |  |
| WHO-4 event | IR^†^ | 7.4 | 3.2 | 2.5 | 1.3 | 0.0 | 0.0 | *<0.001* |
|  | 95%CI^§^ | [6.4-8.5] | [2.7-3.7] | [2.3-2.8] | [1.0-1.8] | - | - |  |
| WHO-3 event | IR^†^ | 20.5 | 9.4 | 4.6 | 1.8 | 0.7 | N/A | *<0.001* |
|  | 95%CI^§^ | [18.8-22.5] | [8.6-10.3] | [4.2-5.0] | [1.4-2.3] | [0.1-5.0] |  |  |
| ***Post-ART**** |  |  |  |  |  |  |  |  |
| Mortality | IR^†^ | 8.6 | 1.8 | 1.0 | 0.7 | 0.8 | 2.3 | *<0.001* |
|  | 95%CI^§^ | [8.1-9.2] | [1.7-2.0] | [0.9-1.0] | [0.6-0.7] | [0.7-1.0] | [1.1-4.9] |  |
| Mortality (counting LTFU) | IR^†^ | 22.8 | 17.8 | 8.8 | 10.6 | 15.8 | 26.3 | *<0.001* |
|  | 95%CI^§^ | [21.9-23.8] | [17.4-18.2] | [8.6-9.0] | [10.3-10.9] | [15.1-16.6] | [21.1-32.8] |  |
| WHO-4 event | IR^†^ | 3.5 | 1.7 | 1.2 | 0.9 | 1.1 | 0.7 | *<0.001* |
|  | 95%CI^§^ | [2.9-4.4] | [1.5-2.0] | [1.1-1.4] | [0.8-1.0] | [0.8-1.5] | [0.1-4.6] |  |
| WHO-3 event | IR^†^ | 8.4 | 4.0 | 1.9 | 0.9 | 0.5 | 0.0 | *<0.001* |
|  | 95%CI^§^ | [7.4-9.7] | [3.7-4.4] | [1.8-2.1] | [0.8-1.0] | [0.3-0.7] | - |  |

* ART: antiretroviral therapy; ^†^ IR: incidence rate; ^§^ 95%CI: 95% confidence interval; ^¥^ *p*-value was calculated in a Poisson model adjusted for age

**Table B. Event rates of mortality, occurrence of first WHO-4 event, and occurrence of first WHO-3 event during follow-up, by time-updated CD4**

| **Outcome of interest** |  | **<5 years; CD4%** | | | ***p-value^¥^*** | **≥5 years; CD4 cells/µL** | | | ***p-value^¥^*** |
| --- | --- | --- | --- | --- | --- | --- | --- | --- | --- |
|  |  | **< 15%** | **15-24%** | **≥25%** |  | **<200** | **200-499** | **≥500** |  |
| ***Pre-ART**** |  |  |  |  |  |  |  |  |  |
| Mortality | IR^†^ | 14.0 | 4.8 | 3.3 | *<0.001* | 5.9 | 1.0 | 0.5 | *<0.001* |
|  | 95%CI^§^ | [12.7-15.4] | [4.4-5.3] | [2.9-3.8] |  | [5.0-7.1] | [0.9-1.3] | [0.4-0.6] |  |
| Mortality (counting LTFU) | IR^†^ | 39.8 | 21.7 | 27.6 | *<0.001* | 28.4 | 13.9 | 12.8 | *<0.001* |
|  | 95%CI^§^ | [37.6-42.1] | [20.8-22.7] | [26.4-28.8] |  | [26.2-30.9] | [13.1-14.6] | [12.3-13.3] |  |
| WHO-4 event | IR^†^ | 12.9 | 4.0 | 2.0 | *<0.001* | 19.0 | 1.9 | 1.0 | *<0.001* |
|  | 95%CI^§^ | [11.0-15.2] | [3.4-4.6] | [1.6-2.6] |  | [16.2-22.2] | [1.5-2.4] | [0.8-1.2] |  |
| WHO-3 event | IR^†^ | 34.1 | 11.7 | 7.2 | *<0.001* | 32.0 | 4.1 | 1.9 | *<0.001* |
|  | 95%CI^§^ | [30.7-38.0] | [10.6-12.9] | [6.3-8.2] |  | [28.1-36.5] | [3.5-4.8] | [1.6-2.2] |  |
| ***Post-ART**** |  |  |  |  |  |  |  |  |  |
| Mortality | IR^†^ | 22.5 | 5.4 | 1.2 | *<0.001* | 8.7 | 1.4 | 0.4 | *<0.001* |
|  | 95%CI^§^ | [20.6-24.4] | [5.0-5.9] | [1.2-1.3] |  | [7.7-9.4] | [1.3-1.7] | [0.4-0.4] |  |
| Mortality (counting LTFU) | IR^†^ | 48.6 | 22.6 | 15.4 | *<0.001* | 22.0 | 10.5 | 9.3 | *<0.001* |
|  | 95%CI^§^ | [45.9-51.5] | [21.7-23.5] | [15.0-15.8] |  | [20.8-23.2] | [10.1-10.9] | [9.1-9.5] |  |
| WHO-4 event | IR^†^ | 11.4 | 2.7 | 0.9 | *<0.001* | 5.3 | 1.5 | 0.9 | *<0.001* |
|  | 95%CI^§^ | [9.4-13.9] | [2.2-3.3] | [0.7-1.1] |  | [4.3-6.5] | [1.3-1.7] | [0.8-1.0] |  |
| WHO-3 event | IR^†^ | 27.7 | 7.5 | 1.8 | *<0.001* | 8.89 | 2.5 | 0.9 | *<0.001* |
|  | 95%CI^§^ | [24.3-31.5] | [6.7-8.5] | [1.6-2.1] |  | [7.6-10.4] | [2.2-2.9] | [0.8-1.0] |  |

* ART: antiretroviral therapy; ^†^ IR: incidence rate; ^§^ 95%CI: 95% confidence interval; ^¥^ *p*-value was calculated in a Poisson model adjusted for age

**Table C. Time-updated age and CD4 rates of mortality by region in the pre-ART period**

| **Age (years)** | **CD4** | **Region** | **Person-time (years)** | **Number of events** | **IR***  **(per 100 PY)** | **95%CI†** |
| --- | --- | --- | --- | --- | --- | --- |
| 0-2 | <15% | Overall | 1,171.3 | 253 | 21.6 | [19.1-24.4] |
|  |  | Asia-Pacific | 109.1 | 6 | 5.5 | [2.5-12.2] |
|  |  | CCASAnet | 24.1 | 1 | 4.1 | [0.6-29.4] |
|  |  | Central Africa | 15.1 | 2 | 13.2 | [3.3-52.9] |
|  |  | East Africa | 209.9 | 45 | 21.4 | [16.0-28.7] |
|  |  | Southern Africa | 726.0 | 176 | 24.2 | [20.9-28.1] |
|  |  | West Africa | 86.9 | 23 | 26.5 | [17.6-39.8] |
|  | 15-24% | Overall | 3,210.4 | 265 | 8.3 | [7.3-9.3] |
|  |  | Asia-Pacific | 253.2 | 5 | 2.0 | [0.8-4.7] |
|  |  | CCASAnet | 92.0 | 1 | 1.1 | [0.2-7.7] |
|  |  | Central Africa | 12.6 | 0 | 0.0 | - |
|  |  | East Africa | 626.9 | 39 | 6.2 | [4.5-8.5] |
|  |  | Southern Africa | 2,025.3 | 201 | 9.9 | [8.6-11.4] |
|  |  | West Africa | 200.4 | 19 | 9.5 | [6.0-14.9] |
|  | ≥25% | Overall | 2,486.7 | 158 | 6.3 | [5.4-7.4] |
|  |  | Asia-Pacific | 220.7 | 7 | 3.2 | [1.5-6.7] |
|  |  | CCASAnet | 80.7 | 2 | 2.5 | [0.6-9.9] |
|  |  | Central Africa | 11.9 | 0 | 0.0 | - |
|  |  | East Africa | 478.8 | 22 | 4.6 | [3.0-7.0] |
|  |  | Southern Africa | 1,572.5 | 117 | 7.4 | [6.2-8.9] |
|  |  | West Africa | 122.1 | 10 | 8.2 | [4.4-15.2] |
| 2-4 | <15% | Overall | 1,841.8 | 169 | 9.2 | [7.9-10.7] |
|  |  | Asia-Pacific | 330.0 | 21 | 6.4 | [4.2-9.8] |
|  |  | CCASAnet | 40.3 | 2 | 5.0 | [1.2-19.8] |
|  |  | Central Africa | 34.4 | 4 | 11.6 | [4.4-31.0] |
|  |  | East Africa | 326.6 | 35 | 10.7 | [7.7-14.9] |
|  |  | Southern Africa | 947.8 | 90 | 9.5 | [7.7-11.7] |
|  |  | West Africa | 162.8 | 17 | 10.4 | [6.5-16.8] |
|  | 15-24% | Overall | 6,443.4 | 198 | 3.1 | [2.7-3.5] |
|  |  | Asia-Pacific | 631.5 | 2 | 0.3 | [0.1-1.3] |
|  |  | CCASAnet | 149.5 | 1 | 0.7 | [0.1-4.8] |
|  |  | Central Africa | 93.2 | 4 | 4.3 | [1.6-11.4] |
|  |  | East Africa | 1,717.6 | 49 | 2.9 | [2.2-3.8] |
|  |  | Southern Africa | 3,275.3 | 121 | 3.7 | [3.1-4.4] |
|  |  | West Africa | 576.4 | 21 | 3.6 | [2.4-5.6] |
|  | ≥25% | Overall | 4,914.6 | 87 | 1.8 | [1.4-2.2] |
|  |  | Asia-Pacific | 478.0 | 3 | 0.6 | [0.2-2.0] |
|  |  | CCASAnet | 103.1 | 0 | 0.0 | - |
|  |  | Central Africa | 68.5 | 0 | 0.0 | - |
|  |  | East Africa | 1,641.4 | 35 | 2.1 | [1.5-3.0] |
|  |  | Southern Africa | 2,356.1 | 43 | 1.8 | [1.4-2.5] |
|  |  | West Africa | 267.4 | 6 | 2.2 | [1.0-5.0] |

**Table C–continued**

| **Age (years)** | **CD4** | **Region** | **Person-time (years)** | **Number of events** | **IR***  **(per 100 PY)** | **95%CI†** |
| --- | --- | --- | --- | --- | --- | --- |
| 5-9 | <200 | Overall | 1,629.4 | 106 | 6.5 | [5.4-7.9] |
|  |  | Asia-Pacific | 396.2 | 15 | 3.8 | [2.3-6.3] |
|  |  | CCASAnet | 44.2 | 1 | 2.3 | [0.3-16.1] |
|  |  | Central Africa | 29.9 | 2 | 6.7 | [1.7-26.8] |
|  |  | East Africa | 272.9 | 26 | 9.5 | [6.5-14.0] |
|  |  | Southern Africa | 748.2 | 47 | 6.3 | [4.7-8.4] |
|  |  | West Africa | 138.0 | 15 | 10.9 | [6.6-18.0] |
|  | 200-499 | Overall | 7,317.5 | 87 | 1.2 | [1.0-1.5] |
|  |  | Asia-Pacific | 745.3 | 2 | 0.3 | [0.1-1.1] |
|  |  | CCASAnet | 206.5 | 0 | 0.0 | - |
|  |  | Central Africa | 319.3 | 3 | 0.9 | [0.3-2.9] |
|  |  | East Africa | 1,702.6 | 24 | 1.4 | [0.9-2.1] |
|  |  | Southern Africa | 3,811.3 | 48 | 1.3 | [1.0-1.7] |
|  |  | West Africa | 532.5 | 10 | 1.9 | [1.0-3.5] |
|  | ≥500 | Overall | 16,133.7 | 83 | 0.5 | [0.4-0.6] |
|  |  | Asia-Pacific | 1,449.1 | 0 | 0.0 | - |
|  |  | CCASAnet | 458.1 | 0 | 0.0 | - |
|  |  | Central Africa | 1,454.5 | 16 | 1.1 | [0.7-1.8] |
|  |  | East Africa | 5,084.6 | 36 | 0.7 | [0.5-1.0] |
|  |  | Southern Africa | 6,397.1 | 23 | 0.4 | [0.2-0.5] |
|  |  | West Africa | 1,290.2 | 8 | 0.6 | [0.3-1.2] |
| 10-14 | <200 | Overall | 368.0 | 13 | 3.5 | [2.1-6.1] |
|  |  | Asia-Pacific | 84.7 | 2 | 2.4 | [0.6-9.4] |
|  |  | CCASAnet | 27.3 | 0 | 0.0 | - |
|  |  | Central Africa | 4.0 | 0 | 0.0 | - |
|  |  | East Africa | 40.3 | 0 | 0.0 | - |
|  |  | Southern Africa | 159.1 | 9 | 5.7 | [2.9-10.9] |
|  |  | West Africa | 52.5 | 2 | 3.8 | [1.0-15.2] |
|  | 200-499 | Overall | 2,726.5 | 17 | 0.6 | [0.4-1.0] |
|  |  | Asia-Pacific | 306.9 | 0 | 0.0 | - |
|  |  | CCASAnet | 108.2 | 0 | 0.0 | - |
|  |  | Central Africa | 256.7 | 2 | 0.8 | [0.2-3.1] |
|  |  | East Africa | 643.9 | 1 | 0.2 | [0.0-1.1] |
|  |  | Southern Africa | 1,161.8 | 12 | 1.0 | [0.6-1.8] |
|  |  | West Africa | 249.0 | 2 | 0.8 | [0.2-3.2] |
|  | ≥500 | Overall | 3,390.5 | 10 | 0.3 | [0.2-0.6] |
|  |  | Asia-Pacific | 319.0 | 0 | 0.0 | - |
|  |  | CCASAnet | 104.8 | 0 | 0.0 | - |
|  |  | Central Africa | 554.6 | 3 | 0.5 | [0.2-1.7] |
|  |  | East Africa | 956.2 | 3 | 0.3 | [0.1-1.0] |
|  |  | Southern Africa | 1,169.5 | 3 | 0.3 | [0.1-0.8] |
|  |  | West Africa | 286.4 | 1 | 0.4 | [0.1-2.5] |

**Table C–continued**

| **Age (years)** | **CD4** | **Region** | **Person-time (years)** | **Number of events** | **IR***  **(per 100 PY)** | **95%CI†** |
| --- | --- | --- | --- | --- | --- | --- |
| 15-19 | <200 | Overall | 38.7 | 2 | 5.2 | [0.3-20.7] |
|  |  | Asia-Pacific | 1.7 | 0 | 0 | - |
|  |  | CCASAnet | 1.3 | 0 | 0 | - |
|  |  | Central Africa | 2.4 | 0 | 0 | - |
|  |  | East Africa | 1.5 | 0 | 0 | - |
|  |  | Southern Africa | 20.5 | 2 | 9.7 | [2.4-39.0] |
|  |  | West Africa | 11.2 | 0 | 0 | - |
|  | 200-499 | Overall | 158.7 | 1 | 0.6 | [0.1-4.5] |
|  |  | Asia-Pacific | 32.3 | 1 | 3.1 | [0.4-22.0] |
|  |  | CCASAnet | 8.7 | 0 | 0 | - |
|  |  | Central Africa | 24.6 | 0 | 0 | - |
|  |  | East Africa | 26.5 | 0 | 0 | - |
|  |  | Southern Africa | 43.0 | 0 | 0 | - |
|  |  | West Africa | 23.7 | 0 | 0 | - |
|  | ≥500 | Overall | 133.0 | 0 | 0 | - |
|  |  | Asia-Pacific | 14.5 | 0 | 0 | - |
|  |  | CCASAnet | 11.6 | 0 | 0 | - |
|  |  | Central Africa | 19.7 | 0 | 0 | - |
|  |  | East Africa | 24.1 | 0 | 0 | - |
|  |  | Southern Africa | 39.5 | 0 | 0 | - |
|  |  | West Africa | 23.7 | 0 | 0 | - |
| 20-24 | <200 | Overall | 38.7 | 0 | 0 | - |
|  |  | Asia-Pacific | 1.7 | 0 | 0 | - |
|  |  | CCASAnet | 1.3 | 0 | 0 | - |
|  |  | Central Africa | 2.4 | 0 | 0 | - |
|  |  | East Africa | 1.5 | 0 | 0 | - |
|  |  | Southern Africa | 20.5 | 0 | 0 | - |
|  |  | West Africa | 11.2 | 0 | 0 | - |
|  | 200-499 | Overall | 158.7 | 0 | 0 | - |
|  |  | Asia-Pacific | 32.3 | 0 | 0 | - |
|  |  | CCASAnet | 8.7 | 0 | 0 | - |
|  |  | Central Africa | 24.6 | 0 | 0 | - |
|  |  | East Africa | 26.5 | 0 | 0 | - |
|  |  | Southern Africa | 43.0 | 0 | 0 | - |
|  |  | West Africa | 23.7 | 0 | 0 | - |
|  | ≥500 | Overall | 133.0 | 0 | 0 | - |
|  |  | Asia-Pacific | 14.5 | 0 | 0 | - |
|  |  | CCASAnet | 11.6 | 0 | 0 | - |
|  |  | Central Africa | 19.7 | 0 | 0 | - |
|  |  | East Africa | 24.1 | 0 | 0 | - |
|  |  | Southern Africa | 39.5 | 0 | 0 | - |
|  |  | West Africa | 23.7 | 0 | 0 | - |

^*^ IR: incidence rate per 100 person years; ^†^ 95%CI: 95% confidence interval

**Table D. Time-updated age and CD4 rates of mortality by region in the pre-ART period where loss-to-follow-up is counted as death**

| **Age (years)** | **CD4** | **Region** | **Person-time (years)** | **Number of events** | **IR***  **(per 100 PY)** | **95%CI†** |
| --- | --- | --- | --- | --- | --- | --- |
| 0-2 | <15% | Overall | 1,171.3 | 649 | 55.4 | [51.3-59.8] |
|  |  | Asia-Pacific | 109.1 | 9 | 8.2 | [4.3-15.9] |
|  |  | CCASAnet | 24.1 | 2 | 8.3 | [2.1-33.1] |
|  |  | Central Africa | 15.1 | 3 | 19.9 | [6.4-61.6] |
|  |  | East Africa | 209.9 | 97 | 46.2 | [37.9-56.4] |
|  |  | Southern Africa | 726.0 | 510 | 70.2 | [64.4-76.6] |
|  |  | West Africa | 86.9 | 28 | 32.2 | [22.2-46.7] |
|  | 15-24% | Overall | 3,210.4 | 916 | 28.5 | [26.7-30.4] |
|  |  | Asia-Pacific | 253.2 | 13 | 5.1 | [3-8.8.0] |
|  |  | CCASAnet | 92.0 | 4 | 4.3 | [1.6-11.6] |
|  |  | Central Africa | 12.6 | 0 | 0.0 | - |
|  |  | East Africa | 626.9 | 103 | 16.4 | [13.5-19.9] |
|  |  | Southern Africa | 2,025.3 | 753 | 37.2 | [34.6-39.9] |
|  |  | West Africa | 200.4 | 43 | 21.5 | [15.9-28.9] |
|  | ≥25% | Overall | 2,486.7 | 916 | 36.8 | [34.5-39.3] |
|  |  | Asia-Pacific | 220.7 | 14 | 6.3 | [3.8-10.7] |
|  |  | CCASAnet | 80.7 | 5 | 6.2 | [2.6-14.9] |
|  |  | Central Africa | 11.9 | 2 | 16.8 | [4.2-67.3] |
|  |  | East Africa | 478.8 | 117 | 24.4 | [20.4-29.3] |
|  |  | Southern Africa | 1,572.5 | 736 | 46.8 | [43.5-50.3] |
|  |  | West Africa | 122.1 | 42 | 34.4 | [25.4-46.5] |
| 2-4 | <15% | Overall | 1,841.8 | 550 | 29.9 | [27.5-32.5] |
|  |  | Asia-Pacific | 330.0 | 34 | 10.3 | [7.4-14.4] |
|  |  | CCASAnet | 40.3 | 3 | 7.4 | [2.4-23.1] |
|  |  | Central Africa | 34.4 | 13 | 37.8 | [21.9-65.1] |
|  |  | East Africa | 326.6 | 124 | 38.0 | [31.8-45.3] |
|  |  | Southern Africa | 947.8 | 341 | 36.0 | [32.4-40] |
|  |  | West Africa | 162.8 | 35 | 21.5 | [15.4-29.9] |
|  | 15-24% | Overall | 6,443.4 | 1183 | 18.4 | [17.3-19.4] |
|  |  | Asia-Pacific | 631.5 | 21 | 3.3 | [2.2-5.1] |
|  |  | CCASAnet | 149.5 | 5 | 3.3 | [1.4-8.0] |
|  |  | Central Africa | 93.2 | 18 | 19.3 | [12.2-30.7] |
|  |  | East Africa | 1,717.6 | 324 | 18.9 | [16.9-21] |
|  |  | Southern Africa | 3,275.3 | 751 | 22.9 | [21.3-24.6] |
|  |  | West Africa | 576.4 | 64 | 11.1 | [8.7-14.2] |
|  | ≥25% | Overall | 4,914.6 | 649 | 55.4 | [51.3-59.8] |
|  |  | Asia-Pacific | 478.0 | 9 | 8.2 | [4.3-15.9] |
|  |  | CCASAnet | 103.1 | 2 | 8.3 | [2.1-33.1] |
|  |  | Central Africa | 68.5 | 3 | 19.9 | [6.4-61.6] |
|  |  | East Africa | 1,641.4 | 97 | 46.2 | [37.9-56.4] |
|  |  | Southern Africa | 2,356.1 | 510 | 70.2 | [64.4-76.6] |
|  |  | West Africa | 267.4 | 28 | 32.2 | [22.2-46.7] |

**Table D –continued**

| **Age (years)** | **CD4** | **Region** | **Person-time (years)** | **Number of events** | **IR***  **(per 100 PY)** | **95%CI†** |
| --- | --- | --- | --- | --- | --- | --- |
| 5-9 | <200 | Overall | 1,629.4 | 1127 | 22.9 | [21.6-24.3] |
|  |  | Asia-Pacific | 396.2 | 17 | 3.6 | [2.2-5.7] |
|  |  | CCASAnet | 44.2 | 6 | 5.8 | [2.6-13] |
|  |  | Central Africa | 29.9 | 8 | 11.7 | [5.8-23.4] |
|  |  | East Africa | 272.9 | 348 | 21.2 | [19.1-23.5] |
|  |  | Southern Africa | 748.2 | 704 | 29.9 | [27.8-32.2] |
|  |  | West Africa | 138.0 | 44 | 16.5 | [12.2-22.1] |
|  | 200-499 | Overall | 7,317.5 | 530 | 32.5 | [29.9-35.4] |
|  |  | Asia-Pacific | 745.3 | 30 | 7.6 | [5.3-10.8] |
|  |  | CCASAnet | 206.5 | 1 | 2.3 | [0.3-16.1] |
|  |  | Central Africa | 319.3 | 6 | 20.1 | [9.0-44.7] |
|  |  | East Africa | 1,702.6 | 110 | 40.3 | [33.4-48.6] |
|  |  | Southern Africa | 3,811.3 | 358 | 47.9 | [43.1-53.1] |
|  |  | West Africa | 532.5 | 25 | 18.1 | [12.2-26.8] |
|  | ≥500 | Overall | 16,133.7 | 1140 | 15.6 | [14.7-16.5] |
|  |  | Asia-Pacific | 1,449.1 | 13 | 1.7 | [1.0-3.0] |
|  |  | CCASAnet | 458.1 | 1 | 0.5 | [0.1-3.4] |
|  |  | Central Africa | 1,454.5 | 17 | 5.3 | [3.3-8.6] |
|  |  | East Africa | 5,084.6 | 227 | 13.3 | [11.7-15.2] |
|  |  | Southern Africa | 6,397.1 | 845 | 22.2 | [20.7-23.7] |
|  |  | West Africa | 1,290.2 | 37 | 6.9 | [5.0-9.6] |
| 10-14 | <200 | Overall | 368.0 | 2212 | 13.7 | [13.2-14.3] |
|  |  | Asia-Pacific | 84.7 | 17 | 1.2 | [0.7-1.9] |
|  |  | CCASAnet | 27.3 | 3 | 0.7 | [0.2-2.0] |
|  |  | Central Africa | 4.0 | 87 | 6.0 | [4.8-7.4] |
|  |  | East Africa | 40.3 | 571 | 11.2 | [10.3-12.2] |
|  |  | Southern Africa | 159.1 | 1449 | 22.7 | [21.5-23.8] |
|  |  | West Africa | 52.5 | 85 | 6.6 | [5.3-8.1] |
|  | 200-499 | Overall | 2,726.5 | 40 | 10.9 | [8.0-14.8] |
|  |  | Asia-Pacific | 306.9 | 6 | 7.1 | [3.2-15.8] |
|  |  | CCASAnet | 108.2 | 1 | 3.7 | [0.5-26] |
|  |  | Central Africa | 256.7 | 0 | 0.0 | - |
|  |  | East Africa | 643.9 | 6 | 14.9 | [6.7-33.2] |
|  |  | Southern Africa | 1,161.8 | 21 | 13.2 | [8.6-20.2] |
|  |  | West Africa | 249.0 | 6 | 11.4 | [5.1-25.4] |
|  | ≥500 | Overall | 3,390.5 | 254 | 9.3 | [8.2-10.5] |
|  |  | Asia-Pacific | 319.0 | 8 | 2.6 | [1.3-5.2] |
|  |  | CCASAnet | 104.8 | 1 | 0.9 | [0.1-6.6] |
|  |  | Central Africa | 554.6 | 18 | 7.0 | [4.4-11.1] |
|  |  | East Africa | 956.2 | 55 | 8.5 | [6.6-11.1] |
|  |  | Southern Africa | 1,169.5 | 151 | 13.0 | [11.1-15.2] |
|  |  | West Africa | 286.4 | 21 | 8.4 | [5.5-12.9] |

**Table D –continued**

| **Age (years)** | **CD4** | **Region** | **Person-time (years)** | **Number of events** | **IR***  **(per 100 PY)** | **95%CI†** |
| --- | --- | --- | --- | --- | --- | --- |
| 15-19 | <200 | Overall | 38.7 | 9 | 23.3 | [12.1-44.7] |
|  |  | Asia-Pacific | 1.7 | 1 | 58.4 | [8.2-414.4] |
|  |  | CCASAnet | 1.3 | 0 | 0.0 | - |
|  |  | Central Africa | 2.4 | 0 | 0.0 | - |
|  |  | East Africa | 1.5 | 0 | 0.0 | - |
|  |  | Southern Africa | 20.5 | 4 | 19.5 | [7.3-51.9] |
|  |  | West Africa | 11.2 | 4 | 35.7 | [13.4-95] |
|  | 200-499 | Overall | 158.7 | 19 | 12.0 | [7.6-18.8] |
|  |  | Asia-Pacific | 32.3 | 3 | 9.3 | [3-28.8] |
|  |  | CCASAnet | 8.7 | 1 | 11.6 | [1.6-82.0] |
|  |  | Central Africa | 24.6 | 0 | 0.0 | - |
|  |  | East Africa | 26.5 | 0 | 0.0 | - |
|  |  | Southern Africa | 43.0 | 9 | 20.9 | [10.9-40.2] |
|  |  | West Africa | 23.7 | 6 | 25.3 | [11.4-56.4] |
|  | ≥500 | Overall | 133.0 | 15 | 11.3 | [6.8-18.7] |
|  |  | Asia-Pacific | 14.5 | 1 | 6.9 | [1.0-49] |
|  |  | CCASAnet | 11.6 | 3 | 25.8 | [8.3-79.9] |
|  |  | Central Africa | 19.7 | 2 | 10.1 | [2.5-40.6] |
|  |  | East Africa | 24.1 | 4 | 16.6 | [6.2-44.3] |
|  |  | Southern Africa | 39.5 | 4 | 10.1 | [3.8-27] |
|  |  | West Africa | 23.7 | 1 | 4.2 | [0.6-30] |
| 20-24 | <200 | Overall | 38.7 | 0 | 0.0 | - |
|  |  | Asia-Pacific | 1.7 | 0 | 0.0 | - |
|  |  | CCASAnet | 1.3 | 0 | 0.0 | - |
|  |  | Central Africa | 2.4 | 0 | 0.0 | - |
|  |  | East Africa | 1.5 | 0 | 0.0 | - |
|  |  | Southern Africa | 20.5 | 0 | 0.0 | - |
|  |  | West Africa | 11.2 | 0 | 0.0 | - |
|  | 200-499 | Overall | 158.7 | 0 | 0.0 | - |
|  |  | Asia-Pacific | 32.3 | 0 | 0.0 | - |
|  |  | CCASAnet | 8.7 | 0 | 0.0 | - |
|  |  | Central Africa | 24.6 | 0 | 0.0 | - |
|  |  | East Africa | 26.5 | 0 | 0.0 | - |
|  |  | Southern Africa | 43.0 | 0 | 0.0 | - |
|  |  | West Africa | 23.7 | 0 | 0.0 | - |
|  | ≥500 | Overall | 133.0 | 0 | 0.0 | - |
|  |  | Asia-Pacific | 14.5 | 0 | 0.0 | - |
|  |  | CCASAnet | 11.6 | 0 | 0.0 | - |
|  |  | Central Africa | 19.7 | 0 | 0.0 | - |
|  |  | East Africa | 24.1 | 0 | 0.0 | - |
|  |  | Southern Africa | 39.5 | 0 | 0.0 | - |
|  |  | West Africa | 23.7 | 0 | 0.0 | - |

^*^ IR: incidence rate per 100 person years; ^†^ 95%CI: 95% confidence interval

| **Age** | **<2 years** | | | **2-4 years** | | | **5-9 years** | | | **10-14 years** | | | **15-19 years** | | | **Total** |
| --- | --- | --- | --- | --- | --- | --- | --- | --- | --- | --- | --- | --- | --- | --- | --- | --- |
| **CD4 cell count** | **<15%** | **15-24%** | **≥ 25%** | **<15%** | **15-24%** | **≥ 25%** | **<200** | **200-499** | **≥500** | **<200** | **200-499** | **≥500** | **<200** | **200-499** | **≥500** |  |
| **WHO Stage 4 events** |  |  |  |  |  |  |  |  |  |  |  |  |  |  |  |  |
| Reccurent bacterial infection | 10 | 16 | 4 | 12 | 15 | 3 | 15 | 22 | 32 | 1 | 8 | 4 | 0 | 0 | 0 | 142 |
| PCP | 21 | 20 | 6 | 21 | 23 | 5 | 18 | 14 | 15 | 2 | 3 | 2 | 0 | 0 | 0 | 150 |
| HIV wasting syndrome | 15 | 12 | 2 | 17 | 8 | 1 | 46 | 9 | 5 | 5 | 2 | 0 | 0 | 0 | 0 | 122 |
| Visceral herpes simplex infection | 0 | 8 | 4 | 4 | 12 | 4 | 7 | 7 | 25 | 1 | 4 | 2 | 0 | 0 | 0 | 78 |
| Candidiasis (except oral) | 10 | 14 | 9 | 8 | 9 | 5 | 15 | 3 | 6 | 1 | 0 | 1 | 0 | 0 | 0 | 81 |
| HIV encephalopathy | 6 | 3 | 7 | 7 | 1 | 1 | 6 | 4 | 0 | 0 | 0 | 0 | 0 | 0 | 0 | 35 |
| Kaposi sarcoma | 0 | 2 | 1 | 1 | 0 | 0 | 10 | 1 | 4 | 0 | 0 | 0 | 0 | 0 | 0 | 19 |
| Cryoptococcosis extrapulmonary | 2 | 1 | 0 | 0 | 0 | 4 | 3 | 3 | 2 | 1 | 1 | 1 | 0 | 0 | 0 | 18 |
| Histoplasmosis extrapulm | 0 | 0 | 0 | 1 | 0 | 0 | 12 | 1 | 1 | 3 | 0 | 0 | 0 | 0 | 0 | 18 |
| CMV chorioretinitis | 3 | 1 | 1 | 2 | 0 | 1 | 4 | 1 | 0 | 0 | 0 | 0 | 0 | 0 | 0 | 13 |
| Cardiomyopathy and/or nephropathy | 2 | 1 | 0 | 2 | 2 | 1 | 2 | 0 | 2 | 0 | 0 | 0 | 0 | 0 | 0 | 12 |
| Toxoplasmosis brain | 0 | 2 | 2 | 1 | 4 | 0 | 2 | 0 | 0 | 0 | 0 | 0 | 0 | 0 | 0 | 11 |
| Non Hodgkin Lymphoma | 0 | 1 | 0 | 3 | 1 | 1 | 0 | 0 | 2 | 0 | 0 | 0 | 0 | 0 | 0 | 8 |
| Unexplained moderate malnutrition or wasting | 2 | 0 | 1 | 1 | 2 | 0 | 2 | 0 | 0 | 0 | 0 | 0 | 0 | 0 | 0 | 8 |
| Other | 1 | 0 | 2 | 0 | 2 | 0 | 2 | 1 | 0 | 0 | 0 | 0 | 0 | 0 | 0 | 8 |
| **WHO Stage 3 events** |  |  |  |  |  |  |  |  |  |  |  |  |  |  |  |  |
| Oral candidiasis | 95 | 122 | 65 | 91 | 82 | 28 | 84 | 55 | 60 | 7 | 11 | 4 | 1 | 0 | 0 | 705 |
| Unexplained chronic diarrhoea | 20 | 42 | 24 | 35 | 48 | 31 | 43 | 27 | 46 | 2 | 4 | 3 | 0 | 0 | 0 | 325 |
| Recurrent bacterial pneumonia | 13 | 28 | 22 | 21 | 19 | 7 | 21 | 7 | 8 | 0 | 1 | 0 | 0 | 0 | 0 | 147 |
| Unexplained anaemia/neutropaenia/ thrombocytopaenia | 4 | 10 | 6 | 25 | 23 | 14 | 22 | 19 | 19 | 1 | 3 | 3 | 0 | 0 | 0 | 149 |
| Unexplained persistant fever | 2 | 6 | 1 | 13 | 8 | 2 | 19 | 14 | 20 | 1 | 3 | 3 | 0 | 0 | 0 | 92 |
| Unexplained moderate mulnutrition or wasting | 2 | 4 | 5 | 8 | 6 | 2 | 16 | 6 | 4 | 2 | 0 | 1 | 0 | 0 | 0 | 56 |
| Symptomatic lymphoid interstitial pneumonitis | 0 | 6 | 0 | 4 | 5 | 1 | 4 | 4 | 0 | 0 | 2 | 0 | 0 | 0 | 0 | 26 |
| Chronic HIV-associated lung disease | 0 | 0 | 0 | 2 | 2 | 2 | 2 | 1 | 7 | 0 | 2 | 1 | 0 | 0 | 0 | 19 |
| Oral hairy leukoplakia | 0 | 0 | 0 | 1 | 1 | 1 | 0 | 3 | 0 | 0 | 0 | 0 | 0 | 0 | 0 | 6 |
| Stomatitis, gingivitis or periodontitis | 0 | 0 | 0 | 0 | 0 | 0 | 0 | 2 | 1 | 0 | 0 | 0 | 0 | 0 | 0 | 3 |

**Table E. Distribution of first WHO-4 events and first WHO-3 events during follow-up, by time-updated CD4 and age in the pre ART period**

**Table F. Time-updated age and CD4 rates of first occurrence of WHO-4 event by region in the pre-ART period**

| **Age (years)** | **CD4** | **Region** | **Person-time (years)** | **Number of events** | **IR* (per 100 PY)** | **95%CI†** |
| --- | --- | --- | --- | --- | --- | --- |
| 0-2 | <15% | Overall | 439.1 | 72 | 16.4 | [13.0-20.7] |
|  |  | Asia-Pacific | 80.3 | 24 | 29.9 | [20.0-44.6] |
|  |  | CCASAnet | 9.2 | 5 | 54.2 | [22.5-130.1] |
|  |  | Central Africa | 14.5 | 1 | 6.9 | [1.0-49.1] |
|  |  | East Africa | 198.1 | 37 | 18.7 | [13.5-25.8] |
|  |  | Southern Africa | 137.1 | 5 | 3.7 | [1.5-8.8] |
|  | 15-24% | Overall | 1,265.1 | 81 | 6.4 | [5.2-8.0] |
|  |  | Asia-Pacific | 200.6 | 25 | 12.5 | [8.4-18.4] |
|  |  | CCASAnet | 51.0 | 8 | 15.7 | [7.8-31.4] |
|  |  | Central Africa | 12.6 | 0 | 0.0 | - |
|  |  | East Africa | 603.9 | 48 | 8.0 | [6.0-10.6] |
|  |  | Southern Africa | 397.0 | 0 | 0.0 | - |
|  | ≥25% | Overall | 900.7 | 39 | 4.3 | [3.2-5.9] |
|  |  | Asia-Pacific | 154.1 | 10 | 6.5 | [3.5-12.1] |
|  |  | CCASAnet | 54.0 | 11 | 20.4 | [11.3-36.8] |
|  |  | Central Africa | 11.8 | 1 | 8.5 | [1.2-60.1] |
|  |  | East Africa | 469.9 | 12 | 2.6 | [1.5-4.5] |
|  |  | Southern Africa | 210.9 | 5 | 2.4 | [1.0-5.7] |
| 2-4 | <15% | Overall | 736.9 | 80 | 10.9 | [8.7-13.5] |
|  |  | Asia-Pacific | 254.0 | 29 | 11.4 | [7.9-16.4] |
|  |  | CCASAnet | 14.0 | 4 | 28.6 | [10.7-76.2] |
|  |  | Central Africa | 34.1 | 0 | 0.0 | - |
|  |  | East Africa | 304.1 | 44 | 14.5 | [10.8-19.4] |
|  |  | Southern Africa | 131.0 | 3 | 2.3 | [0.7-7.1] |
|  | 15-24% | Overall | 2,760.5 | 79 | 2.9 | [2.3-3.6] |
|  |  | Asia-Pacific | 524.3 | 8 | 1.5 | [0.8-3.1] |
|  |  | CCASAnet | 91.0 | 10 | 11.0 | [5.9-20.4] |
|  |  | Central Africa | 90.0 | 0 | 0.0 | - |
|  |  | East Africa | 1,635.3 | 59 | 3.6 | [2.8-4.7] |
|  |  | Southern Africa | 419.9 | 2 | 0.5 | [0.1-1.9] |
|  | ≥25% | Overall | 2,341.5 | 26 | 1.1 | [0.8-1.6] |
|  |  | Asia-Pacific | 362.9 | 5 | 1.4 | [0.6-3.3] |
|  |  | CCASAnet | 84.8 | 3 | 3.5 | [1.1-11] |
|  |  | Central Africa | 69.7 | 0 | 0.0 | - |
|  |  | East Africa | 1,613.5 | 18 | 1.1 | [0.7-1.8] |
|  |  | Southern Africa | 210.6 | 0 | 0.0 | - |

**Table F–continued**

| **Age (years)** | **CD4** | **Region** | **Person-time (years)** | **Number of events** | **IR* (per 100 PY)** | **95%CI†** |
| --- | --- | --- | --- | --- | --- | --- |
| 5-9 | <200 | Overall | 687.2 | 144 | 20.9 | [17.8-24.7] |
|  |  | Asia-Pacific | 319.9 | 84 | 26.3 | [21.2-32.5] |
|  |  | CCASAnet | 7.5 | 4 | 53.1 | [19.9-141.5] |
|  |  | Central Africa | 29.5 | 2 | 6.8 | [1.7-27.1] |
|  |  | East Africa | 240.9 | 51 | 21.2 | [16.1-27.9] |
|  |  | Southern Africa | 89.4 | 3 | 3.4 | [1.1-10.4] |
|  | 200-499 | Overall | 3,055.8 | 66 | 2.2 | [1.7-2.8] |
|  |  | Asia-Pacific | 670.5 | 16 | 2.4 | [1.5-3.9] |
|  |  | CCASAnet | 54.4 | 5 | 9.2 | [3.8-22.1] |
|  |  | Central Africa | 319.2 | 0 | 0.0 | - |
|  |  | East Africa | 1,593.3 | 44 | 2.8 | [2.1-3.7] |
|  |  | Southern Africa | 418.4 | 1 | 0.2 | [0.0-1.7] |
|  | ≥500 | Overall | 8,286.3 | 94 | 1.1 | [0.9-1.4] |
|  |  | Asia-Pacific | 1,259.4 | 4 | 0.3 | [0.1-0.9] |
|  |  | CCASAnet | 181.2 | 7 | 3.9 | [1.8-8.1] |
|  |  | Central Africa | 1,444.7 | 7 | 0.5 | [0.2-1.0] |
|  |  | East Africa | 4,783.1 | 74 | 1.6 | [1.2-1.9] |
|  |  | Southern Africa | 618.0 | 2 | 0.3 | [0.1-1.3] |
| 10-14 | <200 | Overall | 136.7 | 14 | 10.2 | [6.1-17.3] |
|  |  | Asia-Pacific | 78.2 | 11 | 14.1 | [7.8-25.4] |
|  |  | CCASAnet | 1.3 | 1 | 75.4 | [10.6-535.4] |
|  |  | Central Africa | 4.0 | 0 | 0.0 | - |
|  |  | East Africa | 30.6 | 2 | 6.5 | [1.6-26.1] |
|  |  | Southern Africa | 22.5 | 0 | 0.0 | - |
|  | 200-499 | Overall | 1,248.8 | 18 | 1.4 | [0.9-2.3] |
|  |  | Asia-Pacific | 292.8 | 5 | 1.7 | [0.7-4.1] |
|  |  | CCASAnet | 30.7 | 1 | 3.3 | [0.5-23.1] |
|  |  | Central Africa | 256.7 | 0 | 0.0 | - |
|  |  | East Africa | 581.4 | 12 | 2.1 | [1.2-3.6] |
|  |  | Southern Africa | 87.2 | 0 | 0.0 | - |
|  | ≥500 | Overall | 1,860.5 | 10 | 0.5 | [0.3-1.0] |
|  |  | Asia-Pacific | 304.3 | 1 | 0.3 | [0.1-2.3] |
|  |  | CCASAnet | 57.1 | 0 | 0.0 | - |
|  |  | Central Africa | 553.4 | 0 | 0.0 | - |
|  |  | East Africa | 867.5 | 9 | 1.0 | [0.5-2.0] |
|  |  | Southern Africa | 78.2 | 0 | 0.0 | - |

**Table F–continued**

| **Age (years)** | **CD4** | **Region** | **Person-time (years)** | **Number of events** | **IR* (per 100 PY)** | **95%CI†** |
| --- | --- | --- | --- | --- | --- | --- |
| 15-19 | <200 | Overall | 8.5 | 0 | 0 | - |
|  |  | Asia-Pacific | 1.7 | 0 | 0 | - |
|  |  | CCASAnet | 0.0 | 0 | 0 | - |
|  |  | Central Africa | 2.4 | 0 | 0 | - |
|  |  | East Africa | 1.5 | 0 | 0 | - |
|  |  | Southern Africa | 2.9 | 0 | 0 | - |
|  | 200-499 | Overall | 90.3 | 0 | 0 | - |
|  |  | Asia-Pacific | 32.3 | 0 | 0 | - |
|  |  | CCASAnet | 5.4 | 0 | 0 | - |
|  |  | Central Africa | 24.6 | 0 | 0 | - |
|  |  | East Africa | 24.9 | 0 | 0 | - |
|  |  | Southern Africa | 3.2 | 0 | 0 | - |
|  | ≥500 | Overall | 64.8 | 0 | 0 | - |
|  |  | Asia-Pacific | 14.5 | 0 | 0 | - |
|  |  | CCASAnet | 7.0 | 0 | 0 | - |
|  |  | Central Africa | 19.7 | 0 | 0 | - |
|  |  | East Africa | 21.4 | 0 | 0 | - |
|  |  | West Africa | 0.0 | 0 | 0 | - |
| 20-24 | <200 | Overall | 0.0 | 0 | 0 | - |
|  |  | Asia-Pacific | 0.0 | 0 | 0 | - |
|  |  | CCASAnet | 0.0 | 0 | 0 | - |
|  |  | Central Africa | 0.0 | 0 | 0 | - |
|  |  | East Africa | 0.0 | 0 | 0 | - |
|  |  | Southern Africa | 0.0 | 0 | 0 | - |
|  | 200-499 | Overall | 0.8 | 0 | 0 | - |
|  |  | Asia-Pacific | 0.0 | 0 | 0 | - |
|  |  | CCASAnet | 0.0 | 0 | 0 | - |
|  |  | Central Africa | 0.0 | 0 | 0 | - |
|  |  | East Africa | 0.8 | 0 | 0 | - |
|  |  | Southern Africa | 0.0 | 0 | 0 | - |
|  | ≥500 | Overall | 0.0 | 0 | 0 | - |
|  |  | Asia-Pacific | 0.0 | 0 | 0 | - |
|  |  | CCASAnet | 0.0 | 0 | 0 | - |
|  |  | Central Africa | 0.0 | 0 | 0 | - |
|  |  | East Africa | 0.0 | 0 | 0 | - |
|  |  | Southern Africa | 0.0 | 0 | 0 | - |

^*^ IR: incidence rate per 100 person years; ^†^ 95%CI: 95% confidence interval

**Table G. Time-updated age and CD4 rates of first occurrence of WHO-3 event by region in the pre-ART period**

| **Age (years)** | **CD4** | **Region** | **Person-time (years)** | **Number of events** | **IR* (per 100 PY)** | **95%CI†** |
| --- | --- | --- | --- | --- | --- | --- |
| 0-2 | <15% | Overall | 375.4 | 136 | 36.2 | [30.6-42.9] |
|  |  | Asia-Pacific | 63.6 | 39 | 61.3 | [44.8-83.9] |
|  |  | CCASAnet | 3.9 | 4 | 103.3 | [38.8-275.1] |
|  |  | Central Africa | 14.8 | 0 | 0.0 | - |
|  |  | East Africa | 158.2 | 86 | 54.4 | [44.0-67.2] |
|  |  | Southern Africa | 135.0 | 7 | 5.2 | [2.5-10.9] |
|  | 15-24% | Overall | 1,122.3 | 218 | 19.4 | [17.0-22.2] |
|  |  | Asia-Pacific | 163.8 | 56 | 34.2 | [26.3-44.4] |
|  |  | CCASAnet | 38.1 | 13 | 34.1 | [19.8-58.7] |
|  |  | Central Africa | 12.6 | 0 | 0.0 | - |
|  |  | East Africa | 520.6 | 131 | 25.2 | [21.2-29.9] |
|  |  | Southern Africa | 387.2 | 18 | 4.7 | [2.9-7.4] |
|  | ≥25% | Overall | 826.1 | 123 | 14.9 | [12.5-17.8] |
|  |  | Asia-Pacific | 132.7 | 35 | 26.4 | [18.9-36.7] |
|  |  | CCASAnet | 49.9 | 14 | 28.1 | [16.6-47.4] |
|  |  | Central Africa | 11.6 | 1 | 8.6 | [1.2-61.2] |
|  |  | East Africa | 423.3 | 63 | 14.9 | [11.6-19.1] |
|  |  | Southern Africa | 208.6 | 10 | 4.8 | [2.6-8.9] |
| 2-4 | <15% | Overall | 609.6 | 200 | 32.8 | [28.6-37.7] |
|  |  | Asia-Pacific | 180.0 | 87 | 48.4 | [39.2-59.7] |
|  |  | CCASAnet | 4.5 | 2 | 44.2 | [11.1-176.7] |
|  |  | Central Africa | 33.9 | 3 | 8.9 | [2.9-27.5] |
|  |  | East Africa | 267.2 | 100 | 37.4 | [30.8-45.5] |
|  |  | Southern Africa | 124.2 | 8 | 6.4 | [3.2-12.9] |
|  | 15-24% | Overall | 2,392.3 | 194 | 8.1 | [7.0-9.3] |
|  |  | Asia-Pacific | 404.2 | 38 | 9.4 | [6.8-12.9] |
|  |  | CCASAnet | 73.0 | 9 | 12.3 | [6.4-23.7] |
|  |  | Central Africa | 89.0 | 2 | 2.3 | [0.6-9.0] |
|  |  | East Africa | 1,427.9 | 126 | 8.8 | [7.4-10.5] |
|  |  | Southern Africa | 398.4 | 19 | 4.8 | [3.0-7.5] |
|  | ≥25% | Overall | 2,104.1 | 88 | 4.2 | [3.4-5.2] |
|  |  | Asia-Pacific | 298.1 | 18 | 6.0 | [3.8-9.6] |
|  |  | CCASAnet | 70.5 | 4 | 5.7 | [2.1-15.1] |
|  |  | Central Africa | 65.0 | 0 | 0.0 | - |
|  |  | East Africa | 1,468.3 | 60 | 4.1 | [3.2-5.3] |
|  |  | Southern Africa | 202.3 | 6 | 3.0 | [1.3-6.6] |

**Table G–continued**

| **Age (years)** | **CD4** | **Region** | **Person-time (years)** | **Number of events** | **IR* (per 100 PY)** | **95%CI†** |
| --- | --- | --- | --- | --- | --- | --- |
| 5-9 | <200 | Overall | 573.9 | 211 | 36.8 | [32.1-42.1] |
|  |  | Asia-Pacific | 249.6 | 106 | 42.5 | [35.1-51.4] |
|  |  | CCASAnet | 7.9 | 2 | 25.2 | [6.3-100.8] |
|  |  | Central Africa | 28.6 | 4 | 14.0 | [5.3-37.3] |
|  |  | East Africa | 202.4 | 93 | 46.0 | [37.5-56.3] |
|  |  | Southern Africa | 85.5 | 6 | 7.0 | [3.2-15.6] |
|  | 200-499 | Overall | 2,769.9 | 138 | 5.0 | [4.2-5.9] |
|  |  | Asia-Pacific | 552.1 | 35 | 6.3 | [4.5-8.8] |
|  |  | CCASAnet | 51.2 | 3 | 5.9 | [1.9-18.2] |
|  |  | Central Africa | 309.6 | 4 | 1.3 | [0.5-3.4] |
|  |  | East Africa | 1,460.2 | 82 | 5.6 | [4.5-7.0] |
|  |  | Southern Africa | 396.8 | 14 | 3.5 | [2.1-6.0] |
|  | ≥500 | Overall | 7,777.1 | 165 | 2.1 | [1.8-2.5] |
|  |  | Asia-Pacific | 1,014.5 | 36 | 3.5 | [2.6-4.9] |
|  |  | CCASAnet | 170.6 | 3 | 1.8 | [0.6-5.5] |
|  |  | Central Africa | 1,445.0 | 5 | 0.4 | [0.1-0.8] |
|  |  | East Africa | 4,545.7 | 115 | 2.5 | [2.1-3.0] |
|  |  | Southern Africa | 601.4 | 6 | 1.0 | [0.5-2.2] |
| 10-14 | <200 | Overall | 117.3 | 13 | 11.1 | [6.4-19.1] |
|  |  | Asia-Pacific | 61.7 | 8 | 13.0 | [6.5-25.9] |
|  |  | CCASAnet | 2.2 | 0 | 0.0 | - |
|  |  | Central Africa | 4.0 | 0 | 0.0 | - |
|  |  | East Africa | 27.4 | 4 | 14.6 | [5.5-38.9] |
|  |  | Southern Africa | 22.0 | 1 | 4.5 | [0.6-32.2] |
|  | 200-499 | Overall | 1,157.4 | 26 | 2.3 | [1.5-3.3] |
|  |  | Asia-Pacific | 230.1 | 9 | 3.9 | [2.0-7.5] |
|  |  | CCASAnet | 26.0 | 1 | 3.8 | [0.5-27.3] |
|  |  | Central Africa | 255.5 | 1 | 0.4 | [0.1-2.8] |
|  |  | East Africa | 561.1 | 15 | 2.7 | [1.6-4.4] |
|  |  | Southern Africa | 84.8 | 0 | 0.0 | - |
|  | ≥500 | Overall | 1,765.0 | 15 | 0.9 | [0.5-1.4] |
|  |  | Asia-Pacific | 222.4 | 4 | 1.8 | [0.7-4.8] |
|  |  | CCASAnet | 65.3 | 0 | 0.0 | - |
|  |  | Central Africa | 553.2 | 2 | 0.4 | [0.1-1.5] |
|  |  | East Africa | 845.9 | 9 | 1.1 | [0.6-2.0] |
|  |  | Southern Africa | 78.2 | 0 | 0.0 | - |

**Table G–continued**

| **Age (years)** | **CD4** | **Region** | **Person-time (years)** | **Number of events** | **IR* (per 100 PY)** | **95%CI†** |
| --- | --- | --- | --- | --- | --- | --- |
| 15-19 | <200 | Overall | 8.0 | 0 | 0.0 | - |
|  |  | Asia-Pacific | 1.5 | 0 | 0.0 | - |
|  |  | CCASAnet | 0.0 | 0 | 0.0 | - |
|  |  | Central Africa | 2.4 | 0 | 0.0 | - |
|  |  | East Africa | 1.3 | 0 | 0.0 | - |
|  |  | Southern Africa | 2.9 | 0 | 0.0 | - |
|  | 200-499 | Overall | 72.7 | 1 | 1.4 | [0.2-9.8] |
|  |  | Asia-Pacific | 20.6 | 0 | 0.0 | - |
|  |  | CCASAnet | 4.1 | 0 | 0.0 | - |
|  |  | Central Africa | 24.5 | 1 | 4.1 | [0.6-29.0] |
|  |  | East Africa | 20.4 | 0 | 0.0 | - |
|  |  | Southern Africa | 3.2 | 0 | 0.0 | - |
|  | ≥500 | Overall | 60.5 | 0 | 0.0 | - |
|  |  | Asia-Pacific | 13.1 | 0 | 0.0 | - |
|  |  | CCASAnet | 6.8 | 0 | 0.0 | - |
|  |  | Central Africa | 19.7 | 0 | 0.0 | - |
|  |  | East Africa | 18.7 | 0 | 0.0 | - |
|  |  | West Africa | 0.0 | 0 | 0.0 | - |
| 20-24 | <200 | Overall | 0.0 | 0 | 0.0 | - |
|  |  | Asia-Pacific | 0.0 | 0 | 0.0 | - |
|  |  | CCASAnet | 0.0 | 0 | 0.0 | - |
|  |  | Central Africa | 0.0 | 0 | 0.0 | - |
|  |  | East Africa | 0.0 | 0 | 0.0 | - |
|  |  | Southern Africa | 0.0 | 0 | 0.0 | - |
|  | 200-499 | Overall | 0.0 | 0 | 0.0 | - |
|  |  | Asia-Pacific | 0.0 | 0 | 0.0 | - |
|  |  | CCASAnet | 0.0 | 0 | 0.0 | - |
|  |  | Central Africa | 0.0 | 0 | 0.0 | - |
|  |  | East Africa | 0.0 | 0 | 0.0 | - |
|  |  | Southern Africa | 0.0 | 0 | 0.0 | - |
|  | ≥500 | Overall | 0.0 | 0 | 0.0 | - |
|  |  | Asia-Pacific | 0.0 | 0 | 0.0 | - |
|  |  | CCASAnet | 0.0 | 0 | 0.0 | - |
|  |  | Central Africa | 0.0 | 0 | 0.0 | - |
|  |  | East Africa | 0.0 | 0 | 0.0 | - |
|  |  | Southern Africa | 0.0 | 0 | 0.0 | - |

^*^ IR: incidence rate per 100 person years; ^†^ 95%CI: 95% confidence interval

**Table H. Time-updated age and CD4 rates of first occurrence of TB event by region in the pre-ART period**

| **Age (years)** | **CD4** | **Region** | **Person-time (years)** | **Number of events** | **IR* (per 100 PY)** | **95% CI†** |
| --- | --- | --- | --- | --- | --- | --- |
| 0 - 2 | <15% | Overall | 424.7 | 108 | 25.4 | [21.1-30.7] |
|  |  | Asia-Pacific | 84.2 | 24 | 28.5 | [19.1-42.6] |
|  |  | CCASAnet | 10.0 | 0 | 0.0 | - |
|  |  | Central Africa | 14.2 | 2 | 14.1 | [3.5-56.4] |
|  |  | East Africa | 185.4 | 55 | 29.7 | [22.8-38.6] |
|  |  | South Africa | 131.0 | 27 | 20.6 | [14.1-30.1] |
|  | 15-24% | Overall | 1246.8 | 145 | 11.6 | [9.9-13.7] |
|  |  | Asia-Pacific | 218.5 | 13 | 5.9 | [3.5-10.2] |
|  |  | CCASAnet | 54.0 | 0 | 0.0 | - |
|  |  | Central Africa | 12.3 | 2 | 16.3 | [4.1-65.0] |
|  |  | East Africa | 582.2 | 77 | 13.2 | [10.6-16.5] |
|  |  | South Africa | 379.8 | 53 | 14.0 | [10.7-18.3] |
|  | ≥ 25% | Overall | 898.2 | 56 | 6.2 | [4.8-8.1] |
|  |  | Asia-Pacific | 156.0 | 7 | 4.5 | [2.1-9.4] |
|  |  | CCASAnet | 57.3 | 0 | 0.0 | - |
|  |  | Central Africa | 11.4 | 2 | 17.5 | [4.4-69.9] |
|  |  | East Africa | 466.6 | 25 | 5.4 | [3.6-7.9] |
|  |  | South Africa | 206.8 | 22 | 10.6 | [7-16.2] |
| 2 - 4 | <15% | Overall | 692.6 | 166 | 24.0 | [20.6-27.9] |
|  |  | Asia-Pacific | 246.9 | 46 | 18.6 | [14.0-24.9] |
|  |  | CCASAnet | 15.8 | 0 | 0.0 | - |
|  |  | Central Africa | 32.1 | 9 | 28.0 | [14.6-53.8] |
|  |  | East Africa | 273.7 | 95 | 34.7 | [28.4-42.4] |
|  |  | South Africa | 124.1 | 16 | 12.9 | [7.9-21.1] |
|  | 15-24% | Overall | 2639.2 | 168 | 6.4 | [5.5-7.4] |
|  |  | Asia-Pacific | 490.3 | 28 | 5.7 | [3.9-8.3] |
|  |  | CCASAnet | 105.1 | 0 | 0.0 | - |
|  |  | Central Africa | 89.2 | 6 | 6.7 | [3.0-15.0] |
|  |  | East Africa | 1550.1 | 113 | 7.3 | [6.1-8.8] |
|  |  | South Africa | 404.5 | 21 | 5.2 | [3.4-8.0] |
|  | ≥ 25% | Overall | 2272.3 | 81 | 3.6 | [2.9-4.4] |
|  |  | Asia-Pacific | 342.0 | 21 | 6.1 | [4.0-9.4] |
|  |  | CCASAnet | 92.4 | 1 | 1.1 | [0.2-7.7] |
|  |  | Central Africa | 70.6 | 2 | 2.8 | [0.7-11.3] |
|  |  | East Africa | 1564.7 | 50 | 3.2 | [2.4-4.2] |
|  |  | South Africa | 202.7 | 7 | 3.5 | [1.6-7.2] |

^*^ IR: incidence rate per 100 person years; ^†^ 95%CI: 95% confidence interval

**Table H - continued**

| **Age (years)** | **CD4** | **Region** | **Person-time (years)** | **Number of events** | **IR* (per 100 PY)** | **95% CI†** |
| --- | --- | --- | --- | --- | --- | --- |
| 5 - 9 | <200 | Overall | 622.8 | 183 | 29.4 | [25.4-34.0] |
|  |  | Asia-Pacific | 296.2 | 74 | 25.0 | [19.9-31.4] |
|  |  | CCASAnet | 9.0 | 0 | 0.0 | - |
|  |  | Central Africa | 28.4 | 6 | 21.1 | [9.5-47.1] |
|  |  | East Africa | 204.7 | 87 | 42.5 | [34.5-52.5] |
|  |  | South Africa | 84.5 | 16 | 18.9 | [11.6-30.9] |
|  | 200-499 | Overall | 2824.5 | 208 | 7.4 | [6.4-8.4] |
|  |  | Asia-Pacific | 570.4 | 41 | 7.2 | [5.3-9.8] |
|  |  | CCASAnet | 68.5 | 1 | 1.5 | [0.2-10.4] |
|  |  | Central Africa | 312.1 | 8 | 2.6 | [1.3-5.1] |
|  |  | East Africa | 1464.3 | 136 | 9.3 | [7.9-11.0] |
|  |  | South Africa | 409.2 | 22 | 5.4 | [3.5-8.2] |
|  | ≥ 500 | Overall | 7949.8 | 241 | 3.0 | [2.7-3.4] |
|  |  | Asia-Pacific | 1058.2 | 44 | 4.2 | [3.1-5.6] |
|  |  | CCASAnet | 230.7 | 0 | 0.0 | - |
|  |  | Central Africa | 1441.5 | 17 | 1.2 | [0.7-1.9] |
|  |  | East Africa | 4622.0 | 163 | 3.5 | [3.0-4.1] |
|  |  | South Africa | 597.5 | 17 | 2.8 | [1.8-4.6] |
| 10 - 14 | <200 | Overall | 114.4 | 14 | 12.2 | [7.3-20.7] |
|  |  | Asia-Pacific | 61.4 | 8 | 13.0 | [6.5-26.1] |
|  |  | CCASAnet | 2.0 | 0 | 0.0 | - |
|  |  | Central Africa | 3.4 | 1 | 29.7 | [4.2-211.2] |
|  |  | East Africa | 26.1 | 5 | 19.1 | [8.0-45.9] |
|  |  | South Africa | 21.5 | 0 | 0.0 | - |
|  | 200-499 | Overall | 1128.9 | 21 | 1.9 | [1.2-2.9] |
|  |  | Asia-Pacific | 217.0 | 7 | 3.2 | [1.5-6.8] |
|  |  | CCASAnet | 40.7 | 0 | 0.0 | - |
|  |  | Central Africa | 249.3 | 1 | 0.4 | [0.1-2.8] |
|  |  | East Africa | 541.6 | 12 | 2.2 | [1.3-3.9] |
|  |  | South Africa | 80.3 | 1 | 1.2 | [0.2-8.8] |
|  | ≥ 500 | Overall | 1772.4 | 19 | 1.1 | [0.7-1.7] |
|  |  | Asia-Pacific | 227.3 | 2 | 0.9 | [0.2-3.5] |
|  |  | CCASAnet | 75.0 | 0 | 0.0 | - |
|  |  | Central Africa | 550.8 | 1 | 0.2 | [0.0-1.3] |
|  |  | East Africa | 841.5 | 14 | 1.7 | [1.0-2.8] |
|  |  | South Africa | 77.7 | 2 | 2.6 | [0.6-10.3] |

^*^ IR: incidence rate per 100 person years; ^†^ 95%CI: 95% confidence interval

**Table H - continued**

| **Age (years)** | **CD4** | **Region** | **Person-time (years)** | **Number of events** | **IR* (per 100 PY)** | **95% CI†** |
| --- | --- | --- | --- | --- | --- | --- |
| 15 - 19 | <200 | Overall | 5.5 | 0 | 0.0 | - |
|  |  | Asia-Pacific | 0.0 | 0 | 0.0 | - |
|  |  | CCASAnet | 0.0 | 0 | 0.0 | - |
|  |  | Central Africa | 2.4 | 0 | 0.0 | - |
|  |  | East Africa | 0.2 | 0 | 0.0 | - |
|  |  | South Africa | 2.9 | 0 | 0.0 | - |
|  | 200-499 | Overall | 74.4 | 3 | 4.0 | [1.3-12.5] |
|  |  | Asia-Pacific | 18.3 | 2 | 10.9 | [2.7-43.7] |
|  |  | CCASAnet | 7.7 | 0 | 0.0 | - |
|  |  | Central Africa | 24.6 | 0 | 0.0 | - |
|  |  | East Africa | 20.8 | 0 | 0.0 | - |
|  |  | South Africa | 3.0 | 1 | 33.4 | [4.7-237.1] |
|  | ≥ 500 | Overall | 65.2 | 1 | 1.5 | [0.2-10.9] |
|  |  | Asia-Pacific | 13.3 | 0 | 0.0 | - |
|  |  | CCASAnet | 8.9 | 0 | 0.0 | - |
|  |  | Central Africa | 19.5 | 1 | 5.1 | [0.7-36.3] |
|  |  | East Africa | 21.3 | 0 | 0.0 | - |
|  |  | West Africa | 0.0 | 0 | 0.0 | - |
| 20 - 24 | <200 | Overall | 0.0 | 0 | 0.0 | - |
|  |  | Asia-Pacific | 0.0 | 0 | 0.0 | - |
|  |  | CCASAnet | 0.0 | 0 | 0.0 | - |
|  |  | Central Africa | 0.0 | 0 | 0.0 | - |
|  |  | East Africa | 0.0 | 0 | 0.0 | - |
|  |  | South Africa | 0.0 | 0 | 0.0 | - |
|  | 200-499 | Overall | 0.8 | 0 | 0.0 | - |
|  |  | Asia-Pacific | 0.0 | 0 | 0.0 | - |
|  |  | CCASAnet | 0.0 | 0 | 0.0 | - |
|  |  | Central Africa | 0.0 | 0 | 0.0 | - |
|  |  | East Africa | 0.8 | 0 | 0.0 | - |
|  |  | South Africa | 0.0 | 0 | 0.0 | - |
|  | ≥ 500 | Overall | 0.0 | 0 | 0.0 | - |
|  |  | Asia-Pacific | 0.0 | 0 | 0.0 | - |
|  |  | CCASAnet | 0.0 | 0 | 0.0 | - |
|  |  | Central Africa | 0.0 | 0 | 0.0 | - |
|  |  | East Africa | 0.0 | 0 | 0.0 | - |
|  |  | South Africa | 0.0 | 0 | 0.0 | - |

^*^ IR: incidence rate per 100 person years; ^†^ 95%CI: 95% confidence interval

**Table I. Time-updated age and CD4 rates of mortality by region in the post-ART period**

| **Age (years)** | **CD4** | **Region** | **Person-time (years)** | **Number of events** | **IR* (per 100 PY)** | **95%CI†** |
| --- | --- | --- | --- | --- | --- | --- |
| 0-2 | <15% | Overall | 726.8 | 330 | 45.4 | [40.8-50.6] |
|  |  | Asia-Pacific | 57.2 | 23 | 40.2 | [26.7-60.5] |
|  |  | CCASAnet | 22.9 | 9 | 39.2 | [20.4-75.4] |
|  |  | Central Africa | 6.1 | 1 | 16.4 | [2.3-116.4] |
|  |  | East Africa | 70.2 | 29 | 41.3 | [28.7-59.5] |
|  |  | Southern Africa | 515.4 | 233 | 45.2 | [39.8-51.4] |
|  |  | West Africa | 55.1 | 35 | 63.5 | [45.6-88.5] |
|  | 15-24% | Overall | 3,390.5 | 368 | 10.8 | [9.8-12.0] |
|  |  | Asia-Pacific | 195.5 | 11 | 5.6 | [3.1-10.2] |
|  |  | CCASAnet | 96.1 | 14 | 14.6 | [8.6-24.6] |
|  |  | Central Africa | 23.8 | 1 | 4.2 | [0.6-29.9] |
|  |  | East Africa | 341.0 | 29 | 8.5 | [5.9-12.2] |
|  |  | Southern Africa | 2,511.7 | 279 | 11.1 | [9.9-12.5] |
|  |  | West Africa | 222.5 | 34 | 15.3 | [10.9-21.4] |
|  | ≥25% | Overall | 5,969.7 | 172 | 2.9 | [2.5-3.4] |
|  |  | Asia-Pacific | 490.6 | 7 | 1.4 | [0.7-3.0] |
|  |  | CCASAnet | 187.9 | 4 | 2.1 | [0.8-5.7] |
|  |  | Central Africa | 16.7 | 0 | 0.0 | - |
|  |  | East Africa | 605.6 | 15 | 2.5 | [1.5-4.1] |
|  |  | Southern Africa | 4,364.6 | 133 | 3.1 | [2.6-3.6] |
|  |  | West Africa | 304.2 | 13 | 4.3 | [2.5-7.4] |
| 2-4 | <15% | Overall | 1,702.8 | 216 | 12.7 | [11.1-14.5] |
|  |  | Asia-Pacific | 366.0 | 31 | 8.5 | [6.0-12.0] |
|  |  | CCASAnet | 72.6 | 7 | 9.6 | [4.6-20.2] |
|  |  | Central Africa | 44.4 | 4 | 9.0 | [3.4-24.0] |
|  |  | East Africa | 302.0 | 56 | 18.5 | [14.3-24.1] |
|  |  | Southern Africa | 768.7 | 92 | 12.0 | [9.8-14.7] |
|  |  | West Africa | 148.6 | 26 | 17.5 | [11.9-25.7] |
|  | 15-24% | Overall | 8,334.9 | 265 | 3.2 | [2.8-3.6] |
|  |  | Asia-Pacific | 836.7 | 22 | 2.6 | [1.7-4.0] |
|  |  | CCASAnet | 259.9 | 22 | 8.5 | [5.6-12.9] |
|  |  | Central Africa | 191.9 | 4 | 2.1 | [0.8-5.6] |
|  |  | East Africa | 1,437.6 | 53 | 3.7 | [2.8-4.8] |
|  |  | Southern Africa | 4,855.3 | 129 | 2.7 | [2.2-3.2] |
|  |  | West Africa | 753.5 | 35 | 4.6 | [3.3-6.5] |
|  | ≥25% | Overall | 28,065.6 | 222 | 0.8 | [0.7-0.9] |
|  |  | Asia-Pacific | 1,990.0 | 7 | 0.4 | [0.2-0.7] |
|  |  | CCASAnet | 780.5 | 8 | 1.0 | [0.5-2.1] |
|  |  | Central Africa | 422.3 | 3 | 0.7 | [0.2-2.2] |
|  |  | East Africa | 3,893.1 | 36 | 0.9 | [0.7-1.3] |
|  |  | Southern Africa | 18,779.1 | 138 | 0.7 | [0.6-0.9] |
|  |  | West Africa | 2,200.6 | 30 | 1.4 | [1.0-2.0] |

**Table I –continued**

| **Age (years)** | **CD4** | **Region** | **Person-time (years)** | **Number of events** | **IR* (per 100 PY)** | **95%CI†** |
| --- | --- | --- | --- | --- | --- | --- |
| 5-9 | <200 | Overall | 2,901.8 | 376 | 13.0 | [11.7-14.3] |
|  |  | Asia-Pacific | 374.9 | 58 | 15.5 | [12.0-20.0] |
|  |  | CCASAnet | 112.8 | 17 | 15.1 | [9.4-24.3] |
|  |  | Central Africa | 49.2 | 8 | 16.3 | [8.1-32.5] |
|  |  | East Africa | 532.8 | 68 | 12.8 | [10.1-16.2] |
|  |  | Southern Africa | 1,476.9 | 131 | 8.9 | [7.5-10.5] |
|  |  | West Africa | 355.2 | 94 | 26.5 | [21.6-32.4] |
|  | 200-499 | Overall | 12,041.3 | 223 | 1.9 | [1.6-2.1] |
|  |  | Asia-Pacific | 1,294.1 | 22 | 1.7 | [1.1-2.6] |
|  |  | CCASAnet | 596.6 | 2 | 0.3 | [0.1-1.3] |
|  |  | Central Africa | 374.9 | 9 | 2.4 | [1.3-4.6] |
|  |  | East Africa | 2,169.8 | 47 | 2.2 | [1.6-2.9] |
|  |  | Southern Africa | 6,366.1 | 108 | 1.7 | [1.4-2.1] |
|  |  | West Africa | 1,239.8 | 35 | 2.8 | [2.0-3.9] |
|  | ≥500 | Overall | 75,815.0 | 278 | 0.4 | [0.3-0.4] |
|  |  | Asia-Pacific | 7,200.4 | 15 | 0.2 | [0.1-0.4] |
|  |  | CCASAnet | 2,581.5 | 19 | 0.7 | [0.5-1.2] |
|  |  | Central Africa | 2,848.4 | 13 | 0.5 | [0.3-0.8] |
|  |  | East Africa | 11,576.4 | 52 | 0.5 | [0.3-0.6] |
|  |  | Southern Africa | 44,953.3 | 136 | 0.3 | [0.3-0.4] |
|  |  | West Africa | 6,655.0 | 43 | 0.7 | [0.5-0.9] |
| 10-14 | <200 | Overall | 2,026.8 | 107 | 5.3 | [4.4-6.4] |
|  |  | Asia-Pacific | 218.8 | 14 | 6.4 | [3.8-10.8] |
|  |  | CCASAnet | 201.2 | 11 | 5.5 | [3.0-9.9] |
|  |  | Central Africa | 41.8 | 4 | 9.6 | [3.6-25.5] |
|  |  | East Africa | 402.4 | 24 | 6.0 | [4.0-8.9] |
|  |  | Southern Africa | 879.4 | 32 | 3.6 | [2.6-5.2] |
|  |  | West Africa | 283.2 | 22 | 7.8 | [5.1-11.8] |
|  | 200-499 | Overall | 12,030.1 | 129 | 1.1 | [0.9-1.3] |
|  |  | Asia-Pacific | 1,161.0 | 9 | 0.8 | [0.4-1.5] |
|  |  | CCASAnet | 789.6 | 10 | 1.3 | [0.7-2.4] |
|  |  | Central Africa | 505.6 | 1 | 0.2 | [0.0-1.4] |
|  |  | East Africa | 2,069.5 | 33 | 1.6 | [1.1-2.2] |
|  |  | Southern Africa | 6,271.9 | 51 | 0.8 | [0.6-1.1] |
|  |  | West Africa | 1,232.4 | 25 | 2.0 | [1.4-3.0] |
|  | ≥500 | Overall | 44,239.9 | 154 | 0.4 | [0.3-0.4] |
|  |  | Asia-Pacific | 5,571.8 | 10 | 0.2 | [0.1-0.3] |
|  |  | CCASAnet | 1,860.7 | 9 | 0.5 | [0.3-0.9] |
|  |  | Central Africa | 2,297.1 | 3 | 0.1 | [0.0-0.4] |
|  |  | East Africa | 5,920.7 | 25 | 0.4 | [0.3-0.6] |
|  |  | Southern Africa | 24,831.4 | 69 | 0.3 | [0.2-0.4] |
|  |  | West Africa | 3,758.3 | 38 | 1.0 | [0.7-1.4] |

**Table I –continued**

| **Age (years)** | **CD4** | **Region** | **Person-time (years)** | **Number of events** | **IR* (per 100 PY)** | **95%CI†** |
| --- | --- | --- | --- | --- | --- | --- |
| 15-19 | <200 | Overall | 748.5 | 11 | 1.5 | [0.8-2.7] |
|  |  | Asia-Pacific | 111.8 | 4 | 3.6 | [1.3-9.5] |
|  |  | CCASAnet | 152.3 | 4 | 2.6 | [1.0-7.0] |
|  |  | Central Africa | 13.9 | 0 | 0.0 | - |
|  |  | East Africa | 98.5 | 0 | 0.0 | - |
|  |  | Southern Africa | 271.5 | 1 | 0.4 | [0.1-2.6] |
|  |  | West Africa | 100.4 | 2 | 2.0 | [0.5-8.0] |
|  | 200-499 | Overall | 3,047.4 | 33 | 1.1 | [0.8-1.5] |
|  |  | Asia-Pacific | 463.7 | 3 | 0.7 | [0.2-2.0] |
|  |  | CCASAnet | 293.2 | 2 | 0.7 | [0.2-2.7] |
|  |  | Central Africa | 172.5 | 1 | 0.6 | [0.1-4.1] |
|  |  | East Africa | 306.0 | 2 | 0.7 | [0.2-2.6] |
|  |  | Southern Africa | 1,519.8 | 11 | 0.7 | [0.4-1.3] |
|  |  | West Africa | 292.3 | 14 | 4.8 | [2.8-8.1] |
|  | ≥500 | Overall | 6,604.1 | 41 | 0.6 | [0.5-0.8] |
|  |  | Asia-Pacific | 1,421.0 | 8 | 0.6 | [0.3-1.1] |
|  |  | CCASAnet | 576.4 | 6 | 1.0 | [0.5-2.3] |
|  |  | Central Africa | 435.7 | 2 | 0.5 | [0.1-1.8] |
|  |  | East Africa | 550.4 | 3 | 0.6 | [0.2-1.7] |
|  |  | Southern Africa | 3,169.8 | 17 | 0.5 | [0.3-0.9] |
|  |  | West Africa | 450.9 | 5 | 1.1 | [0.5-2.7] |
| 20-24 | <200 | Overall | 53.0 | 2 | 3.8 | [0.9-15.1] |
|  |  | Asia-Pacific | 8.1 | 1 | 12.3 | [1.7-87.2] |
|  |  | CCASAnet | 33.6 | 1 | 3.0 | [0.4-21.1] |
|  |  | Central Africa | - | 0 | 0.0 | - |
|  |  | East Africa | - | 0 | 0.0 | - |
|  |  | Southern Africa | 11.2 | 0 | 0.0 | - |
|  |  | West Africa | - | 0 | 0.0 | - |
|  | 200-499 | Overall | 75.9 | 1 | 1.3 | [0.2-9.3] |
|  |  | Asia-Pacific | 14.6 | 0 | 0.0 | - |
|  |  | CCASAnet | 36.5 | 1 | 2.7 | [0.4-19.4] |
|  |  | Central Africa | 0.9 | 0 | 0.0 | - |
|  |  | East Africa | 1.8 | 0 | 0.0 | - |
|  |  | Southern Africa | 12.8 | 0 | 0.0 | - |
|  |  | West Africa | 9.3 | 0 | 0.0 | - |
|  | ≥500 | Overall | 171.4 | 4 | 2.3 | [0.9-6.2] |
|  |  | Asia-Pacific | 56.5 | 0 | 0.0 | - |
|  |  | CCASAnet | 61.2 | 2 | 3.3 | [0.8-13.1] |
|  |  | Central Africa | 3.3 | 0 | 0.0 | - |
|  |  | East Africa | 1.7 | 0 | 0.0 | - |
|  |  | Southern Africa | 36.9 | 1 | 2.7 | [0.4-19.3] |
|  |  | West Africa | 11.7 | 1 | 8.5 | [1.2-60.6] |

^*^ IR: incidence rate per 100 person years; ^†^ 95%CI: 95% confidence interval

**Table J. Time-updated age and CD4 rates of mortality by region in the post-ART period where loss-to-follow-up is defined as mortality**

| **Age (years)** | **CD4** | **Region** | **Person-time (years)** | **Number of events** | **IR* (per 100 PY)** | **95%CI†** |
| --- | --- | --- | --- | --- | --- | --- |
| 0-2 | <15% | Overall | 726.8 | 604 | 83.1 | [76.7-90.0] |
|  |  | Asia-Pacific | 57.2 | 29 | 50.7 | [35.2-73.0] |
|  |  | CCASAnet | 22.9 | 9 | 39.2 | [20.4-75.4] |
|  |  | Central Africa | 6.1 | 1 | 16.4 | [2.3-116.4] |
|  |  | East Africa | 70.2 | 62 | 88.4 | [68.9-113.3] |
|  |  | Southern Africa | 515.4 | 454 | 88.1 | [80.4-96.6] |
|  |  | West Africa | 55.1 | 49 | 89.0 | [67.2-117.7] |
|  | 15-24% | Overall | 3,390.5 | 971 | 28.6 | [26.9-30.5] |
|  |  | Asia-Pacific | 195.5 | 18 | 9.2 | [5.8-14.6] |
|  |  | CCASAnet | 96.1 | 23 | 23.9 | [15.0.9-36] |
|  |  | Central Africa | 23.8 | 1 | 4.2 | [0.6-29.9] |
|  |  | East Africa | 341.0 | 96 | 28.2 | [23.0-34.4] |
|  |  | Southern Africa | 2,511.7 | 769 | 30.6 | [28.5-32.9] |
|  |  | West Africa | 222.5 | 64 | 28.8 | [22.5-36.8] |
|  | ≥25% | Overall | 5,969.7 | 725 | 12.1 | [11.3-13.1] |
|  |  | Asia-Pacific | 490.6 | 25 | 5.1 | [3.4-7.5] |
|  |  | CCASAnet | 187.9 | 20 | 10.6 | [6.9-16.5] |
|  |  | Central Africa | 16.7 | 0 | 0.0 | - |
|  |  | East Africa | 605.6 | 69 | 11.4 | [9.0-14.4] |
|  |  | Southern Africa | 4,364.6 | 583 | 13.4 | [12.3-14.5] |
|  |  | West Africa | 304.2 | 28 | 9.2 | [6.4-13.3] |
| 2-4 | <15% | Overall | 1,702.8 | 577 | 33.9 | [31.2-36.8] |
|  |  | Asia-Pacific | 366.0 | 54 | 14.7 | [11.3-19.2] |
|  |  | CCASAnet | 72.6 | 17 | 23.4 | [14.5-37.6] |
|  |  | Central Africa | 44.4 | 11 | 24.8 | [13.7-44.8] |
|  |  | East Africa | 302.0 | 117 | 38.7 | [32.3-46.4] |
|  |  | Southern Africa | 768.7 | 331 | 43.1 | [38.7-48.0] |
|  |  | West Africa | 148.6 | 47 | 31.6 | [23.8-42.1] |
|  | 15-24% | Overall | 8,334.9 | 1676 | 20.1 | [19.2-21.1] |
|  |  | Asia-Pacific | 836.7 | 93 | 11.1 | [9.1-13.6] |
|  |  | CCASAnet | 259.9 | 57 | 21.9 | [16.9-28.4] |
|  |  | Central Africa | 191.9 | 27 | 14.1 | [9.7-20.5] |
|  |  | East Africa | 1,437.6 | 264 | 18.4 | [16.3-20.7] |
|  |  | Southern Africa | 4,855.3 | 1105 | 22.8 | [21.5-24.1] |
|  |  | West Africa | 753.5 | 130 | 17.3 | [14.5-20.5] |
|  | ≥25% | Overall | 28,065.6 | 4523 | 16.1 | [15.7-16.6] |
|  |  | Asia-Pacific | 1,990.0 | 243 | 12.2 | [10.8-13.8] |
|  |  | CCASAnet | 780.5 | 120 | 15.4 | [12.9-18.4] |
|  |  | Central Africa | 422.3 | 61 | 14.4 | [11.2-18.6] |
|  |  | East Africa | 3,893.1 | 532 | 13.7 | [12.6-14.9] |
|  |  | Southern Africa | 18,779.1 | 3213 | 17.1 | [16.5-17.7] |
|  |  | West Africa | 2,200.6 | 354 | 16.1 | [14.5-17.9] |

**Table J –continued**

| **Age (years)** | **CD4** | **Region** | **Person-time (years)** | **Number of events** | **IR* (per 100 PY)** | **95%CI†** |
| --- | --- | --- | --- | --- | --- | --- |
| 5-9 | <200 | Overall | 2,901.8 | 844 | 29.1 | [27.2-31.1] |
|  |  | Asia-Pacific | 374.9 | 79 | 21.1 | [16.9-26.3] |
|  |  | CCASAnet | 112.8 | 24 | 21.3 | [14.3-31.7] |
|  |  | Central Africa | 49.2 | 11 | 22.4 | [12.4-40.4] |
|  |  | East Africa | 532.8 | 171 | 32.1 | [27.6-37.3] |
|  |  | Southern Africa | 1,476.9 | 421 | 28.5 | [25.9-31.4] |
|  |  | West Africa | 355.2 | 138 | 38.9 | [32.9-45.9] |
|  | 200-499 | Overall | 12,041.3 | 1310 | 10.9 | [10.3-11.5] |
|  |  | Asia-Pacific | 1,294.1 | 48 | 3.7 | [2.8-4.9] |
|  |  | CCASAnet | 596.6 | 15 | 2.5 | [1.5-4.2] |
|  |  | Central Africa | 374.9 | 35 | 9.3 | [6.7-13.0] |
|  |  | East Africa | 2,169.8 | 268 | 12.4 | [11.0-13.9] |
|  |  | Southern Africa | 6,366.1 | 843 | 13.2 | [12.4-14.2] |
|  |  | West Africa | 1,239.8 | 101 | 8.1 | [6.7-9.9] |
|  | ≥500 | Overall | 75,815.0 | 5841 | 7.7 | [7.5-7.9] |
|  |  | Asia-Pacific | 7,200.4 | 302 | 4.2 | [3.7-4.7] |
|  |  | CCASAnet | 2,581.5 | 95 | 3.7 | [3.0-4.5] |
|  |  | Central Africa | 2,848.4 | 161 | 5.7 | [4.8-6.6] |
|  |  | East Africa | 11,576.4 | 897 | 7.7 | [7.3-8.3] |
|  |  | Southern Africa | 44,953.3 | 4090 | 9.1 | [8.8-9.4] |
|  |  | West Africa | 6,655.0 | 296 | 4.4 | [4.0-5.0] |
| 10-14 | <200 | Overall | 2,026.8 | 348 | 17.2 | [15.5-19.1] |
|  |  | Asia-Pacific | 218.8 | 21 | 9.6 | [6.3-14.7] |
|  |  | CCASAnet | 201.2 | 15 | 7.5 | [4.5-12.4] |
|  |  | Central Africa | 41.8 | 8 | 19.1 | [9.6-38.3] |
|  |  | East Africa | 402.4 | 58 | 14.4 | [11.1-18.6] |
|  |  | Southern Africa | 879.4 | 205 | 23.3 | [20.3-26.7] |
|  |  | West Africa | 283.2 | 41 | 14.5 | [10.7-19.7] |
|  | 200-499 | Overall | 12,030.1 | 1122 | 9.3 | [8.8-9.9] |
|  |  | Asia-Pacific | 1,161.0 | 46 | 4.0 | [3.0-5.3] |
|  |  | CCASAnet | 789.6 | 29 | 3.7 | [2.6-5.3] |
|  |  | Central Africa | 505.6 | 29 | 5.7 | [4.0-8.3] |
|  |  | East Africa | 2,069.5 | 181 | 8.7 | [7.6-10.1] |
|  |  | Southern Africa | 6,271.9 | 746 | 11.9 | [11.1-12.8] |
|  |  | West Africa | 1,232.4 | 91 | 7.4 | [6.0-9.1] |
|  | ≥500 | Overall | 44,239.9 | 4705 | 10.6 | [10.3-10.9] |
|  |  | Asia-Pacific | 5,571.8 | 300 | 5.4 | [4.8-6.0] |
|  |  | CCASAnet | 1,860.7 | 136 | 7.3 | [6.2-8.6] |
|  |  | Central Africa | 2,297.1 | 175 | 7.6 | [6.6-8.8] |
|  |  | East Africa | 5,920.7 | 518 | 8.7 | [8.0-9.5] |
|  |  | Southern Africa | 24,831.4 | 3101 | 12.5 | [12.1-12.9] |
|  |  | West Africa | 3,758.3 | 475 | 12.6 | [11.6-13.8] |

**Table J –continued**

| **Age (years)** | **CD4** | **Region** | **Person-time (years)** | **Number of events** | **IR* (per 100 PY)** | **95%CI†** |
| --- | --- | --- | --- | --- | --- | --- |
| 15-19 | <200 | Overall | 748.5 | 67 | 9.0 | [7.0-11.4] |
|  |  | Asia-Pacific | 111.8 | 12 | 10.7 | [6.1-18.9] |
|  |  | CCASAnet | 152.3 | 7 | 4.6 | [2.2-9.6] |
|  |  | Central Africa | 13.9 | 1 | 7.2 | [1.0-50.9] |
|  |  | East Africa | 98.5 | 4 | 4.1 | [1.5-10.8] |
|  |  | Southern Africa | 271.5 | 34 | 12.5 | [8.9-17.5] |
|  |  | West Africa | 100.4 | 9 | 9.0 | [4.7-17.2] |
|  | 200-499 | Overall | 3,047.4 | 393 | 12.9 | [11.7-14.2] |
|  |  | Asia-Pacific | 463.7 | 44 | 9.5 | [7.1-12.7] |
|  |  | CCASAnet | 293.2 | 33 | 11.3 | [8.0-15.8] |
|  |  | Central Africa | 172.5 | 12 | 7.0 | [4.0-12.3] |
|  |  | East Africa | 306.0 | 31 | 10.1 | [7.1-14.4] |
|  |  | Southern Africa | 1,519.8 | 224 | 14.7 | [12.9-16.8] |
|  |  | West Africa | 292.3 | 49 | 16.8 | [12.7-22.2] |
|  | ≥500 | Overall | 6,604.1 | 1184 | 17.9 | [16.9-19] |
|  |  | Asia-Pacific | 1,421.0 | 142 | 10.0 | [8.5-11.8] |
|  |  | CCASAnet | 576.4 | 122 | 21.2 | [17.7-25.3] |
|  |  | Central Africa | 435.7 | 60 | 13.8 | [10.7-17.7] |
|  |  | East Africa | 550.4 | 62 | 11.3 | [8.8-14.4] |
|  |  | Southern Africa | 3,169.8 | 701 | 22.1 | [20.5-23.8] |
|  |  | West Africa | 450.9 | 97 | 21.5 | [17.6-26.2] |
| 20-24 | <200 | Overall | 53.0 | 2 | 3.8 | [0.9-15.1] |
|  |  | Asia-Pacific | 8.1 | 1 | 12.3 | [1.7-87.2] |
|  |  | CCASAnet | 33.6 | 1 | 3.0 | [0.4-21.1] |
|  |  | Central Africa | - | 0 | 0.0 | - |
|  |  | East Africa | - | 0 | 0.0 | - |
|  |  | Southern Africa | 11.2 | 0 | 0.0 | - |
|  |  | West Africa | - | 0 | 0.0 | - |
|  | 200-499 | Overall | 75.9 | 21 | 27.7 | [18.0-42.4] |
|  |  | Asia-Pacific | 14.6 | 5 | 34.2 | [14.2-82.2] |
|  |  | CCASAnet | 36.5 | 14 | 38.3 | [22.7-64.8] |
|  |  | Central Africa | 0.9 | 0 | 0.0 | - |
|  |  | East Africa | 1.8 | 0 | 0.0 | - |
|  |  | Southern Africa | 12.8 | 0 | 0.0 | - |
|  |  | West Africa | 9.3 | 2 | 21.4 | [5.4-85.6] |
|  | ≥500 | Overall | 171.4 | 56 | 32.7 | [25.1-42.5] |
|  |  | Asia-Pacific | 56.5 | 17 | 30.1 | [18.7-48.4] |
|  |  | CCASAnet | 61.2 | 18 | 29.4 | [18.5-46.7] |
|  |  | Central Africa | 3.3 | 2 | 60.0 | [15.0-240.0] |
|  |  | East Africa | 1.7 | 0 | 0.0 | - |
|  |  | Southern Africa | 36.9 | 16 | 43.4 | [26.6-70.9] |
|  |  | West Africa | 11.7 | 3 | 25.6 | [8.3-79.4] |

^*^ IR: incidence rate per 100 person years; ^†^ 95%CI: 95% confidence interval

**Table K . Distribution of first WHO-4 events and first WHO-3 events during follow-up, by time-updated CD4 and age in the post ART period**

| **Age** | **<2 years** | | | **2-4 years** | | | **5-9 years** | | | **10-14 years** | | | **15-19 years** | | | **20-24 years** | | | **Total** |
| --- | --- | --- | --- | --- | --- | --- | --- | --- | --- | --- | --- | --- | --- | --- | --- | --- | --- | --- | --- |
| **CD4 cell count** | **<15%** | **15-24%** | **≥ 25%** | **<15%** | **15-24%** | **≥ 25%** | **<200** | **200-499** | **≥500** | **<200** | **200-499** | **≥500** | **<200** | **200-499** | **≥500** | **<200** | **200-499** | **≥500** |  |
| **WHO Stage 4 events** |  |  |  |  |  |  |  |  |  |  |  |  |  |  |  |  |  |  |  |
| Reccurent bacterial infection | 3 | 8 | 4 | 13 | 21 | 32 | 11 | 21 | 117 | 6 | 21 | 64 | 1 | 5 | 5 | 1 | 0 | 0 | 333 |
| PCP | 10 | 11 | 4 | 19 | 8 | 3 | 11 | 7 | 19 | 4 | 4 | 6 | 0 | 2 | 3 | 0 | 0 | 0 | 111 |
| HIV wasting syndrome | 2 | 2 | 2 | 7 | 4 | 3 | 5 | 1 | 7 | 2 | 1 | 2 | 0 | 1 | 0 | 0 | 0 | 0 | 39 |
| Visceral herpes simplex infection | 1 | 3 | 4 | 7 | 15 | 5 | 6 | 11 | 43 | 3 | 6 | 20 | 2 | 1 | 5 | 0 | 0 | 0 | 132 |
| Candidiasis (except oral) | 0 | 3 | 0 | 11 | 3 | 3 | 9 | 6 | 11 | 4 | 2 | 8 | 0 | 2 | 2 | 0 | 0 | 0 | 64 |
| HIV encephalopathy | 6 | 9 | 10 | 6 | 2 | 9 | 3 | 2 | 8 | 0 | 1 | 1 | 0 | 0 | 0 | 0 | 0 | 0 | 57 |
| Kaposi sarcoma | 0 | 0 | 0 | 0 | 0 | 1 | 6 | 2 | 2 | 1 | 3 | 2 | 0 | 0 | 0 | 0 | 0 | 0 | 17 |
| Cryoptococcosis extrapulmonary | 1 | 2 | 0 | 2 | 3 | 2 | 6 | 2 | 6 | 1 | 4 | 6 | 0 | 2 | 1 | 0 | 0 | 0 | 38 |
| Histoplasmosis extrapulm | 0 | 0 | 0 | 1 | 0 | 0 | 5 | 6 | 1 | 0 | 1 | 1 | 0 | 0 | 0 | 0 | 0 | 0 | 15 |
| CMV chorioretinitis | 0 | 1 | 1 | 4 | 0 | 1 | 1 | 0 | 3 | 0 | 0 | 1 | 0 | 1 | 0 | 0 | 0 | 0 | 13 |
| Cardiomyopathy and/or nephropathy | 0 | 0 | 0 | 0 | 1 | 0 | 2 | 3 | 1 | 1 | 0 | 2 | 0 | 1 | 1 | 0 | 0 | 0 | 12 |
| Toxoplasmosis brain | 1 | 0 | 0 | 0 | 2 | 2 | 1 | 3 | 10 | 1 | 2 | 1 | 0 | 1 | 1 | 0 | 0 | 0 | 25 |
| Non Hodgkin Lymphoma | 0 | 0 | 0 | 2 | 1 |  | 1 | 1 | 1 | 6 | 0 | 0 | 1 | 0 | 1 | 0 | 0 | 0 | 14 |
| Unexplained moderate malnutrition or wasting | 1 | 1 | 0 | 0 | 0 | 0 | 0 | 0 | 1 | 0 | 0 | 0 | 0 | 0 | 0 | 0 | 0 | 0 | 3 |
| Other | 0 | 2 | 1 | 6 | 0 | 2 | 3 | 2 | 5 | 2 | 2 | 2 | 0 | 7 | 5 | 0 | 0 | 0 | 39 |
| **WHO Stage 3 events** |  |  |  |  |  |  |  |  |  |  |  |  |  |  |  |  |  |  |  |
| Oral candidiasis | 24 | 37 | 22 | 57 | 55 | 27 | 37 | 38 | 74 | 14 | 16 | 25 | 1 | 1 | 5 | 0 | 0 | 0 | 433 |
| Unexplained chronic diarrhoea | 15 | 12 | 10 | 30 | 62 | 29 | 26 | 42 | 58 | 4 | 10 | 17 | 0 | 1 | 3 | 0 | 0 | 0 | 319 |
| Recurrent bacterial pneumonia | 16 | 30 | 18 | 47 | 27 | 37 | 38 | 40 | 69 | 4 | 20 | 19 | 0 | 0 | 0 | 0 | 0 | 0 | 365 |
| Unexplained anaemia/neutropaenia/ thrombocytopaenia | 2 | 3 | 2 | 13 | 12 | 8 | 8 | 6 | 31 | 0 | 6 | 9 | 1 | 2 | 1 | 0 | 0 | 0 | 104 |
| Unexplained persistant fever | 0 | 2 | 3 | 5 | 8 | 6 | 9 | 7 | 20 | 2 | 6 | 6 | 1 | 2 | 1 | 0 | 0 | 0 | 78 |
| Unexplained moderate mulnutrition or wasting | 1 | 5 | 3 | 3 | 0 | 4 | 4 | 5 | 8 | 2 | 2 | 4 | 0 | 1 | 0 | 0 | 0 | 0 | 42 |
| Symptomatic lymphoid interstitial pneumonitis | 2 | 2 | 0 | 8 | 8 | 0 | 3 | 6 | 4 | 0 | 0 | 0 | 0 | 0 | 0 | 0 | 0 | 0 | 33 |
| Chronic HIV-associated lung disease | 0 | 1 | 3 | 1 | 1 | 5 | 6 | 7 | 0 | 3 | 4 | 0 | 0 | 0 | 0 | 0 | 0 | 0 | 31 |
| Oral hairy leukoplakia | 0 | 0 | 0 | 0 | 0 | 0 | 0 | 0 | 1 | 0 | 0 | 0 | 0 | 0 | 0 | 0 | 0 | 0 | 1 |
| Stomatitis, gingivitis or periodontitis | 0 | 0 | 0 | 0 | 0 | 0 | 0 | 1 | 1 | 0 | 1 | 1 | 0 | 0 | 0 | 0 | 0 | 0 | 4 |

**Table L. Time-updated age and CD4 rates of first occurrence of WHO-4 event by region in the post-ART period**

| **Age (years)** | **CD4** | **Region** | **Person-time (years)** | **Number of events** | **IR* (per 100 PY)** | **95%CI†** |
| --- | --- | --- | --- | --- | --- | --- |
| 0-2 | <15% | Overall | 190.2 | 25 | 13.1 | [8.9-19.4] |
|  |  | Asia-Pacific | 29.7 | 1 | 3.4 | [0.5-23.9] |
|  |  | CCASAnet | 15.1 | 9 | 59.5 | [31.0-114.3] |
|  |  | Central Africa | 6.1 | 0 | 0.0 | - |
|  |  | East Africa | 68.8 | 12 | 17.4 | [9.9-30.7] |
|  |  | Southern Africa | 70.5 | 3 | 4.3 | [1.4-13.2] |
|  | 15-24% | Overall | 883.9 | 41 | 4.6 | [3.4-6.3] |
|  |  | Asia-Pacific | 101.3 | 7 | 6.9 | [3.3-14.5] |
|  |  | CCASAnet | 50.7 | 17 | 33.5 | [20.8-53.9] |
|  |  | Central Africa | 23.8 | 0 | 0.0 | - |
|  |  | East Africa | 336.1 | 15 | 4.5 | [2.7-7.4] |
|  |  | Southern Africa | 372.1 | 2 | 0.5 | [0.1-2.2] |
|  | ≥25% | Overall | 1,497.4 | 25 | 1.7 | [1.1-2.5] |
|  |  | Asia-Pacific | 171.3 | 4 | 2.3 | [0.9-6.2] |
|  |  | CCASAnet | 83.6 | 13 | 15.6 | [9.0-26.8] |
|  |  | Central Africa | 16.7 | 0 | 0.0 | - |
|  |  | East Africa | 540.3 | 8 | 1.5 | [0.7-3.0] |
|  |  | Southern Africa | 685.5 | 0 | 0.0 | - |
| 2-4 | <15% | Overall | 677.4 | 74 | 10.9 | [8.7-13.7] |
|  |  | Asia-Pacific | 233.0 | 13 | 5.6 | [3.2-9.6] |
|  |  | CCASAnet | 40.0 | 18 | 45.0 | [28.4-71.4] |
|  |  | Central Africa | 44.4 | 0 | 0.0 | - |
|  |  | East Africa | 278.5 | 43 | 15.4 | [11.4-20.8] |
|  |  | Southern Africa | 81.3 | 0 | 0.0 | - |
|  | 15-24% | Overall | 2,820.0 | 60 | 2.1 | [1.7-2.7] |
|  |  | Asia-Pacific | 522.9 | 3 | 0.6 | [0.2-1.8] |
|  |  | CCASAnet | 141.9 | 19 | 13.4 | [8.5-21.0] |
|  |  | Central Africa | 191.9 | 1 | 0.5 | [0.1-3.7] |
|  |  | East Africa | 1,391.2 | 37 | 2.7 | [1.9-3.7] |
|  |  | Southern Africa | 572.1 | 0 | 0.0 | - |
|  | ≥25% | Overall | 8,286.8 | 63 | 0.8 | [0.6-1.0] |
|  |  | Asia-Pacific | 1,038.8 | 8 | 0.8 | [0.4-1.5] |
|  |  | CCASAnet | 381.6 | 18 | 4.7 | [3.0-7.5] |
|  |  | Central Africa | 421.7 | 0 | 0.0 | - |
|  |  | East Africa | 3,763.7 | 37 | 1.0 | [0.7-1.4] |
|  |  | Southern Africa | 2,680.9 | 0 | 0.0 | - |

**Table L–continued**

| **Age (years)** | **CD4** | **Region** | **Person-time (years)** | **Number of events** | **IR* (per 100 PY)** | **95%CI†** |
| --- | --- | --- | --- | --- | --- | --- |
| 5-9 | <200 | Overall | 899.1 | 69 | 7.7 | [6.1-9.7] |
|  |  | Asia-Pacific | 272.5 | 24 | 8.8 | [5.9-13.1] |
|  |  | CCASAnet | 26.2 | 11 | 42.0 | [23.3-75.8] |
|  |  | Central Africa | 49.2 | 0 | 0.0 | - |
|  |  | East Africa | 448.3 | 34 | 7.6 | [5.4-10.6] |
|  |  | Southern Africa | 102.9 | 0 | 0.0 | - |
|  | 200-499 | Overall | 3,946.7 | 67 | 1.7 | [1.3-2.2] |
|  |  | Asia-Pacific | 974.9 | 13 | 1.3 | [0.8-2.3] |
|  |  | CCASAnet | 208.2 | 9 | 4.3 | [2.3-8.3] |
|  |  | Central Africa | 371.9 | 1 | 0.3 | [0.0-1.9] |
|  |  | East Africa | 1,982.4 | 44 | 2.2 | [1.7-3.0] |
|  |  | Southern Africa | 409.2 | 0 | 0.0 | - |
|  | ≥500 | Overall | 25,233.2 | 237 | 0.9 | [0.8-1.1] |
|  |  | Asia-Pacific | 5,065.7 | 17 | 0.3 | [0.2-0.5] |
|  |  | CCASAnet | 938.0 | 45 | 4.8 | [3.6-6.4] |
|  |  | Central Africa | 2,814.1 | 10 | 0.4 | [0.2-0.7] |
|  |  | East Africa | 10,751.9 | 163 | 1.5 | [1.3-1.8] |
|  |  | Southern Africa | 5,663.6 | 2 | 0.0 | [0.0-0.1] |
| 10-14 | <200 | Overall | 637.3 | 25 | 3.9 | [2.7-5.8] |
|  |  | Asia-Pacific | 188.3 | 8 | 4.3 | [2.1-8.5] |
|  |  | CCASAnet | 47.7 | 7 | 14.7 | [7.0-30.8] |
|  |  | Central Africa | 39.2 | 0 | 0.0 | - |
|  |  | East Africa | 310.3 | 10 | 3.2 | [1.7-6.0] |
|  |  | Southern Africa | 51.8 | 0 | 0.0 | - |
|  | 200-499 | Overall | 4,175.1 | 47 | 1.1 | [0.9-1.5] |
|  |  | Asia-Pacific | 1,024.5 | 7 | 0.7 | [0.3-1.4] |
|  |  | CCASAnet | 243.9 | 5 | 2.1 | [0.9-4.9] |
|  |  | Central Africa | 503.8 | 0 | 0.0 | - |
|  |  | East Africa | 1,857.0 | 35 | 1.9 | [1.4-2.6] |
|  |  | Southern Africa | 545.8 | 0 | 0.0 | - |
|  | ≥500 | Overall | 16,004.6 | 116 | 0.7 | [0.6-0.9] |
|  |  | Asia-Pacific | 4,718.8 | 8 | 0.2 | [0.1-0.3] |
|  |  | CCASAnet | 663.8 | 12 | 1.8 | [1.0-3.2] |
|  |  | Central Africa | 2,280.4 | 10 | 0.4 | [0.2-0.8] |
|  |  | East Africa | 5,315.3 | 86 | 1.6 | [1.3-2.0] |
|  |  | Southern Africa | 3,026.2 | 0 | 0.0 | - |

**Table L –continued**

| **Age (years)** | **CD4** | **Region** | **Person-time (years)** | **Number of events** | **IR* (per 100 PY)** | **95%CI†** |
| --- | --- | --- | --- | --- | --- | --- |
| 15-19 | <200 | Overall | 273.4 | 3 | 1.1 | [0.4-3.4] |
|  |  | Asia-Pacific | 91.4 | 2 | 2.2 | [0.6-8.8] |
|  |  | CCASAnet | 43.4 | 0 | 0.0 | - |
|  |  | Central Africa | 13.9 | 0 | 0.0 | - |
|  |  | East Africa | 80.3 | 1 | 1.2 | [0.2-8.8] |
|  |  | Southern Africa | 44.3 | 0 | 0.0 | - |
|  | 200-499 | Overall | 1,131.8 | 23 | 2.0 | [1.4-3.1] |
|  |  | Asia-Pacific | 442.1 | 5 | 1.1 | [0.5-2.7] |
|  |  | CCASAnet | 100.4 | 10 | 10.0 | [5.4-18.5] |
|  |  | Central Africa | 166.9 | 1 | 0.6 | [0.1-4.3] |
|  |  | East Africa | 250.9 | 7 | 2.8 | [1.3-5.8] |
|  |  | Southern Africa | 171.5 | 0 | 0.0 | - |
|  | ≥500 | Overall | 3,034.4 | 23 | 0.8 | [0.5-1.1] |
|  |  | Asia-Pacific | 1,323.3 | 3 | 0.2 | [0.1-0.7] |
|  |  | CCASAnet | 233.2 | 9 | 3.9 | [2.0-7.4] |
|  |  | Central Africa | 433.6 | 1 | 0.2 | [0.0-1.6] |
|  |  | East Africa | 475.5 | 10 | 2.1 | [1.1-3.9] |
|  |  | West Africa | 0.0 | 0 | 0.0 | - |
| 20-24 | <200 | Overall | 23.1 | 0 | 0.0 | - |
|  |  | Asia-Pacific | 8.0 | 0 | 0.0 | - |
|  |  | CCASAnet | 10.1 | 0 | 0.0 | - |
|  |  | Central Africa | 0.0 | 0 | 0.0 | - |
|  |  | East Africa | 0.0 | 0 | 0.0 | - |
|  |  | Southern Africa | 5.1 | 0 | 0.0 | - |
|  | 200-499 | Overall | 32.7 | 0 | 0.0 | - |
|  |  | Asia-Pacific | 14.6 | 0 | 0.0 | - |
|  |  | CCASAnet | 12.8 | 0 | 0.0 | - |
|  |  | Central Africa | 0.9 | 0 | 0.0 | - |
|  |  | East Africa | 1.8 | 0 | 0.0 | - |
|  |  | Southern Africa | 2.6 | 0 | 0.0 | - |
|  | ≥500 | Overall | 97.5 | 1 | 1.0 | [0.1-7.3] |
|  |  | Asia-Pacific | 50.7 | 0 | 0.0 | - |
|  |  | CCASAnet | 27.8 | 1 | 3.6 | [0.5-25.5] |
|  |  | Central Africa | 3.3 | 0 | 0.0 | - |
|  |  | East Africa | 1.3 | 0 | 0.0 | - |
|  |  | Southern Africa | 14.4 | 0 | 0.0 | - |

^*^ IR: incidence rate per 100 person years; ^†^ 95%CI: 95% confidence interval

**Table M. Time-updated age and CD4 rates of first occurrence of WHO-3 event by region in the post-ART period**

| **Age (years)** | **CD4** | **Region** | **Person-time (years)** | **Number of events** | **IR* (per 100 PY)** | **95%CI†** |
| --- | --- | --- | --- | --- | --- | --- |
| 0-2 | <15% | Overall | 182.7 | 60 | 32.9 | [25.5-42.3] |
|  |  | Asia-Pacific | 28.1 | 12 | 42.8 | [24.3-75.3] |
|  |  | CCASAnet | 13.6 | 13 | 95.9 | [55.7-165.2] |
|  |  | Central Africa | 6.1 | 0 | 0.0 | - |
|  |  | East Africa | 64.3 | 33 | 51.4 | [36.5-72.2] |
|  |  | Southern Africa | 70.7 | 2 | 2.8 | [0.7-11.3] |
|  | 15-24% | Overall | 853.2 | 91 | 10.7 | [8.7-13.1] |
|  |  | Asia-Pacific | 94.3 | 9 | 9.6 | [5.0-18.4] |
|  |  | CCASAnet | 45.3 | 25 | 55.2 | [37.3-81.6] |
|  |  | Central Africa | 23.8 | 0 | 0.0 | - |
|  |  | East Africa | 320.3 | 50 | 15.6 | [11.8-20.6] |
|  |  | Southern Africa | 369.5 | 7 | 1.9 | [0.9-4.0] |
|  | ≥25% | Overall | 1,455.0 | 59 | 4.0 | [3.1-5.2] |
|  |  | Asia-Pacific | 166.8 | 10 | 6.0 | [3.2-11.1] |
|  |  | CCASAnet | 80.5 | 11 | 13.7 | [7.6-24.7] |
|  |  | Central Africa | 16.7 | 0 | 0.0 | - |
|  |  | East Africa | 508.2 | 36 | 7.1 | [5.1-9.8] |
|  |  | Southern Africa | 682.9 | 2 | 0.3 | [0.1-1.2] |
| 2-4 | <15% | Overall | 634.3 | 166 | 26.2 | [22.5-30.5] |
|  |  | Asia-Pacific | 215.0 | 62 | 28.8 | [22.4-36.9] |
|  |  | CCASAnet | 31.6 | 21 | 66.3 | [43.3-101.8] |
|  |  | Central Africa | 44.4 | 2 | 4.5 | [1.1-18.0] |
|  |  | East Africa | 262.0 | 75 | 28.6 | [22.8-35.9] |
|  |  | Southern Africa | 81.0 | 6 | 7.4 | [3.3-16.5] |
|  | 15-24% | Overall | 2,651.0 | 173 | 6.5 | [5.6-7.6] |
|  |  | Asia-Pacific | 475.3 | 23 | 4.8 | [3.2-7.3] |
|  |  | CCASAnet | 118.9 | 24 | 20.2 | [13.5-30.1] |
|  |  | Central Africa | 189.1 | 1 | 0.5 | [0.1-3.8] |
|  |  | East Africa | 1,299.5 | 114 | 8.8 | [7.3-10.5] |
|  |  | Southern Africa | 568.2 | 11 | 1.9 | [1.1-3.5] |
|  | ≥25% | Overall | 7866.1 | 112 | 1.4 | [1.2-1.7] |
|  |  | Asia-Pacific | 974.3 | 20 | 2.1 | [1.3-3.2] |
|  |  | CCASAnet | 356.6 | 25 | 7.0 | [4.7-10.4] |
|  |  | Central Africa | 418.4 | 1 | 0.2 | [0.0-1.7] |
|  |  | East Africa | 3,474.8 | 61 | 1.8 | [1.4-2.3] |
|  |  | Southern Africa | 2,641.9 | 5 | 0.2 | [0.1-0.5] |

**Table M–continued**

| **Age (years)** | **CD4** | **Region** | **Person-time (years)** | **Number of events** | **IR* (per 100 PY)** | **95%CI†** |
| --- | --- | --- | --- | --- | --- | --- |
| 5-9 | <200 | Overall | 868.5 | 131 | 15.1 | [12.7-17.9] |
|  |  | Asia-Pacific | 258.6 | 55 | 21.3 | [16.3-27.7] |
|  |  | CCASAnet | 27.5 | 11 | 40.0 | [22.1-72.2] |
|  |  | Central Africa | 49.0 | 2 | 4.1 | [1.0-16.3] |
|  |  | East Africa | 431.1 | 60 | 13.9 | [10.8-17.9] |
|  |  | Southern Africa | 102.2 | 3 | 2.9 | [1.0-9.1] |
|  | 200-499 | Overall | 3,730.4 | 151 | 4.0 | [3.5-4.8] |
|  |  | Asia-Pacific | 905.4 | 41 | 4.5 | [3.3-6.2] |
|  |  | CCASAnet | 192.2 | 18 | 9.4 | [5.9-14.9] |
|  |  | Central Africa | 366.9 | 5 | 1.4 | [0.6-3.3] |
|  |  | East Africa | 1,864.0 | 85 | 4.6 | [3.7-5.6] |
|  |  | Southern Africa | 401.9 | 2 | 0.5 | [0.1-2.0] |
|  | ≥500 | Overall | 24,174.0 | 272 | 1.1 | [1.0-1.3] |
|  |  | Asia-Pacific | 4590.1 | 62 | 1.4 | [1.1-1.7] |
|  |  | CCASAnet | 850.0 | 35 | 4.1 | [3.0-5.7] |
|  |  | Central Africa | 2,805.5 | 12 | 0.4 | [0.2-0.8] |
|  |  | East Africa | 10,335.0 | 160 | 1.6 | [1.3-1.8] |
|  |  | Southern Africa | 5,593.4 | 3 | 0.1 | [0.0-0.2] |
| 10-14 | <200 | Overall | 609.1 | 27 | 4.4 | [3.0-6.5] |
|  |  | Asia-Pacific | 177.6 | 14 | 7.9 | [4.7-13.3] |
|  |  | CCASAnet | 47.3 | 0 | 0.0 | - |
|  |  | Central Africa | 41.8 | 0 | 0.0 | - |
|  |  | East Africa | 290.6 | 13 | 4.5 | [2.6-7.7] |
|  |  | Southern Africa | 51.8 | 0 | 0.0 | - |
|  | 200-499 | Overall | 3,989.8 | 64 | 1.6 | [1.3-2.1] |
|  |  | Asia-Pacific | 950.2 | 11 | 1.2 | [0.6-2.1] |
|  |  | CCASAnet | 245.6 | 14 | 5.7 | [3.4-9.6] |
|  |  | Central Africa | 501.2 | 1 | 0.2 | [0.0-1.4] |
|  |  | East Africa | 1,765.8 | 37 | 2.1 | [1.5-2.9] |
|  |  | Southern Africa | 526.9 | 1 | 0.2 | [0.0-1.4] |
|  | ≥500 | Overall | 15,520.8 | 84 | 0.5 | [0.4-0.7] |
|  |  | Asia-Pacific | 4,366.2 | 15 | 0.3 | [0.2-0.6] |
|  |  | CCASAnet | 652.0 | 6 | 0.9 | [0.4-2.1] |
|  |  | Central Africa | 2,268.3 | 10 | 0.4 | [0.2-0.8] |
|  |  | East Africa | 5,237.6 | 51 | 1.0 | [0.7-1.3] |
|  |  | Southern Africa | 2,996.8 | 2 | 0.1 | [0.0-0.3] |

**Table M –continued**

| **Age (years)** | **CD4** | **Region** | **Person-time (years)** | **Number of events** | **IR* (per 100 PY)** | **95%CI†** |
| --- | --- | --- | --- | --- | --- | --- |
| 15-19 | <200 | Overall | 2,996.8 | 3 | 1.0 | [0.3-3.1] |
|  |  | Asia-Pacific | 298.9 | 3 | 3.2 | [1.0-9.8] |
|  |  | CCASAnet | 95.3 | 0 | 0.0 | - |
|  |  | Central Africa | 66.6 | 0 | 0.0 | - |
|  |  | East Africa | 12.4 | 0 | 0.0 | - |
|  |  | Southern Africa | 83.0 | 0 | 0.0 | - |
|  | 200-499 | Overall | 41.5 | 7 | 0.6 | [0.3-1.3] |
|  |  | Asia-Pacific | 1,126.8 | 1 | 0.2 | [0.0-1.7] |
|  |  | CCASAnet | 409.1 | 0 | 0.0 | - |
|  |  | Central Africa | 119.0 | 3 | 1.8 | [0.6-5.5] |
|  |  | East Africa | 169.3 | 3 | 1.1 | [0.4-3.5] |
|  |  | Southern Africa | 265.0 | 0 | 0.0 | - |
|  | ≥500 | Overall | 164.3 | 10 | 0.3 | [0.2-0.6] |
|  |  | Asia-Pacific | 2,955.4 | 2 | 0.2 | [0.0-0.7] |
|  |  | CCASAnet | 1,236.9 | 0 | 0.0 | - |
|  |  | Central Africa | 261.1 | 5 | 1.2 | [0.5-2.9] |
|  |  | East Africa | 421.8 | 3 | 0.6 | [0.2-2.0] |
|  |  | West Africa | 473.4 | 0 | 0.0 | - |
| 20-24 | <200 | Overall | 0.0 | 0 | 0.0 | - |
|  |  | Asia-Pacific | 35.9 | 0 | 0.0 | - |
|  |  | CCASAnet | 6.8 | 0 | 0.0 | - |
|  |  | Central Africa | 24.1 | 0 | 0.0 | - |
|  |  | East Africa | 0.0 | 0 | 0.0 | - |
|  |  | Southern Africa | 0.0 | 0 | 0.0 | - |
|  | 200-499 | Overall | 5.1 | 0 | 0.0 | - |
|  |  | Asia-Pacific | 35.6 | 0 | 0.0 | - |
|  |  | CCASAnet | 13.7 | 0 | 0.0 | - |
|  |  | Central Africa | 16.7 | 0 | 0.0 | - |
|  |  | East Africa | 0.9 | 0 | 0.0 | - |
|  |  | Southern Africa | 1.8 | 0 | 0.0 | - |
|  | ≥500 | Overall | 2.6 | 0 | 0.0 | - |
|  |  | Asia-Pacific | 98.3 | 0 | 0.0 | - |
|  |  | CCASAnet | 46.2 | 0 | 0.0 | - |
|  |  | Central Africa | 33.6 | 0 | 0.0 | - |
|  |  | East Africa | 3.3 | 0 | 0.0 | - |
|  |  | Southern Africa | 1.3 | 0 | 0.0 | - |

^*^ IR: incidence rate per 100 person years; ^†^ 95%CI: 95% confidence interval

**Table N. Time-updated age and CD4 rates of first occurrence of WHO-3 event by region in the post-ART period**

| **Age (years)** | **CD4** | **Region** | **Person-time (years)** | **Number of events** | **IR* (per 100 PY)** | **95% CI†** |
| --- | --- | --- | --- | --- | --- | --- |
| 0 - 2 | <15% | Overall | 188.4 | 27 | 14.3 | [9.8-20.9] |
|  |  | Asia-Pacific | 28.9 | 4 | 13.8 | [5.2-36.9] |
|  |  | CCASAnet | 17.9 | 0 | 0.0 | - |
|  |  | Central Africa | 6.1 | 1 | 16.4 | [2.3-116.4] |
|  |  | East Africa | 66.5 | 17 | 25.6 | [15.9-41.1] |
|  |  | South Africa | 69.0 | 5 | 7.2 | [3.0-17.4] |
|  | 15-24% | Overall | 880.9 | 35 | 4.0 | [2.9-5.5] |
|  |  | Asia-Pacific | 100.7 | 5 | 5.0 | [2.1-11.9] |
|  |  | CCASAnet | 58.9 | 0 | 0.0 | - |
|  |  | Central Africa | 23.3 | 1 | 4.3 | [0.6-30.4] |
|  |  | East Africa | 334.0 | 20 | 6.0 | [3.9-9.3] |
|  |  | South Africa | 364.0 | 9 | 2.5 | [1.3-4.8] |
|  | ≥ 25% | Overall | 1492.2 | 28 | 1.9 | [1.3-2.7] |
|  |  | Asia-Pacific | 171.1 | 4 | 2.3 | [0.9-6.2] |
|  |  | CCASAnet | 92.9 | 0 | 0.0 | - |
|  |  | Central Africa | 16.6 | 0 | 0.0 | - |
|  |  | East Africa | 535.3 | 12 | 2.2 | [1.3-3.9] |
|  |  | South Africa | 676.2 | 12 | 1.8 | [1.0-3.1] |
| 2 - 4 | <15% | Overall | 677.2 | 108 | 15.9 | [13.2-19.3] |
|  |  | Asia-Pacific | 233.1 | 17 | 7.3 | [4.5-11.7] |
|  |  | CCASAnet | 54.2 | 0 | 0.0 | - |
|  |  | Central Africa | 43.7 | 12 | 27.5 | [15.6-48.4] |
|  |  | East Africa | 266.1 | 70 | 26.3 | [20.8-33.2] |
|  |  | South Africa | 80.2 | 9 | 11.2 | [5.8-21.6] |
|  | 15-24% | Overall | 2775.9 | 95 | 3.4 | [2.8-4.2] |
|  |  | Asia-Pacific | 517.6 | 2 | 0.4 | [0.1-1.5] |
|  |  | CCASAnet | 182.9 | 0 | 0.0 | - |
|  |  | Central Africa | 182.5 | 11 | 6.0 | [3.3-10.9] |
|  |  | East Africa | 1341.9 | 72 | 5.4 | [4.3-6.8] |
|  |  | South Africa | 551.0 | 10 | 1.8 | [1.0-3.4] |
|  | ≥ 25% | Overall | 8227.4 | 66 | 0.8 | [0.6-1.0] |
|  |  | Asia-Pacific | 1039.5 | 5 | 0.5 | [0.2-1.2] |
|  |  | CCASAnet | 484.9 | 0 | 0.0 | - |
|  |  | Central Africa | 394.2 | 4 | 1.0 | [0.4-2.7] |
|  |  | East Africa | 3692.1 | 46 | 1.2 | [0.9-1.7] |
|  |  | South Africa | 2616.7 | 11 | 0.4 | [0.2-0.8] |

^*^ IR: incidence rate per 100 person years; ^†^ 95%CI: 95% confidence interval

**Table N - continued**

| **Age (years)** | **CD4** | **Region** | **Person-time (years)** | **Number of events** | **IR* (per 100 PY)** | **95% CI†** |
| --- | --- | --- | --- | --- | --- | --- |
| 5 - 9 | <200 | Overall | 1348.8 | 102 | 7.6 | [6.2-9.2] |
|  |  | Asia-Pacific | 345.0 | 18 | 5.2 | [3.3-8.3] |
|  |  | CCASAnet | 72.7 | 0 | 0.0 | - |
|  |  | Central Africa | 93.9 | 9 | 9.6 | [5.0-18.4] |
|  |  | East Africa | 690.6 | 68 | 9.8 | [7.8-12.5] |
|  |  | South Africa | 146.5 | 7 | 4.8 | [2.3-10.0] |
|  | 200-499 | Overall | 3837.9 | 124 | 3.2 | [2.7-3.9] |
|  |  | Asia-Pacific | 952.7 | 22 | 2.3 | [1.5-3.5] |
|  |  | CCASAnet | 282.5 | 1 | 0.4 | [0.0-2.5] |
|  |  | Central Africa | 350.6 | 5 | 1.4 | [0.6-3.4] |
|  |  | East Africa | 1852.4 | 88 | 4.8 | [3.9-5.9] |
|  |  | South Africa | 399.8 | 8 | 2.0 | [1.0-4.0] |
|  | ≥ 500 | Overall | 24801.0 | 268 | 1.1 | [1.0-1.2] |
|  |  | Asia-Pacific | 5029.4 | 17 | 0.3 | [0.2-0.5] |
|  |  | CCASAnet | 1333.4 | 0 | 0.0 | - |
|  |  | Central Africa | 2740.4 | 20 | 0.7 | [0.5-1.1] |
|  |  | East Africa | 10257.4 | 209 | 2.0 | [1.8-2.3] |
|  |  | South Africa | 5440.4 | 22 | 0.4 | [0.3-0.6] |
| 10 - 14 | <200 | Overall | 719.4 | 27 | 3.8 | [2.6-5.5] |
|  |  | Asia-Pacific | 205.8 | 7 | 3.4 | [1.6-7.1] |
|  |  | CCASAnet | 94.4 | 0 | 0.0 | - |
|  |  | Central Africa | 47.9 | 0 | 0.0 | - |
|  |  | East Africa | 322.7 | 17 | 5.3 | [3.3-8.5] |
|  |  | South Africa | 48.5 | 3 | 6.2 | [2.0-19.2] |
|  | 200-499 | Overall | 4103.0 | 74 | 1.8 | [1.4-2.3] |
|  |  | Asia-Pacific | 991.5 | 5 | 0.5 | [0.2-1.2] |
|  |  | CCASAnet | 388.6 | 1 | 0.3 | [0.0-1.8] |
|  |  | Central Africa | 476.7 | 5 | 1.0 | [0.4-2.5] |
|  |  | East Africa | 1729.7 | 57 | 3.3 | [2.5-4.3] |
|  |  | South Africa | 516.4 | 6 | 1.2 | [0.5-2.6] |
|  | ≥ 500 | Overall | 16027.3 | 143 | 0.9 | [0.8-1.1] |
|  |  | Asia-Pacific | 4707.6 | 15 | 0.3 | [0.2-0.5] |
|  |  | CCASAnet | 1054.6 | 1 | 0.1 | [0.0-0.7] |
|  |  | Central Africa | 2249.5 | 15 | 0.7 | [0.4-1.1] |
|  |  | East Africa | 5088.6 | 103 | 2.0 | [1.7-2.5] |
|  |  | South Africa | 2927.0 | 9 | 0.3 | [0.2-0.6] |

^*^ IR: incidence rate per 100 person years; ^†^ 95%CI: 95% confidence interval

**Table N- continued**

| **Age (years)** | **CD4** | **Region** | **Person-time (years)** | **Number of events** | **IR* (per 100 PY)** | **95% CI†** |
| --- | --- | --- | --- | --- | --- | --- |
| 15 - 19 | <200 | Overall | 341.5 | 4 | 1.2 | [0.4-3.1] |
|  |  | Asia-Pacific | 108.8 | 3 | 2.8 | [0.9-8.5] |
|  |  | CCASAnet | 112.4 | 0 | 0.0 | - |
|  |  | Central Africa | 11.5 | 0 | 0.0 | - |
|  |  | East Africa | 68.4 | 1 | 1.5 | [0.2-10.4] |
|  |  | South Africa | 40.5 | 0 | 0.0 | - |
|  | 200-499 | Overall | 1165.4 | 10 | 0.9 | [0.5-1.6] |
|  |  | Asia-Pacific | 430.2 | 1 | 0.2 | [0.0-1.7] |
|  |  | CCASAnet | 171.7 | 0 | 0.0 | - |
|  |  | Central Africa | 157.0 | 4 | 2.5 | [1.0-6.8] |
|  |  | East Africa | 248.8 | 5 | 2.0 | [0.8-4.8] |
|  |  | South Africa | 157.6 | 0 | 0.0 | - |
|  | ≥ 500 | Overall | 3163.2 | 14 | 0.4 | [0.3-0.7] |
|  |  | Asia-Pacific | 1343.6 | 6 | 0.4 | [0.2-1.0] |
|  |  | CCASAnet | 421.1 | 0 | 0.0 | - |
|  |  | Central Africa | 428.1 | 4 | 0.9 | [0.4-2.5] |
|  |  | East Africa | 428.4 | 3 | 0.7 | [0.2-2.2] |
|  |  | West Africa | 0.0 | 0 | 0.0 | - |
| 20 - 24 | <200 | Overall | 42.5 | 0 | 0.0 | - |
|  |  | Asia-Pacific | 7.6 | 0 | 0.0 | - |
|  |  | CCASAnet | 29.8 | 0 | 0.0 | - |
|  |  | Central Africa | 0.0 | 0 | 0.0 | - |
|  |  | East Africa | 0.0 | 0 | 0.0 | - |
|  |  | South Africa | 5.1 | 0 | 0.0 | - |
|  | 200-499 | Overall | 44.0 | 0 | 0.0 | - |
|  |  | Asia-Pacific | 11.6 | 0 | 0.0 | - |
|  |  | CCASAnet | 27.6 | 0 | 0.0 | - |
|  |  | Central Africa | 0.9 | 0 | 0.0 | - |
|  |  | East Africa | 1.4 | 0 | 0.0 | - |
|  |  | South Africa | 2.6 | 0 | 0.0 | - |
|  | ≥ 500 | Overall | 119.4 | 0 | 0.0 | - |
|  |  | Asia-Pacific | 53.6 | 0 | 0.0 | - |
|  |  | CCASAnet | 47.0 | 0 | 0.0 | - |
|  |  | Central Africa | 3.3 | 0 | 0.0 | - |
|  |  | East Africa | 1.6 | 0 | 0.0 | - |
|  |  | South Africa | 13.9 | 0 | 0.0 | - |

^*^ IR: incidence rate per 100 person years; ^†^ 95%CI: 95% confidence interval

**Figure A. Incidence rates of pre-ART mortality by region and time-updated age, IeDEA multiregional cohort, 2004-2016.**

**
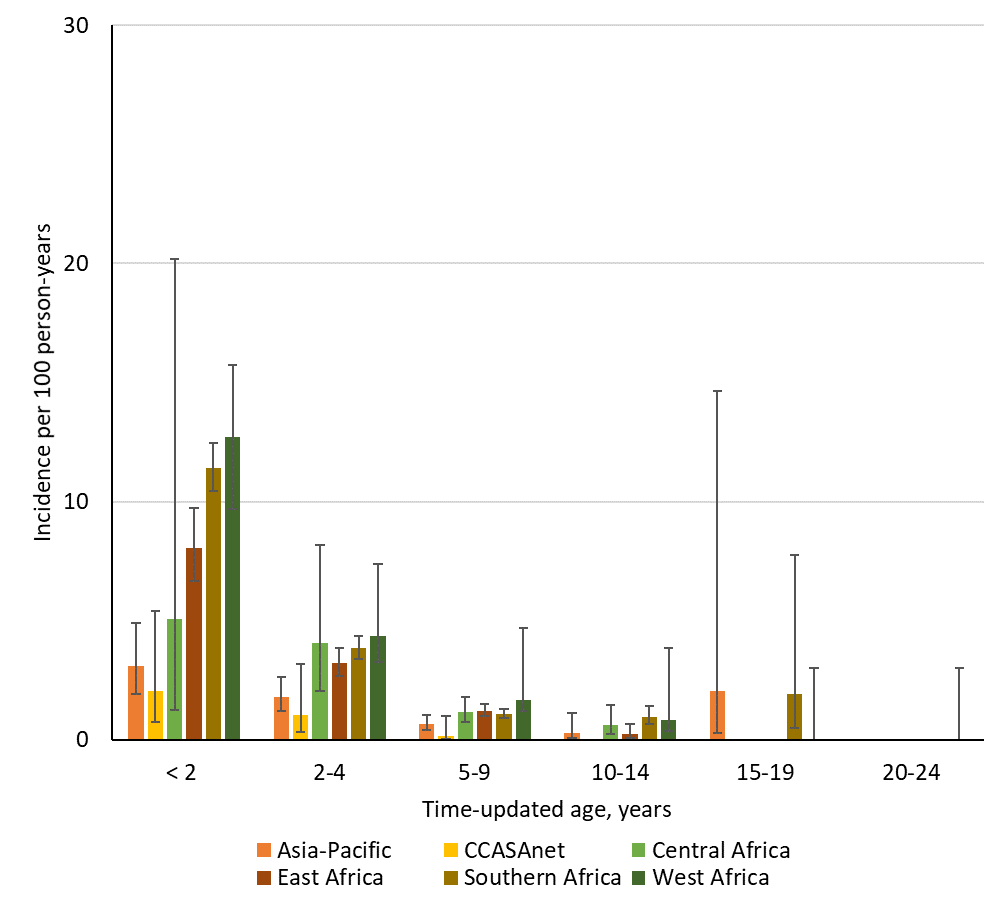
**

**Figure B. Frequency of first occurrence of WHO-4 (A) and WHO-3 (B) events in the pre-ART and post-ART periods, IeDEA multiregional cohort, 2004-2016**


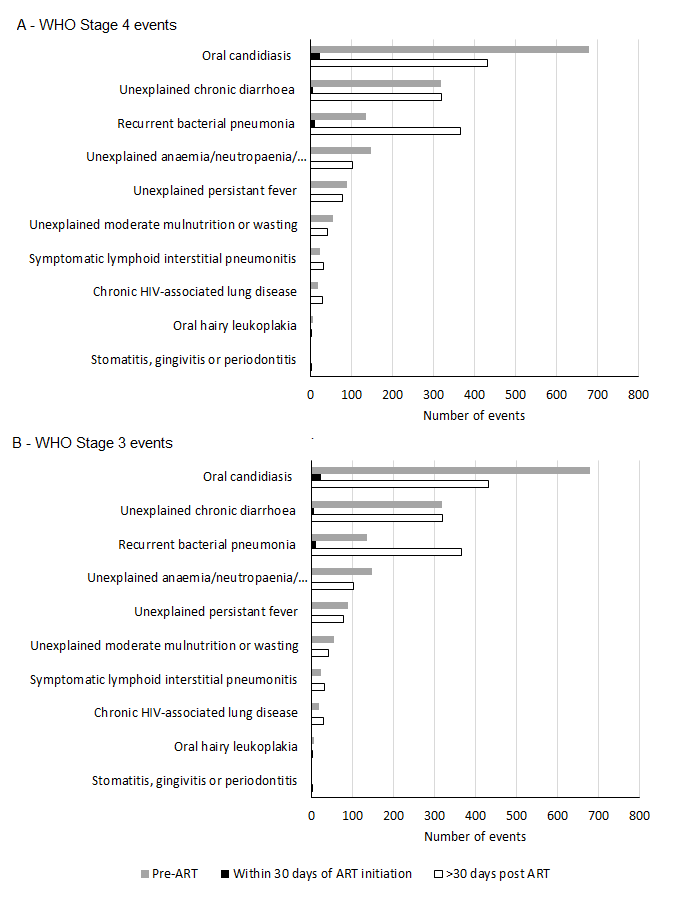


*Participants with a prevalent WHO-4 or WHO-3 event were excluded from the analysis of first occurrence of WHO-4 or WHO-3 events, respectively. Those with a prevalent WHO-3 event were included in the analysis of WHO-4 event and vice versa.*

**Figure C. Incidence rate of first occurrence of pre-ART WHO-4 event by region and time-updated age, IeDEA multiregional cohort, 2004-2016.**

*
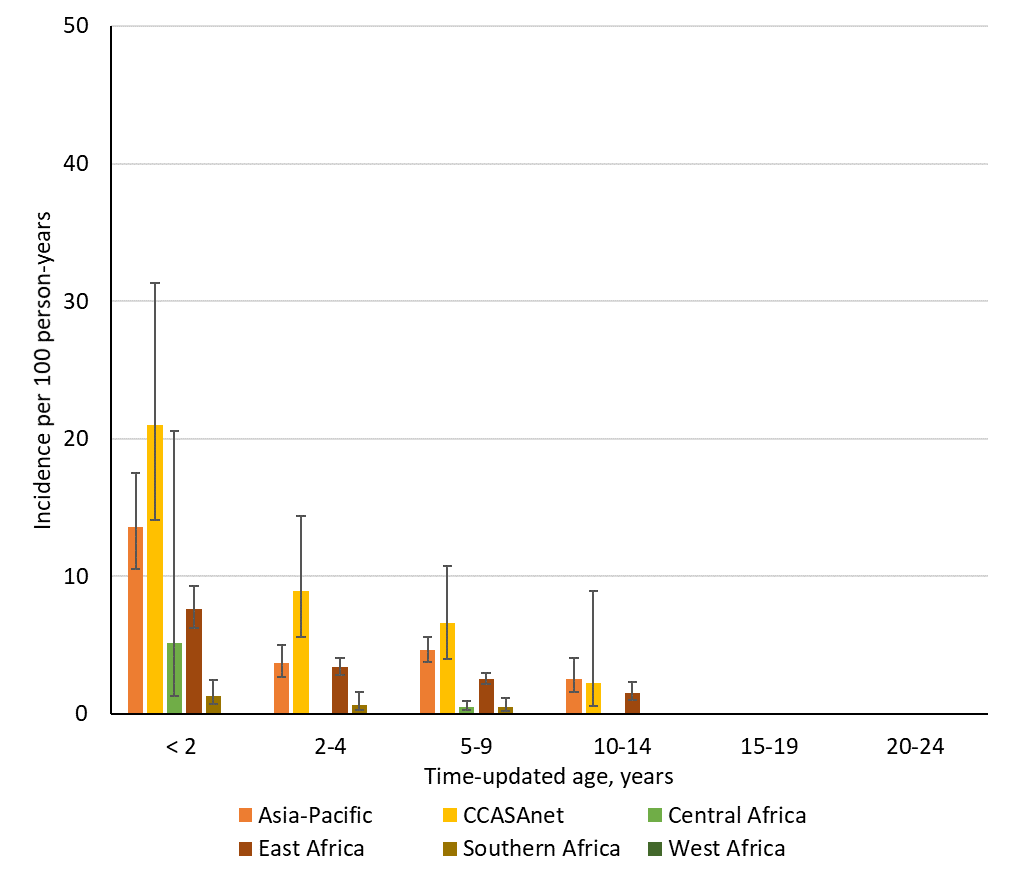
*

**Figure D. Incidence rate of first occurrence of pre-ART WHO-3 event by region and time-updated age, IeDEA multiregional cohort, 2004-2016.**

*
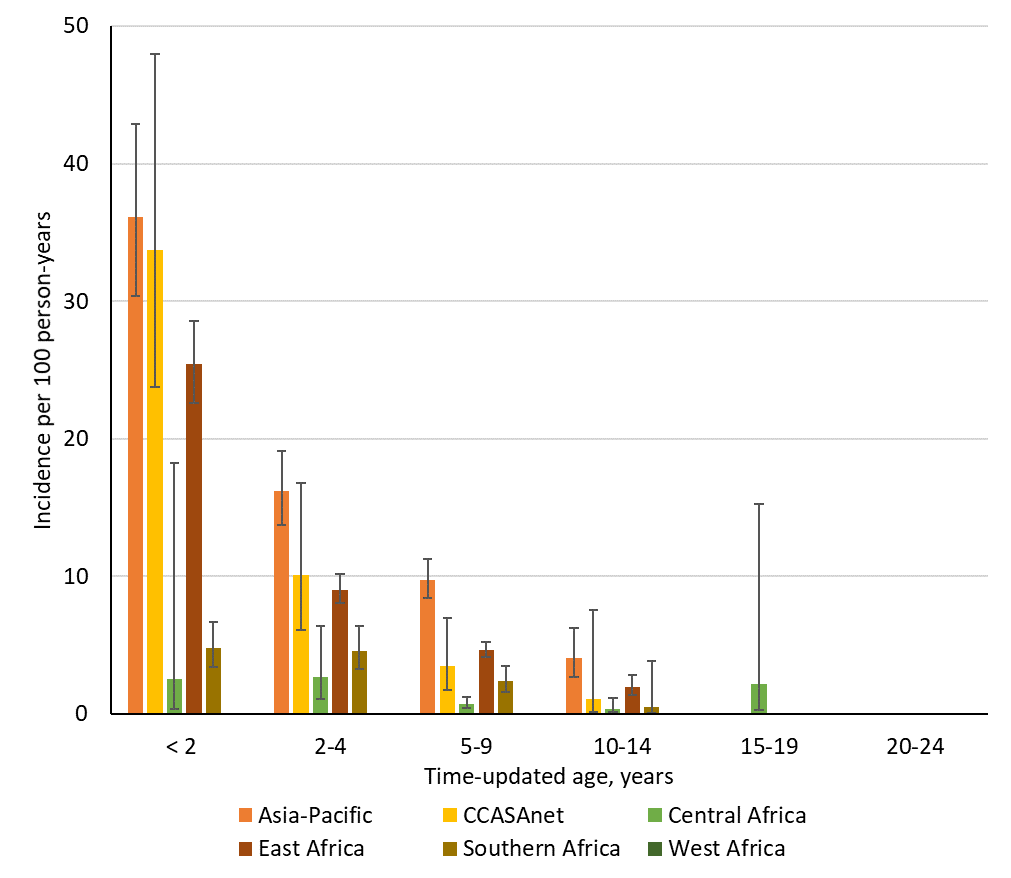
*

**Figure E. Incidence rates of post-ART mortality by region and time-updated age, IeDEA** *
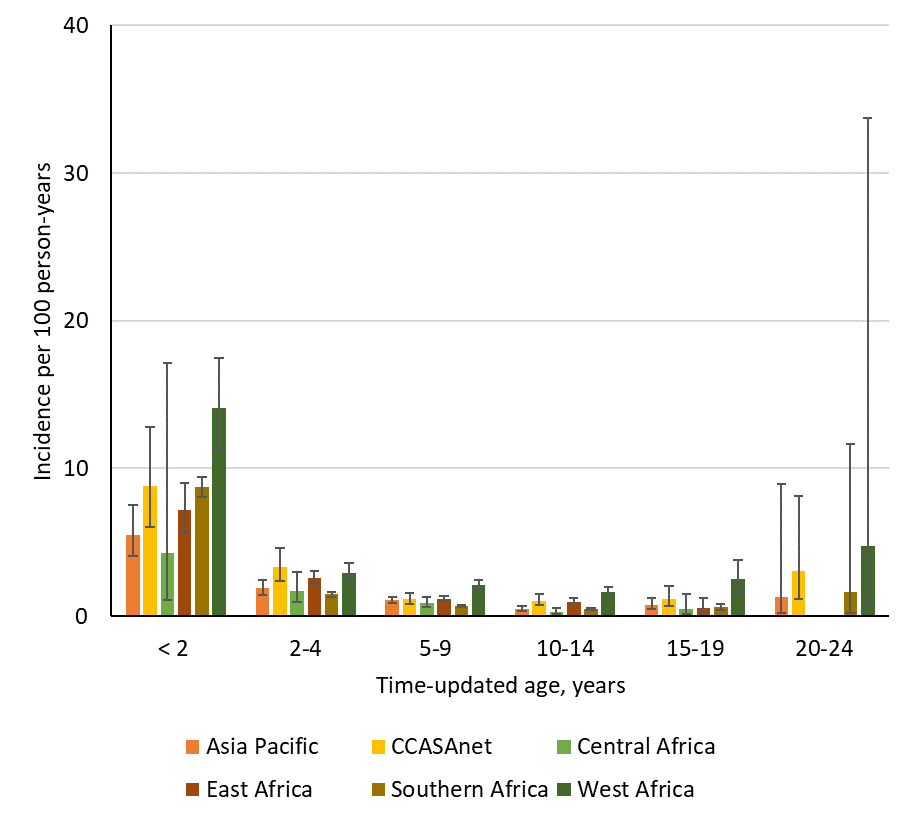
***multiregional cohort, 2004-2016.**

**Figure F. Incidence rate of first occurrence of post-ART WHO-4 event by region and time-updated age, IeDEA multiregional cohort, 2004-2016.**

*
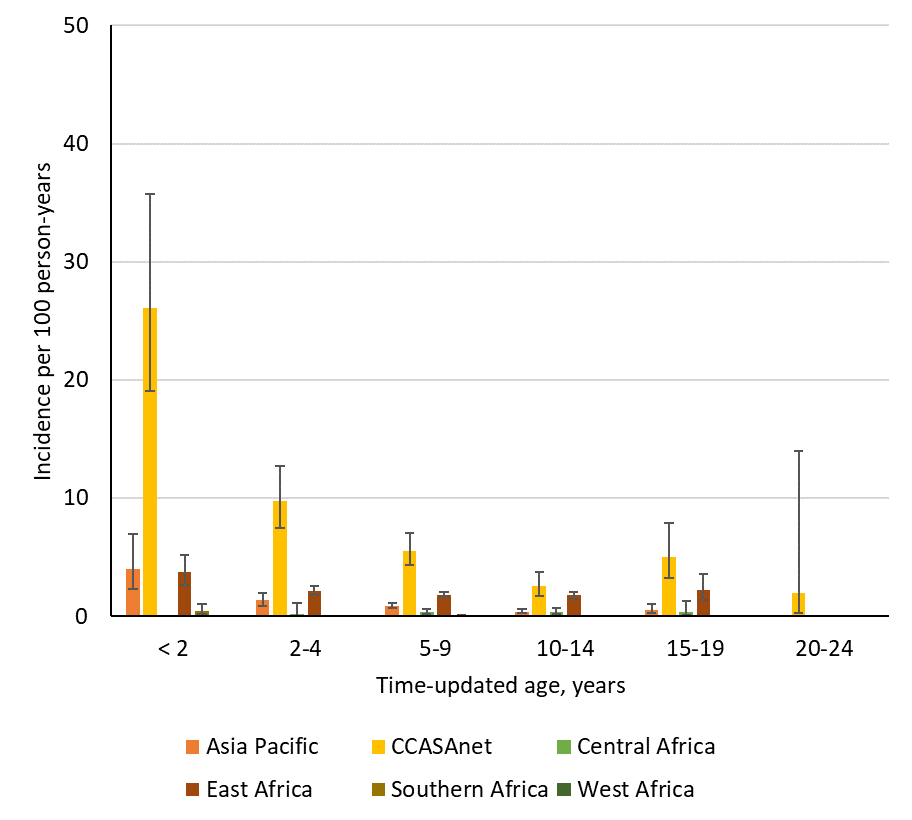
*

**Figure G. Incidence rate of first occurrence of post-ART WHO-3 event by region and time-updated age, IeDEA multiregional cohort, 2004-2016.**

**
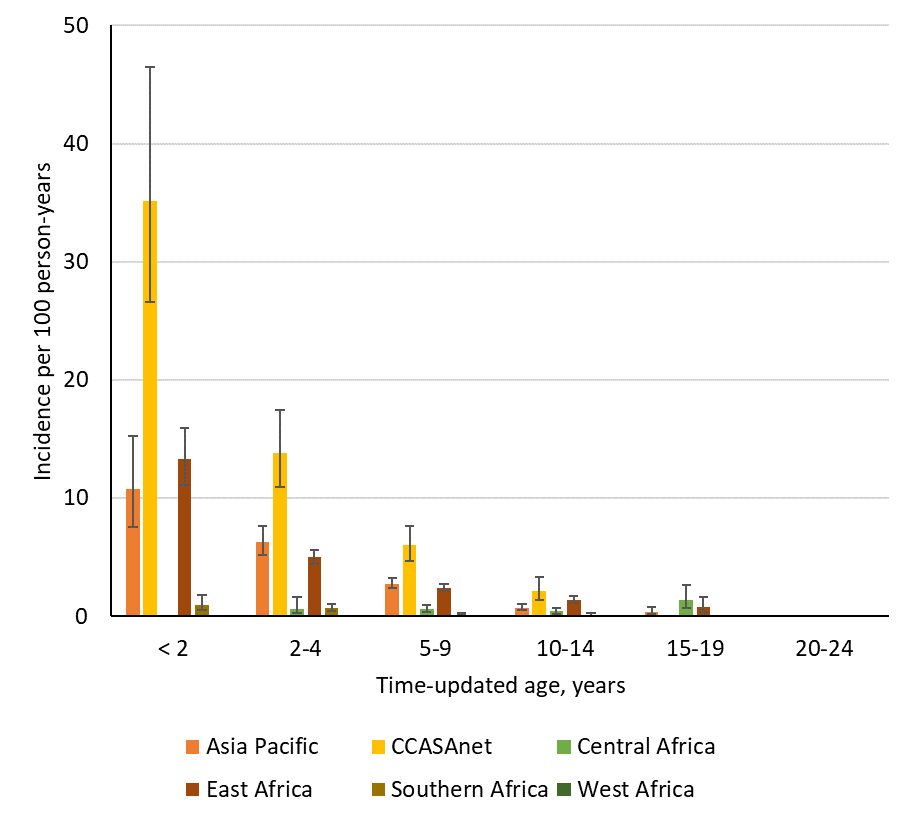
**
